# Ag_2_WO_4_ nanocatalyst-driven green synthesis of pyrano[2,3-*d*]pyrimidinones: an integrated experimental, DFT, and cytotoxic investigation

**DOI:** 10.1039/d5ra08635c

**Published:** 2026-01-16

**Authors:** Sathiaseelan Perumal, Perumal Muthuraja, M. Sasikumar, R. Hari Krishna, Paramasivam Manisankar, Viswanathan Subramanian

**Affiliations:** a Department of Industrial Chemistry, Alagappa University Karaikudi Tamil Nadu 630 006 India manisankarp@alagappauniversity.ac.in rsviswa@gmail.com; b Department of Chemistry, Bishop Heber College Tiruchirappalli Tamil Nadu 620 017 India; c Department of Chemistry, Indian Institute of Technology Guwahati Guwahati 781039 India; d Department of Chemistry, M.S. Ramaiah Institute of Technology Bangalore-560 054 India

## Abstract

The development of environmentally sustainable routes for synthesizing bioactive heterocycles remains a central objective in contemporary medicinal chemistry. In this work, silver tungstate (Ag_2_WO_4_) nanoparticles were synthesized and characterized by X-ray diffraction and scanning electron microscopy, confirming their crystalline structure and surface morphology. The prepared nanoparticles served as an efficient heterogeneous nanocatalyst for the one-pot green synthesis of pyrano[2,3-*d*]pyrimidinone derivatives (2a–2h) in an ethanol–water medium, affording excellent yields under mild conditions and demonstrating high catalytic efficiency with strong alignment to green chemistry principles. Comprehensive *in silico* analyses, including density functional theory (DFT), molecular docking, molecular dynamics (MD) simulations, and ADME screening, were performed to elucidate the electronic distribution, binding interactions, and pharmacokinetic profiles of the synthesized scaffolds. Docking studies revealed that compounds 2g (−7.58 kcal mol^−1^) and 2h (−7.34 kcal mol^−1^) exhibited binding affinities comparable to the reference drug afatinib (−8.01 kcal mol^−1^). *In vitro* cytotoxic evaluation against A549 lung carcinoma cells further identified compound 2h as the most potent derivative (IC_50_ = 39.29 µM), significantly outperforming the unsubstituted analogue 2a (IC_50_ = 120.65 µM). Overall, this integrated experimental, computational, and biological study establishes Ag_2_WO_4_ as a robust and sustainable nanocatalyst, offering an efficient pathway for the rapid synthesis of pyrano[2,3-*d*]pyrimidinone scaffolds with promising anticancer potential.

## Abstract 2

Ag_2_WO_4_ nanoparticles catalyse a green multicomponent synthesis of pyrano[2,3-*d*]pyrimidinones in aqueous ethanol, supported by DFT, molecular docking, MD simulations, ADME profiling, and *in vitro* cytotoxicity against A549 cells.

## Graphical abstract

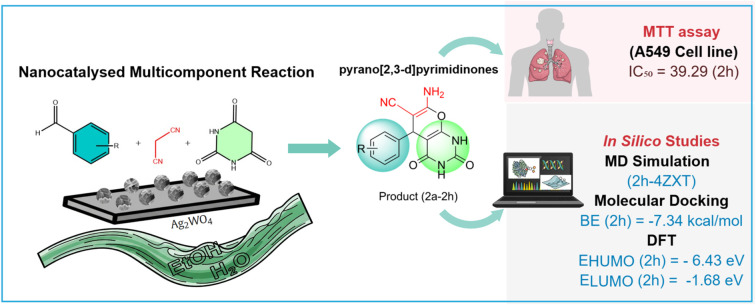

## 1 Introduction

The pyrano-pyrimidinone moiety is a significant class of fused heterocycles with diverse biological activities, such as antioxidant,^1^ antimicrobial,^2^ anti-inflammatory,^3^ and anticancer^4^ properties, making them attractive scaffolds for therapeutic development.^5^ Owing to their structural diversity and biological relevance, the development of efficient and sustainable methods for synthesising these frameworks is a priority in medicinal chemistry.

To date, various methodologies have been developed for constructing the pyrano[2,3-*d*]pyrimidinone core.^6–15^ Multicomponent reactions (MCRs) are eco-friendly owing to their high atom economy, operational simplicity, and ability to produce complex heterocycles in a single step.^16,17^ Simultaneously, nanocatalysts have gained popularity in organic synthesis because of their improved surface reactivity and reusability, thus adhering to green chemistry.^18,19^ However, innovative catalytic systems that offer both high efficiency and environmental friendliness are still required, particularly for pharmaceutically significant MCRs.

In this context, a review of the literature shows that silver tungstate (Ag_2_WO_4_) has been studied mainly for its redox activity and photocatalytic efficiency.^20^ However, reports on its application as a heterogeneous nanocatalyst in organic synthesis, particularly in multicomponent reactions leading to heterocycles, are still limited.^21^ These unexplored potential positions Ag_2_WO_4_ NPs as promising candidates for developing greener synthetic methodologies. Guo *et al.* demonstrated the bifunctional catalytic role of silver tungstate (Ag_2_WO_4_) in the carbonylation of terminal alkynes.^22^ Motivated by these findings, we synthesised Ag_2_WO_4_ nanoparticles (NPs) to further enhance their surface activity and extend the investigation.

This study introduces an environmentally benign one-pot protocol that utilises Ag_2_WO_4_ nanoparticles as heterogeneous nanocatalystss for the synthesis of pyrano[2,3-*d*]pyrimidinone derivatives. Importantly, the protocol proceeds efficiently under mild conditions, delivering excellent yields and purities within short reaction times without requiring chromatographic purification. By reducing energy consumption and waste, this methodology significantly enhances the green credentials of heterocyclic synthesis processes.

Building on this synthetic success, the compounds were systematically assessed to complement the experimental synthesis using a combination of computational and biological methods, such as density functional theory (DFT) calculations, ADME prediction, molecular docking, molecular dynamics (MD) simulations, MM-GBSA, and free energy landscape (FEL) mapping.^23^ Cytotoxicity was assessed in A549 lung cancer cells using an MTT assay.^24^ Afatinib, an FDA-approved EGFR tyrosine kinase inhibitor, was selected as the reference drug owing to its cytotoxicity in A549 cells.^25^ Although Afatinib primarily targets EGFR, its docking with PDB ID: 4ZXT (ERK2 kinase domain) was performed in this study as an exploratory benchmark, enabling a comparative analysis of the binding affinities of the clinically validated drug and the newly synthesised derivatives.

Overall, these integrated synthetic, computational, and biological approaches address the critical gap in the sustainable production of pharmaceutically important heterocycles. This study breaks new ground by utilising Ag_2_WO_4_ NPs in a one-pot method to synthesise pyrano[2,3-*d*]pyrimidinones. This highlights their potential as anticancer agents and supports ongoing initiatives for sustainable drug discovery.

## 2 Results and discussion

### 2.1 Chemistry

#### 2.1.1 Catalyst characterisation

**Fig. 1:**
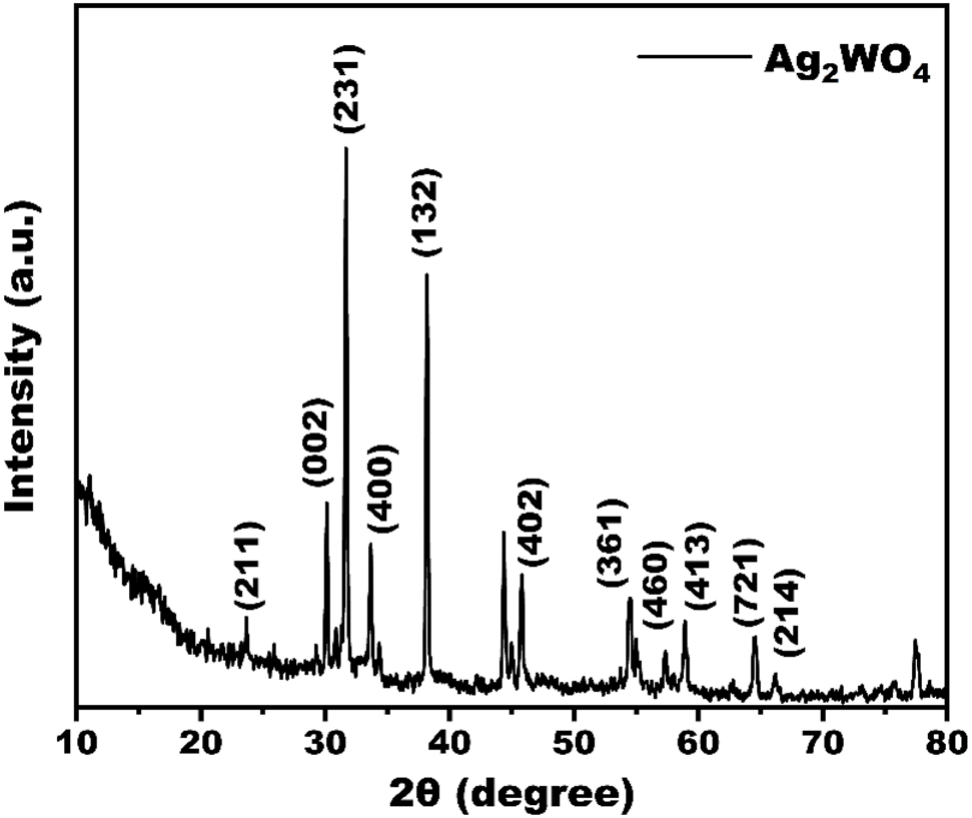
X-ray diffraction (XRD) pattern of synthesized Ag_2_WO_4_ nanocatalyst, showing characteristic diffraction peaks.

The crystalline phase of Ag_2_WO_4_ was analysed using X-ray diffraction. Fig. 1 shows the diffraction patterns of the prepared samples. The as-prepared Ag_2_WO_4_ exhibited characteristic diffraction peaks at 24.2°, 30.5°, 31.5°, 34.2°, 38.5°, 46.7°, 54.2°, 57.2°, 59.2°, 64.4°, and 66.2°, corresponding to the (211), (002), (231), (400), (132), (402), (361), (460), (413), (721), and (214) planes of Ag_2_WO_4_ (JCPDS card no.00-034-0061), indicating the successful formation of Ag_2_WO_4_. The presence of narrow and intense diffraction peaks indicates that Ag_2_WO_4_ is highly crystalline. Nonetheless, minor traces of residue or unreacted silver were found in the Ag_2_WO_4_ powder sample. The average crystallite size was 47 nm.

**Fig. 2:**
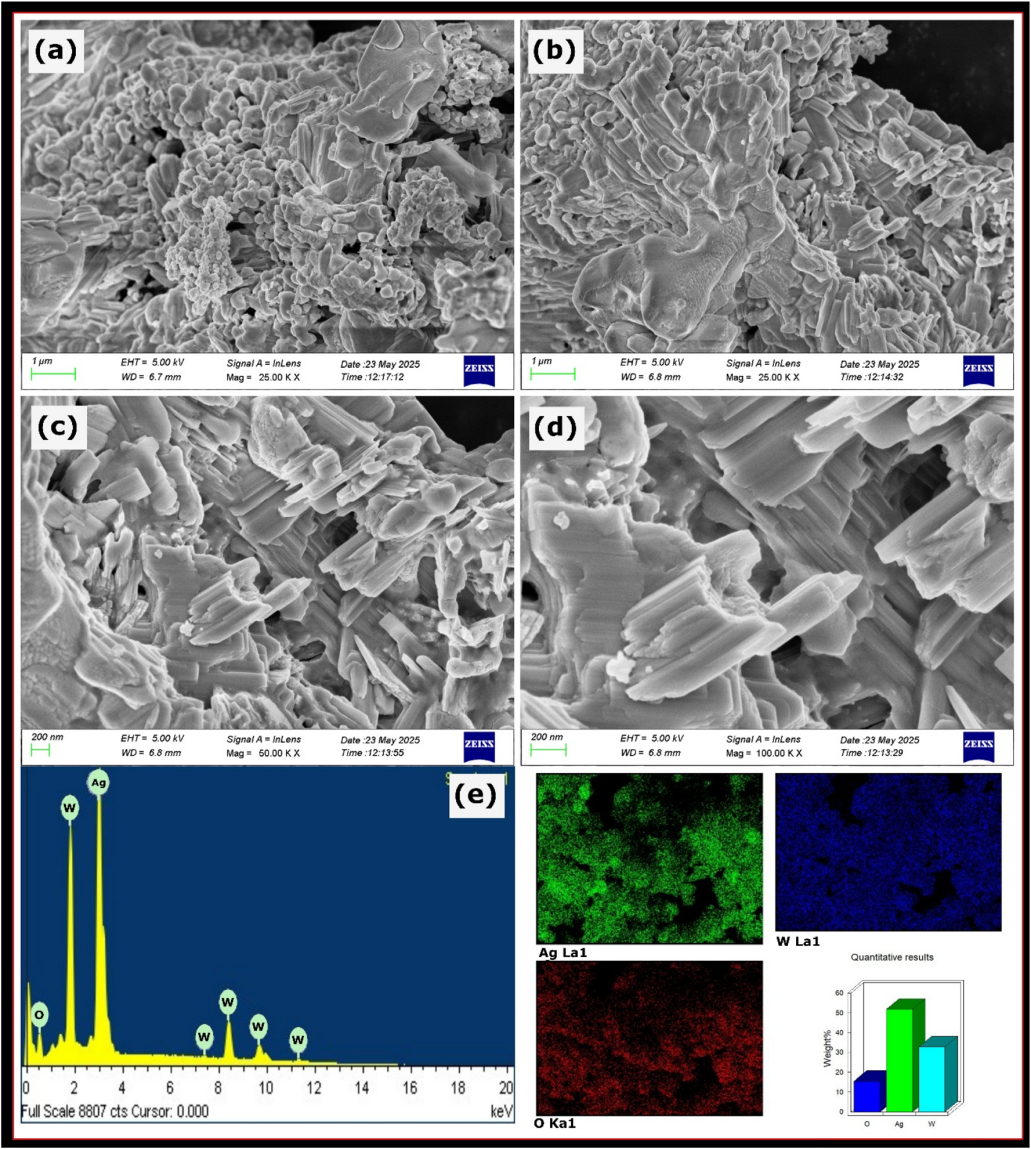
SEM images of Ag_2_WO_4_ at different magnifications: (a and b) low magnification showing agglomerated particle clusters, and (c and d) higher magnification revealing fused rod-like structures with uneven growth and rough surfaces. (e) EDAX spectrum confirming the presence of Ag, W, and O without any impurities. Elemental mapping images (Ag, W, O).

Scanning electron micrographs of Ag_2_WO_4_ at different magnifications are shown in Fig. 2a–d. At lower magnification, the surface morphology was found to be a highly agglomerated mass of particle clusters. A closer observation of the micrographs revealed that the agglomerated particles had a mixed morphology consisting of irregular particle aggregates and fused rod-like structures (Fig. 2b). However, at higher magnifications, the SEM images clearly show the dominance of rod-like fused structures with uneven growth. This uneven growth results in a rough surface, and the tailing of rod-like structures can be observed. Furthermore, to evaluate the elemental composition and distribution, EDAX analysis with colour mapping was performed, as shown in Fig. 2. The EDAX and colour mapping results confirmed that, except for Ag, W, and O, no other elements were present in the compound, suggesting simple purity of the sample. Furthermore, colour mapping showed a uniform distribution of elements throughout the product and the homogeneous nature of the product.

#### 2.1.2 Catalytic efficiency of Ag_2_WO_4_ NPs in Pyrano[2,3-*d*]pyrimidinone synthesis

After examining the structure and morphology of the Ag_2_WO_4_ nanoparticles using X-ray diffraction (XRD), Scanning Electron Microscopy (SEM) with Energy Dispersive X-ray Analysis (EDX) analyses, their catalytic potential in the one-pot synthesis of pyrano[2,3-*d*]pyrimidinone derivatives was evaluated. The effectiveness of the Ag_2_WO_4_ nanocatalyst was compared with that of other reported catalysts used in the transformation (Table S17). The majority of heterogeneous systems have produced products in good-to-excellent yields (80–97%); however, the majority of the above-mentioned systems require longer reaction times, higher temperatures, or specific equipment to effect the desired transformations. Although some studies have described the use of greener media, such as water or ethanol, for the reaction of interest, it is clear that most reactions are slow to reach completion.

The Ag_2_WO_4_ catalyst demonstrated superior performance compared with previously reported catalysts for the synthesis of pyrano[2,3-*d*]pyrimidinone derivatives. Specifically, Ag_2_WO_4_ catalysed the one-pot synthesis of pyrano[2,3-*d*]pyrimidinones in an EtOH/H_2_O (1 : 1) solvent mixture, achieving product yields of 95% within a reaction time of 5–8 min at 70 °C. The superior performance of Ag_2_WO_4_ can be attributed to the operational simplicity and rapidity of the catalyst and/or its adherence to the principles of green chemistry. Overall, the results demonstrated that Ag_2_WO_4_ is a highly active and environmentally compliant catalyst that can match or exceed conventional systems in terms of activity and practicability.

#### 2.1.3 Proposed mechanism and substrate scope

Based on the literature^22^ and the observed efficiency of the transformation, a plausible mechanism for the Ag_2_WO_4_-nanocatalyzed synthesis of pyrano[2,3-*d*]pyrimidinone derivatives is proposed in Scheme 2. A distinguishing feature of this methodology is the dual catalytic nature of Ag_2_WO_4_ NPs, which provides both Lewis acidic (Ag^+^) and Brønsted basic (WO_4_^2−^) sites in the catalyst structure. While Ag^+^ cations coordinate with the aldehyde carbonyl oxygen to enhance the electrophilicity of the aldehyde carbon, the tungstate anions simultaneously act as a base and abstract the acidic protons present in malano nitrile and barbituric acid in the subsequent steps of the proposed mechanism. The malononitrile carbanion attacks the aldehyde carbon, forming a benzylidenemalononitrile intermediate *via* Knoevenagel condensation reaction. The newly formed enolate of barbituric acid acts as a nucleophile and attacks the electrophilic center of the benzylidenemalononitrile intermediate to form a Michael-type adduct. The Michael-type adduct then undergoes intramolecular ring closure to form a fused pyranopyrimidinone, which can then undergo a subsequent tautomeric transformation to give the final product. The cooperative action of the acid-base functional groups on the Ag_2_WO_4_ NP significantly reduced the energy barrier for the reaction and facilitated the complete reaction within a few minutes at low temperatures and high yields.

Table 1 lists the generality of the proposed method. All aromatic aldehydes examined, regardless of whether they had an electron-donating or electron-withdrawing group, underwent a clean reaction to produce the corresponding derivatives (2a–2h) in very high yields (84–95%) over a short time period (5–7 min). Thus, the unsubstituted analogue (2a) was produced in 95% yield (7 min), while *p*-chloro (2d) and *p*-fluoro (2g) analogues were produced in 91% and 95% yields, respectively, within 6 min. Additionally, the *o*-dichloro-substituted analogue (2h) was produced in 88% yield within 7 min, demonstrating the wide applicability of the Ag_2_WO_4_-NP-based system. In summary, the proposed mechanism and scope demonstrate that Ag_2_WO_4_ nanoparticles act as a bifunctional nanocatalyst to enable a green precipitation-assisted protocol for the facile and efficient synthesis of pyrano[2,3-*d*]pyrimidinones.

**Table 1:** Representative library of synthesized pyrano[2,3-*d*]pyrimidinone derivatives (2a–2h) obtained using Ag_2_WO_4_ catalyst under optimized EtOH/H_2_O conditions, with isolated yields and reaction times indicated

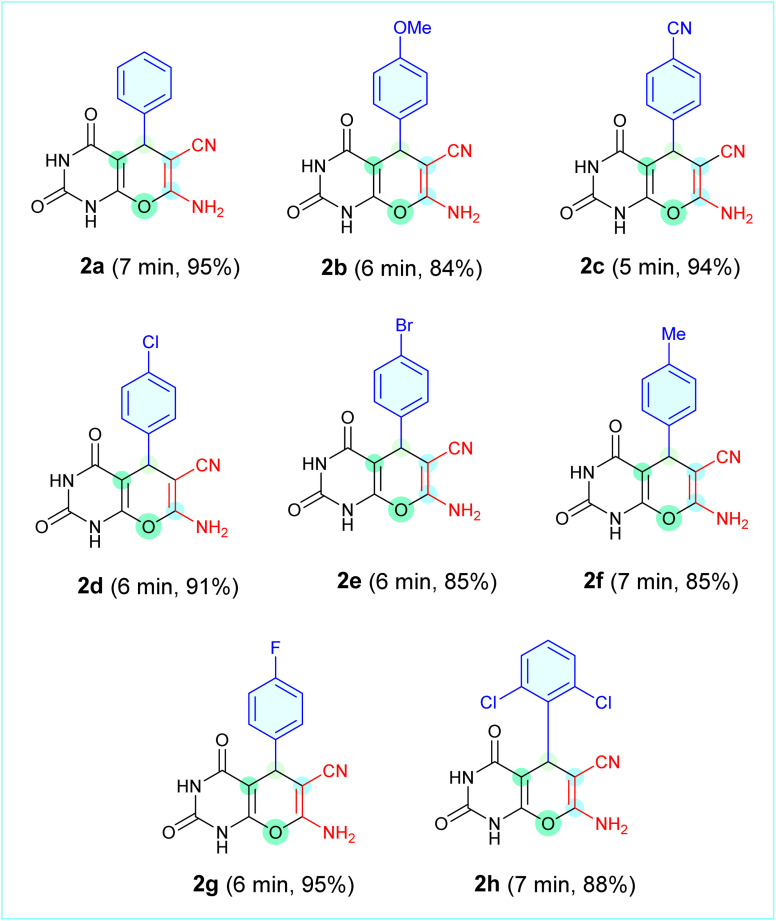

#### 2.1.4 NMR characterisation of pyrano[2,3-*d*]pyrimidinone derivatives

**Fig. 3:**
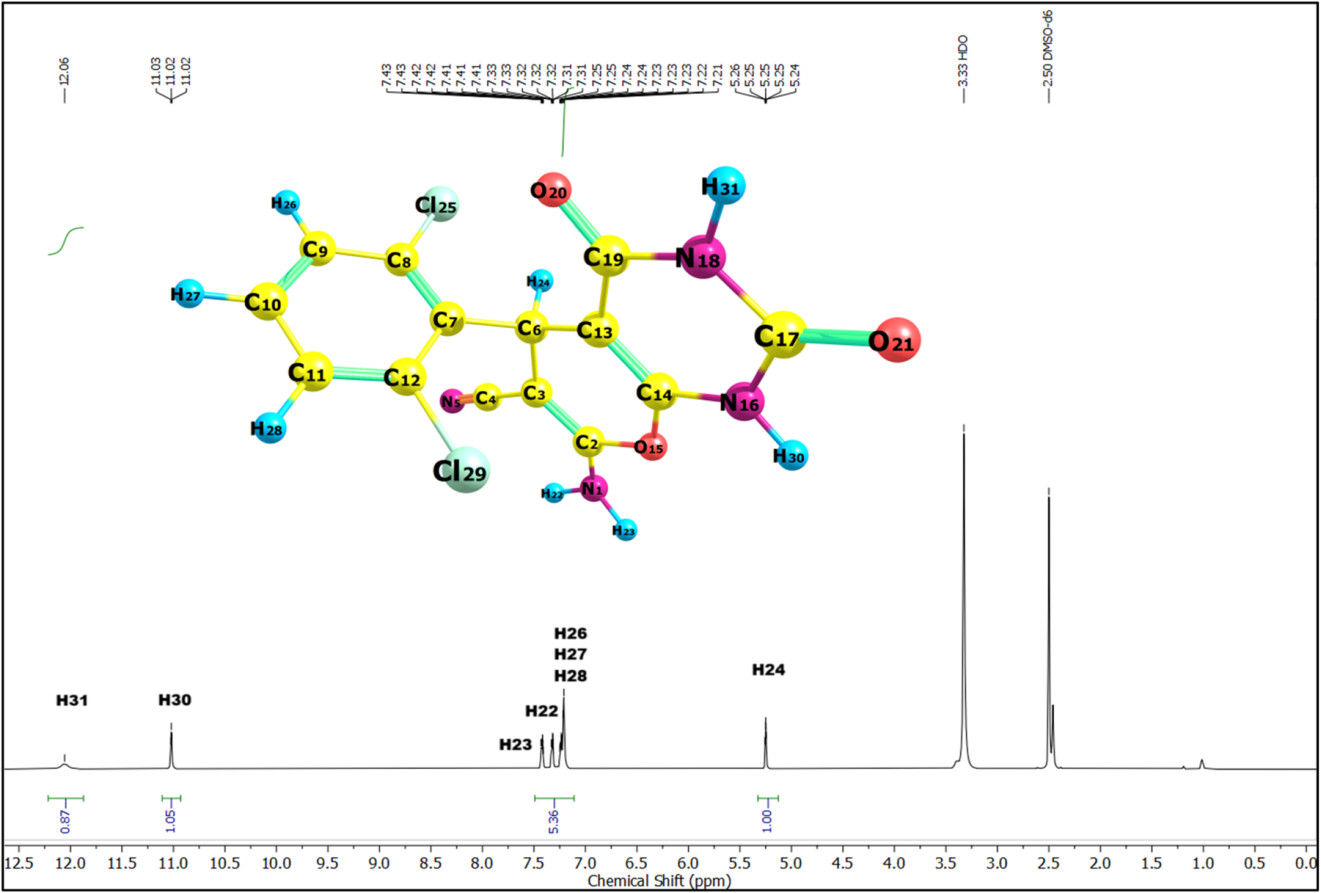
^1^H NMR spectrum (in DMSO) of compound 2h recorded at 600 MHz, showing the chemical shifts (*δ*, ppm) with proton assignments corresponding to the molecular structure.

**Fig. 4:**
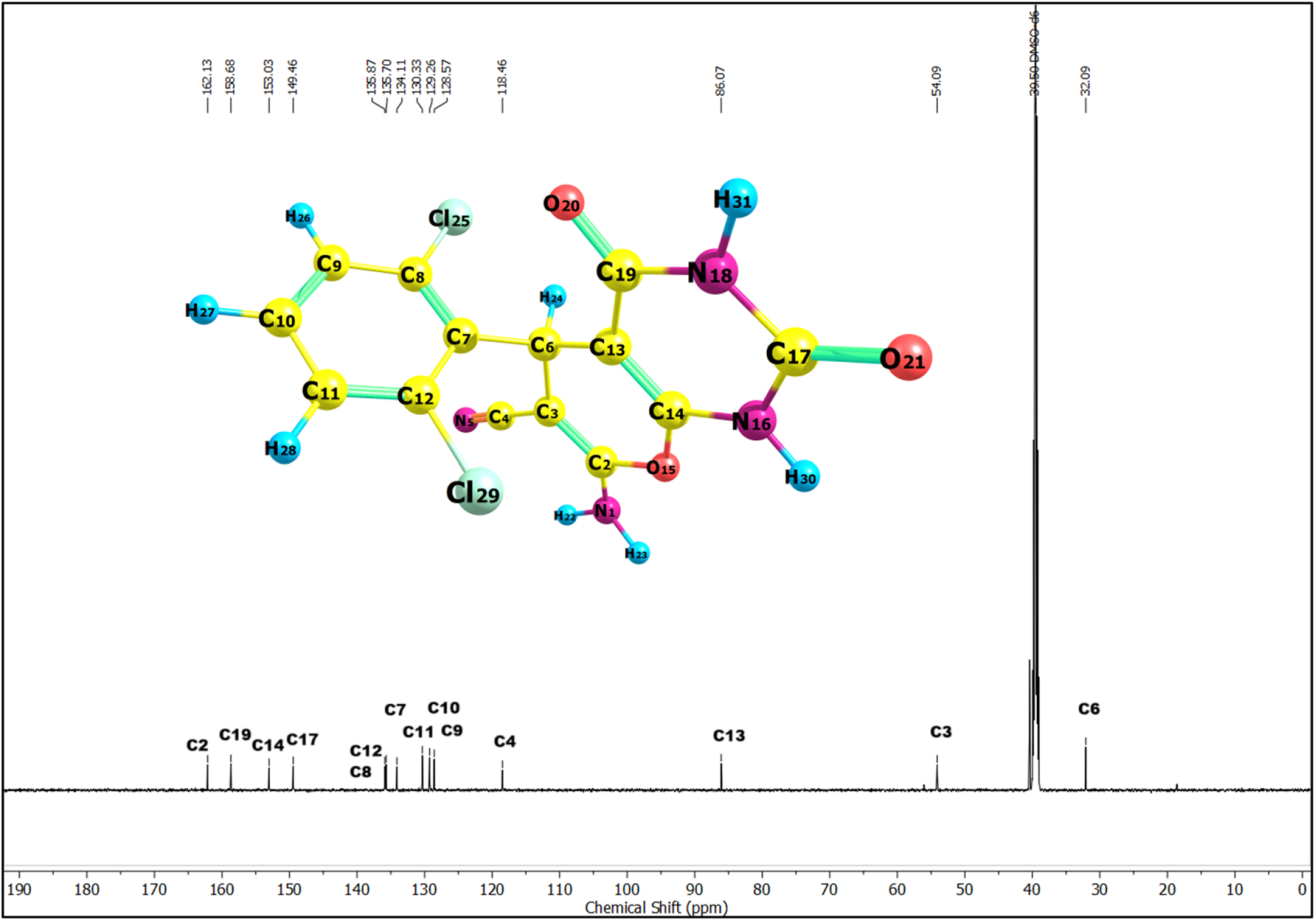
^13^C NMR spectrum (in DMSO) of compound 2h recorded at 600 MHz, showing the chemical shifts (*δ*, ppm) with carbon assignments corresponding to the molecular structure.

Following the confirmation of the catalytic efficiency of Ag_2_WO_4_ in the synthesis of pyrano[2,3-*d*]pyrimidinones, the structural integrity of the synthesised derivatives (2a–2h) was validated. A comprehensive spectroscopic characterisation was conducted, and NMR and IR analyses provided conclusive evidence for the successful formation of the intended heteroaromatic scaffolds. The ^1^H and ^13^C NMR spectra of the synthesised scaffolds (2a–2h) provided compelling evidence for the successful formation of heteroaromatic scaffolds. The representative ^1^H and ^13^C NMR spectra of compound 2h are shown in Fig. 3 and 4, respectively, and the complete experimental NMR and IR spectra of all derivatives (2a–2h) are provided in the SI (Fig. S1–S24). In the ^1^H NMR spectra, we observed two strongly deshielded singlets at *δ* ∼12.0 and *δ* ∼11.0 ppm, respectively. This downfield shift was attributed to the cyclic imide environment formed when the nitrogen atom in the six-membered pyrimidinone ring was flanked by two carbonyl groups.

Such glutarimide-type deshielding significantly lowers the shielding of the N–H protons, leading to resonances far from downfield. In compound 2h, these signals were assigned to H31 (*δ* 12.06 ppm, amide N–H) and H30 (*δ* 11.02 ppm, imide N–H). The –NH_2_ protons at C2 appeared as a broad signal at *δ* ∼7.4 ppm, confirming the presence of an exocyclic amino group. The methine proton (H24) attached to C6 of the pyran ring appeared at *δ* ∼5.2 ppm, serving as a diagnostic marker for ring closure. The aromatic protons (H26–H28) of the phenyl substituent were observed at *δ* 7.2–7.3 ppm, with minor chemical shift variations across the derivatives reflecting the electronic influence of substituents such as –Cl, –Br, –F, –CH_3_, –OCH_3_, and –CN. This overall spectral pattern, such as two downfield NH signals, a mid-field amino group, a methine resonance near *δ* 4.0–5.2 ppm, and aromatic protons near *δ* 7.2–7.3 ppm was consistently observed across all derivatives, confirming the integrity of the prano[2,3-*d*]pyrimidinone framework.

The ^13^C NMR spectra further substantiated these structural assignments. We observed the most deshielded signals with respect to the sp^2^ carbon (C2) of the pyran ring, which is connected to O15 and N1, followed by the carbonyl carbons of the pyrimidinone core, resonating in the *δ* 160–167 ppm range, which is consistent with conjugation and strong electron-withdrawing effects. In compound 2h, the characteristic carbonyl resonances were observed at *δ* = 158.68 and 149.46 ppm. The cyano carbon signal was consistently detected at *δ* 118 ppm (*δ* 118.46 ppm for 2h), serving as a reliable fingerprint of the –CN substitution at the pyran ring. The sp^2^ hybridised carbon atoms at the ring junction of the pyran ring appeared in the *δ* 50–90 ppm range (*δ* 54.09 and 86.07 ppm for 2h), while the sp^3^ carbon of the system resonated between *δ* 35–39 ppm (*δ* 32.09 ppm in 2h). The substituent effects are clearly reflected in the aromatic carbon region; electron-withdrawing groups, such as halogens (Cl, Br, and F), produced deshielding of the ortho and ipso carbons (*δ* ∼135–140 ppm), whereas electron-donating substituents (–OCH_3_ and –CH_3_) induced slight upfield shifts in the phenyl carbons.

In summary, experimental NMR data confirmed the target structure. The reproducible downfield NH resonances, characteristic methine and amino signals in the proton spectra, carbonyl and cyano resonances in the carbon spectra, and substituent-dependent aromatic shifts confirmed the successful synthesis of the scaffolds (2a–2h). To further validate the structural assignments, the experimental ^1^H and ^13^C NMR spectra of compound 2h were compared with the theoretical chemical shifts calculated at the B3LYP/def2-TZVP level of theory for the DMSO phase (Table 2). For ^1^H NMR, the calculated chemical shifts aligned with the experimental trend with good accuracy (RMSD = 2.6 ppm). The deshielded protons H31 (*δ*_exp = 12.0 ppm) and H30 (*δ*_exp = 11.0 ppm), arising from the glutarimide-type N–H groups, were predicted theoretically (*δ*_calc ∼7.0–7.1 ppm), albeit slightly underestimated because of the known limitations of DFT shielding calculations.

**Table 2:** Experimental and calculated ^1^H NMR and ^13^C NMR chemical shifts (*δ* in ppm) of compound 2h at the B3LYP/def2-TZVP level of theory in the DMSO phase

^1^H NMR			^13^C NMR		
Atom	B3LYP	Exp	Atom	B3LYP	Exp
H22	5.0	7.4	C6	45.2	32.1
H23	4.5	7.4	C3	65.6	54.1
H24	5.8	5.3	C13	100.8	86.1
H26	7.6	7.2	C4	130.9	118.5
H27	7.5	7.3	C9	139.7	128.6
H28	7.5	7.2	C10	139.8	129.3
H30	7.0	11.0	C11	141.7	130.3
H31	7.1	12.0	C7	151.4	134.1
RMSD	2.6		C12	152.7	135.7
			C8	153.8	135.9
			C17	156.9	149.5
			C14	167.6	153.0
			C19	170.9	158.7
			C2	176.4	162.1
			RMSD	12.7	

The methine proton H24 (attached to C6) appeared at *δ*_exp = 5.3 ppm, which closely matched the predicted value of *δ*_calc = 5.8 ppm. The aromatic protons H26–H28 clustered around *δ*_exp 7.2–7.4 ppm, also showing good correlation with the theoretical data *δ*_calc = 7.5–7.6 ppm. The ^13^C NMR results showed a good match between the experimental and theoretical shifts, with an RMSD of 12.7 ppm, which was acceptable. The carbonyl carbons (C17 and C19) resonated experimentally at *δ* 149.5 and 158.7 ppm, respectively, and were reproduced theoretically (*δ*_calc ∼157–171 ppm). The cyano carbon (C4) appeared at *δ*_exp = 118.5 ppm, in line with its strong electron-withdrawing environment, and was well predicted by DFT (*δ*_calc ≈ 130.9 ppm). The methine carbon (C6) gave a signal at *δ*_exp = 32.1 ppm (*δ*_calc = 45.2 ppm), which is consistent with the sp^3^-hybridised environment in the fused pyran ring. The most downfield carbon, C2 (*δ*_exp = 162.1 ppm), corresponds to the sp^2^-hybridised carbon in the pyran ring conjugated with adjacent heteroatoms and was predicted to be *δ*_calc = 176.4 ppm. The remaining aromatic carbons (C7–C12) resonated between *δ*_exp 126–136 ppm, with theoretical values showing good correspondence.

The strong correlation between the theoretical and experimental NMR results strongly supported the structural assignment and confirmed the theoretically predicted electronic environment of the molecules. The similar comparison table is also shown for each of the other derivatives 2a–2h in the SI (Tables S1–S16). This confirmed the assignments based on experimental NMR data for each derivative. In addition to NMR analysis, FT-IR analysis was performed to further validate the key functional groups and vibrational features of the synthesised scaffolds.

#### 2.1.5 IR characterisation of pyrano[2,3-*d*]pyrimidinone derivatives

The FT-IR spectra of the synthesised scaffolds (2a–2h) confirmed that the characteristic bands were consistent with those of the synthesised compounds. We observed broad bands in the 3400–3380 cm^−1^ region, attributable to primary amine (–NH_2_) stretching, and distinct bands around 3215–3180 cm^−1^ due to amide N–H stretching of the pyrimidinone core. The cyano group (C≡N) was consistently observed as a sharp, medium-intensity band at 2195–2200 cm^−1^, confirming the presence of nitrile functionality. Two strong bands corresponding to the C=O stretching vibrations of the pyrimidinone/lactam functional groups appeared in the range of 1720–1670 cm^−1^, which is characteristic of conjugated carbonyl groups within the fused heteroaromatic system.

Another characteristic band at 1380–1090 cm^−1^, assigned to the C–O–C stretching of the pyran ring, and absorptions in the fingerprint region (900–700 cm^−1^) correspond to the aromatic C–H bending modes. Substituent effects were also evident; halogenated derivatives (2a–2h) exhibited stronger bands in the 600–500 cm^−1^ region corresponding to C–Cl or C–Br stretching, while the methoxy-substituted compound (2b) showed an additional band near 1096 cm^−1^ characteristic of C–O stretching. The IR spectra of the synthesised derivatives (2a–2h are shown in the SI (Fig. S1–S32), with comprehensive FT-IR data for each derivative detailed in Sections 2.2.1–2.2.8.

**Fig. 5:**
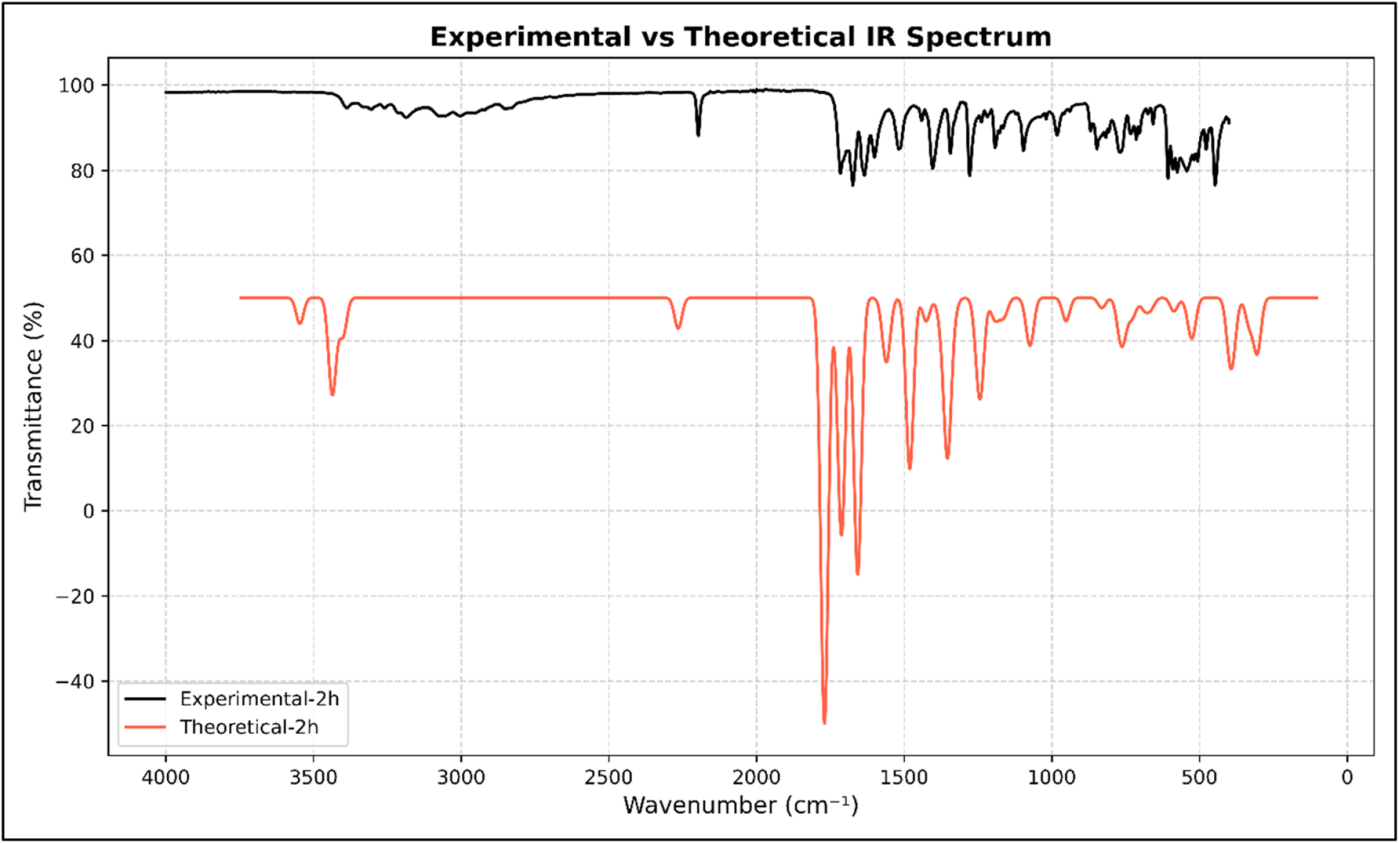
Comparative experimental and theoretical IR spectra of compound 2h.

The comparative IR analysis of compound 2h, as shown in Fig. 5, demonstrates excellent agreement between the experimental spectrum and the theoretically predicted vibrational profile, thereby validating the optimised geometry and electronic structure calculations. Experimentally, strong absorption bands were observed at ∼3389 and 3213 cm^−1^, corresponding to primary amino and amide N–H stretching vibrations respectively, which were reproduced in the theoretical spectrum with slight shifts due to anharmonic and solvation effects. The sharp absorption near 2196 cm^−1^ was assigned to the cyano (C≡N) stretch, which was consistent with the experimental and theoretical results. In the carbonyl region, distinct stretching bands were observed at approximately 1717 and 1673 cm^−1^, which correlated well with the theoretically predicted C=O vibrations, confirming the integrity of the pyranone and pyrimidinone frameworks. Additionally, a C–O–C stretching band appeared near 1096 cm^−1^ in both spectra, highlighting the characteristic contribution of the heterocyclic oxygen.

The close correspondence between the theoretical and experimental spectra not only supports the structural assignment of 2h but also reinforces the reliability of the DFT-based vibrational analysis for the entire series of derivatives (2a–2h). This similarity provides confidence in linking computational insights to experimental validation. Although 2h is shown as a representative example, comparative experimental and theoretical IR spectra of the derivatives (2a–2h) are presented in the SI (Fig. S25–S32), thus validating the characteristic vibrational features.

### 2.2 Computational analysis

Following the synthesis and characterisation of pyrano[2,3-*d*]pyrimidinones, we proceeded with the application of computational methods that would provide additional evidence and support the experimental data and explain the properties of compounds 2a–2h. The use of computational methods for this purpose provided an opportunity to utilise the DFT method to provide HOMO–LUMO, MEP, and other reactivity descriptors to illustrate the electronic distribution and reaction of derivatives 2a–2h. Additionally, docking of derivatives 2a–2h with PDB ID: 4ZXT using afatinib (A549 assay) as a reference drug provided an assessment of the binding affinities of the derivatives with the target protein. Dynamic stability was also investigated through 100 ns molecular dynamics simulations, which included additional analyses such as MM/GBSA,^26^ PCA and FEL.^27,28^ Finally, ADME prediction of the pharmacokinetic potential of derivatives 2a–2h was performed, and the results were correlated with MTT cytotoxicity to bridge the gap between computational methods and biological systems.

#### 2.2.1 Global reactive parameters

**Fig. 6:**
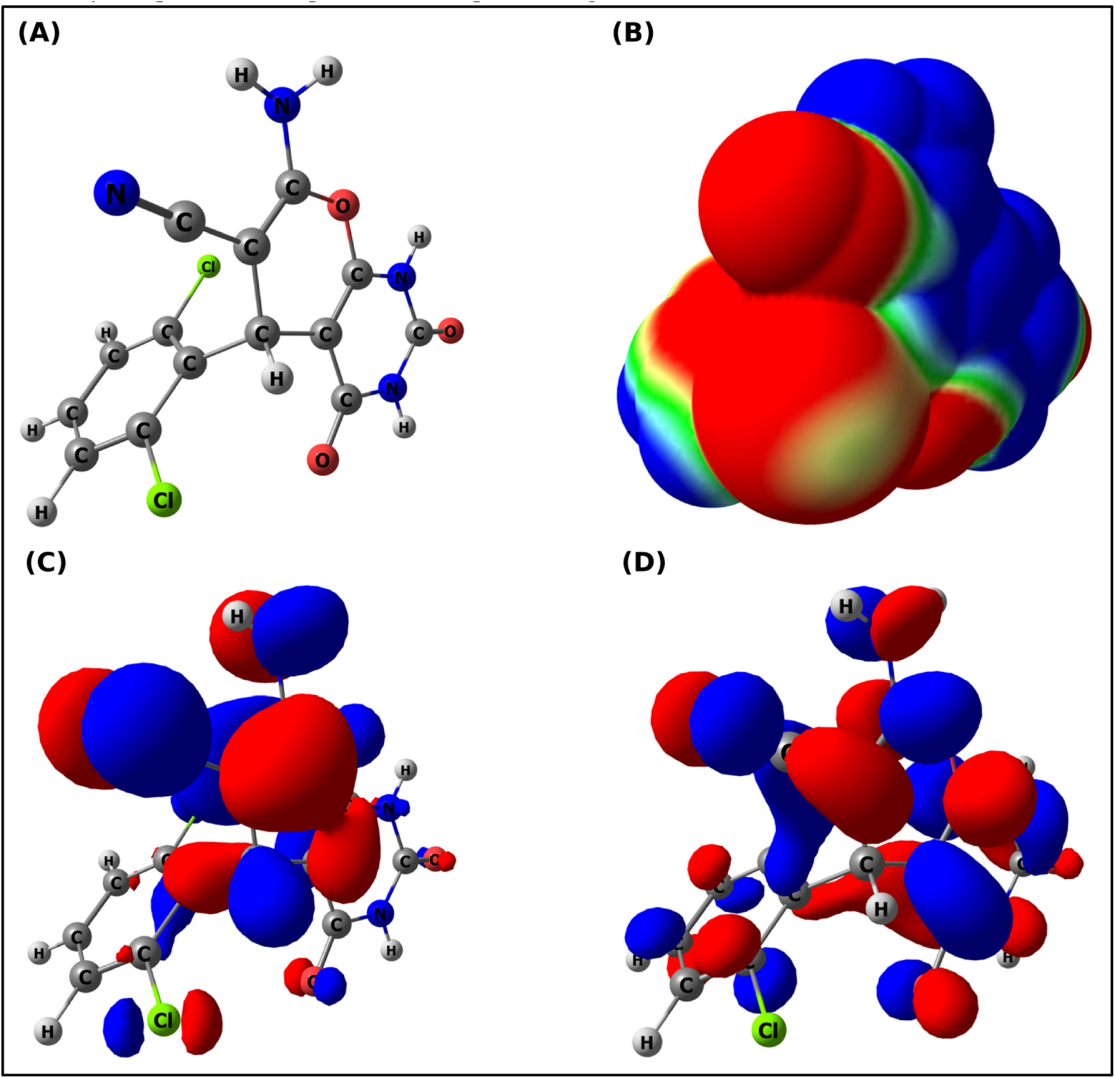
DFT-based molecular visualizations of compound 2h at the B3LYP/def2-TZVP level: (A) optimized geometry, (B) molecular electrostatic potential (MEP) surface, (C) HOMO distribution, and (D) LUMO distribution.

To further understand the electronic behaviour of the synthesised compounds, we calculated and analysed global reactivity parameters using frontier molecular orbitals (FMO) based on the HOMO/LUMO energy levels. FMO calculations provided a clear view of the electronic properties (Table 3) and reactivity of the synthesised structures (2a–2h). The HOMO energy level of each derivative was calculated to vary by approximately 1 eV, indicating a significant variation in the ability of these molecules to donate electrons. Derivatives with lower absolute values of HOMO energy, such as 2b (−6.05 eV) and 2f (−6.41 eV), are expected to be more likely to donate an electron and, therefore, participate in π–π stacking or hydrogen bonding with a potential biological target. Conversely, derivatives with less negative HOMO energies (*e.g.*2c (−6.87 eV) and 2g (−6.58 eV)) indicate greater intrinsic stability of the HOMO but possibly less nucleophilic character. A comparison of the LUMO energies of 2b (−1.57 eV) and 2f (−1.60 eV) suggests a decreased ability to accept electrons. Examination of the entire series indicated a relatively consistent range for the HOMO–LUMO gap (Δ*E*) of approximately 4.5–4.9 eV, indicating a balance between the kinetic stability and reactivity of the compounds. Interestingly compound 2h had an energy gap of Δ*E* = 4.75 eV, which was very close to that of afatinib (Δ*E* = 3.91 eV). This suggests that 2h has favourable electronic characteristics for interaction with biological molecules. It is especially important to note the similarity in electronic properties, as 2h showed the highest cytotoxic potency *in vitro* of all the compounds tested, with an IC_50_ Value of 39.29 µM on the A549 cell line. Thus, there is a coherent relationship between the computed reactivity parameters of 2h and its experimentally observed bioactivity. Representative plots of the optimised geometric structures, MEPs, HOMO, and LUMO surface plots for 2h are provided in Fig. 6A–D, and additional information regarding the MEPs, HOMO, and LUMO surface plots for all derivatives (2a–2h) can be found in the SI (Fig. S33).

**Table 3:** Calculated quantum chemical parameters of the synthesised compounds (2a–2h) and afatinib, computed at the B3LYP/def2-TZVP level of theory^a^

Code	*E*_LUMO_ (eV)	*E*_HOMO_ (eV)	Δ*E*	(*I*)	(*A)*	(*n*)	(*z*)	(*χ*)	(*µ*)	(*ω*)	*µ* (D)
2a	−1.65	−6.53	4.88	6.53	1.65	2.44	0.20	4.09	−4.09	3.43	5.12
2b	−1.57	−6.05	4.48	6.05	1.57	2.24	0.22	3.81	−3.81	3.24	3.89
2c	−2.07	−6.87	4.80	6.87	2.07	2.40	0.21	4.47	−4.47	4.16	8.77
2d	−1.77	−6.57	4.80	6.57	1.77	2.40	0.21	4.17	−4.17	3.62	5.95
2e	−1.79	−6.55	4.76	6.55	1.79	2.38	0.21	4.17	−4.17	3.65	6.17
2f	−1.60	−6.41	4.81	6.41	1.60	2.41	0.21	4.01	−4.01	3.33	4.98
2g	−1.73	−6.58	4.85	6.58	1.73	2.43	0.21	4.16	−4.16	3.56	5.84
2h	−1.68	−6.43	4.75	6.43	1.68	2.38	0.21	4.06	−4.06	3.46	4.35
Afatinib	−1.81	−5.72	3.91	5.72	1.81	1.96	0.26	3.77	−3.77	3.63	3.76

a *E*_LUMO_ = energy of LUMO, *E*_HOMO_ = energy of HOMO, Δ*E* = |*E*_HOMO_–E_LUMO_|, *I* = ionisation potential, *A* = electron affinity, *n* = chemical hardness, *z* = chemical softness, *χ* = electronegativity, *µ* = chemical potential, *ω* = electrophilicity, *µ* = dipole moment.

The chemical potential (*µ*), which reflects the tendency of electrons to escape, was consistently negative (−3.81 to −4.47 eV) across the series. The most negative values (2c, −4.47 eV; 2g, −4.16 eV) suggest a lower tendency to lose electron density, which stabilises these molecules against spontaneous charge transfer. In contrast, 2b (−3.81 eV) and afatinib (−3.77 eV) showed relatively higher *µ* values, indicating a greater propensity to engage in donor–acceptor interactions within the protein-binding sites. The electrophilicity index (*ω*) of the scaffolds (2a–2h) ranged from 3.24–4.16 eV. Compounds 2c (4.16 eV) and 2e (3.65 eV) showed the highest electrophilic character, making them favourable candidates for electron-acceptor roles in protein-ligand binding. Compound 2h (*ω* = 3.46 eV) displayed electrophilicity comparable to that of afatinib (3.63 eV), suggesting a balanced profile of reactivity and selectivity. The results of dipole moment analysis further support these findings. The calculated dipole moments ranged from 3.89 D (2b) to 8.77 D (2c), reflecting the variations in the overall molecular polarity. Highly polar molecules such as 2c (8.77 D) and 2e (6.17 D) are assumed to have stronger solubility in aqueous environments and enhanced orientation within the polar regions of the active site of PDB ID: 4ZXT. In contrast, derivatives with lower dipole moments, including 2b (3.89 D) and 2h (4.35 D), may favour hydrophobic pockets and balanced solubility, contributing to favourable bioavailability. Afatinib exhibited the lowest dipole moment (3.76 D) among the scaffolds (2a–2h), suggesting that moderate polarity, rather than excessive polarity, may be optimal for achieving strong and specific binding interactions.

Therefore, the general trend throughout the series indicates that all electron-withdrawing substituents (CN, Halogen in compounds 2c, 2d, 2e, 2g, and 2h) increased the electrophilicity and dipole moment of the compound, while electron-donating substituents (OCH_3_ in 2b and OH in 2f) raised the HOMO energy levels and decreased the dipole moment, thereby lowering the nucleophilicity. Compound 2h represents an intermediate with a moderately lowered HOMO energy level (−6.43 eV), low LUMO energy (−1.68 eV), appropriate chemical potential (−4.06 eV), reasonable electrophilic index (3.46 eV), and moderate dipole moment (4.35 D). The above characteristics explain the good IC_50_ value (39.29 µM) against the A549 cell line, which clearly shows a strong relationship between the predicted results from the computer models and the measured activity in the cell lines.

#### 2.2.2 Molecular electrostatic potential (MEP) analysis

Building on the orbital-based insights from the FMO calculations, we examined the molecular electrostatic potential (MEP) surfaces to visualise the charge distribution and identify the reactive regions within the scaffolds. The MEP surface provides valuable information regarding the charge distribution and potential reactivity sites within the molecules. Compound 2h (Fig. 6B) showed a red surface around the carbonyl oxygen and cyano nitrogen atoms. The red region denotes a high electron density. Consequently, they can act as hydrogen bond acceptors and interact with the electrophilic region (hydrogen bond donor) of the active sites in 4ZXT. In contrast, regions of electron deficiency (blue) were concentrated over the amino hydrogen atoms, marking them as probable donors of hydrogen bonds with biological targets. The intermediate green regions correspond to areas of near-neutral potential, reflecting relatively nonpolar surfaces.

**Fig. 7:**
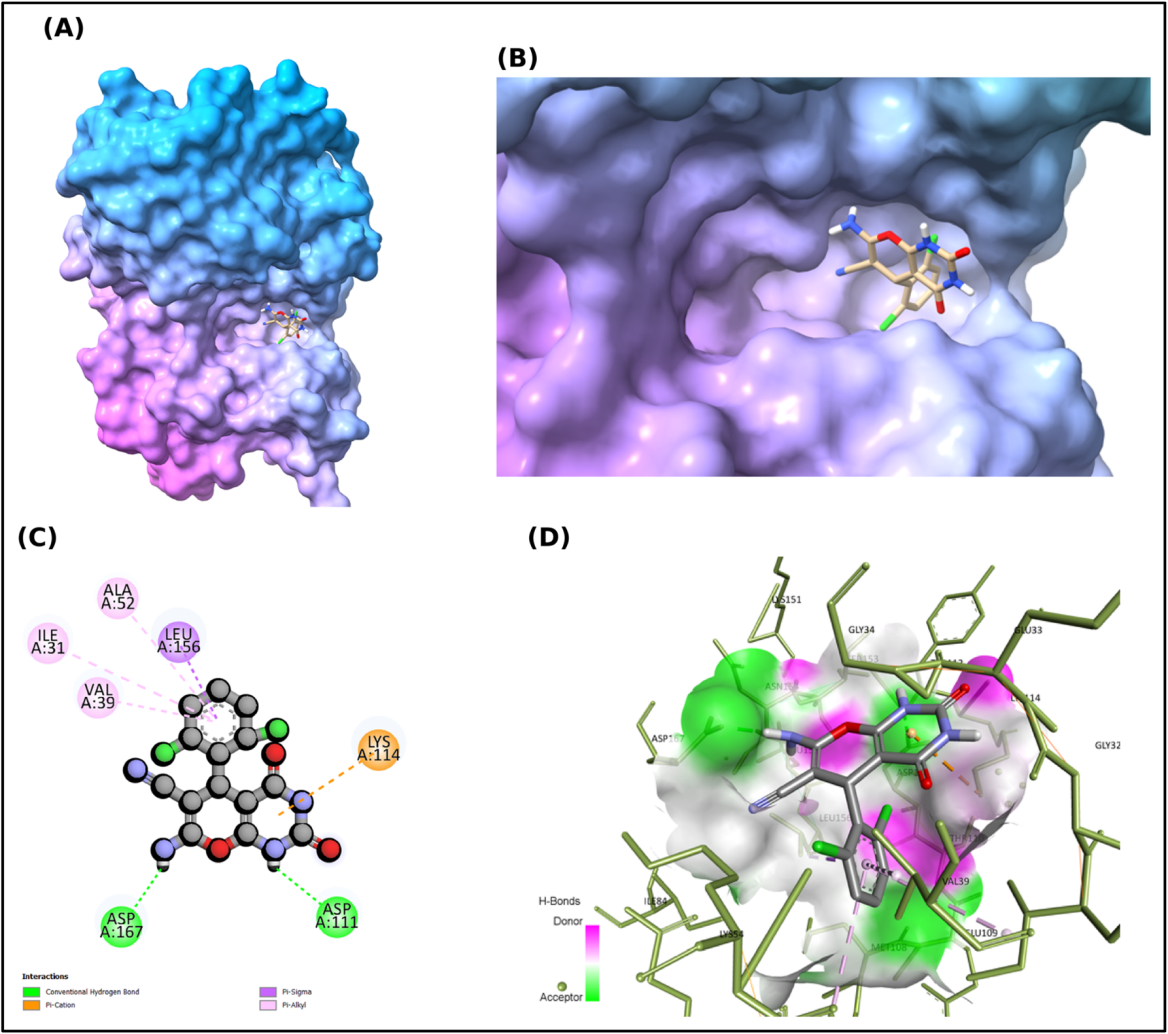
Molecular docking analysis of compound 2h within the active site of the protein (PDB ID: 4ZXT). (A) Surface representation of protein showing the ligand accommodated in the active site. (B) Enlarged binding pocket view highlighting the snug fit of 2h. (C) 2D interaction diagram depicting hydrogen bonding of the amide/amine protons with ASP111 and ASP167, along with π–cation interaction with LYS114 and hydrophobic contacts with VAL39, LEU156, and ALA52. (D) 3D interaction profile illustrating donor/acceptor regions (green/magenta) and hydrophobic surfaces stabilising the ligand orientation.

The MEP results indicate that 2h may interact *via* two different mechanisms: first, by forming a hydrogen bonding interaction between a polar hydrogen atom from 2h and an acceptor group on the protein; second, by forming a non-polar interaction (π–σ or π–alkyl) between 2h and the protein. The MEP results suggested that this increased flexibility is beneficial for protein binding. Furthermore, the calculated electrophilicity index (*ω* = 3.46 eV) for 2h was similar to that of afatinib, suggesting a favourable binding profile. Importantly, the results shown in the MEP maps were consistent with the docking of 2h into 4ZXT (Fig. 7D). In this complex, the electronegative regions of 2h were aligned with the donor groups of the protein, and the electropositive sites of 2h complement the acceptor sites (ASP167 and ASP111) on the enzyme. These MEP-derived predictions were validated using the different types of interactions present in the docking complex. MEP plots and the optimised geometries along with the HOMO–LUMO for each of the synthesised compounds (2a–2h) are provided in the SI (Fig. S33)

#### 2.2.3 Natural bond orbital (NBO) analysis

While MEP highlights the spatial distribution of electron density, a more quantitative understanding of donor–acceptor interactions was obtained through natural bond orbital (NBO) analysis, which revealed intramolecular charge transfer and stabilisation of orbital delocalizations. Second-order perturbation analysis of the Fock matrix (Table 4) in the NBO basis showed pronounced lone-pair → antibonding delocalizations that polarise the amide/amine segment of 2h and rationalise the two experimentally observed donor H-bonds in the ERK2 pocket. The two strongest stabilising interactions were the *n* → π* (donation) interactions of LP(N18) → π*(C17–O21) ≈ 63.7 kcal mol^−1^, LP(N16) → π*(C17–O21) ≈ 53.1 kcal mol^−1^, and LP(N18) → π*(C19–O20) ≈ 51.5 kcal mol^−1^. The high degree of stabilisation by these *n* → π* (donor) interactions is due to the fact that they remove significant amounts of electron density from the adjacent nitrogen-hydrogen bonds, thus significantly increasing the polarity of said nitrogen–hydrogen bonds (thus increasing the acidity of those bonds) which in turn creates an environment where the amide nitrogen–hydrogen bond (N16–H) and the terminal amine nitrogen–hydrogen bond (N18–H) will act efficiently as hydrogen-bond donors. Docking of the protein with the ligand showed that the Asp111 residue accepted a typical hydrogen bond from the amide nitrogen–hydrogen bond, while the Asp167 residue accepted a typical hydrogen bond from the amine nitrogen–hydrogen bond.

**Table 4:** The second-order perturbation analysis of the Fock matrix in the NBO basis for compound 2h (Δ*E* > 15.0 kcal mol^−1^) revealed notable donor–acceptor delocalisation interactions, which contributed significantly to the overall electronic stabilisation of the system

Donor	Acceptor	*E*(2) kcal mol^−1^)^a^	Δ*E* (a.u.)^b^	*F*(*i*, *j*) (a.u.)^c^
LP (N18)	π* (C17–O21)	63.71	0.27	0.118
LP (N16)	π* (C17–O21)	53.12	0.29	0.111
LP (N18)	π* (C19–O20)	51.48	0.29	0.109
LP (N16)	π* (C13–C14)	46.74	0.30	0.106
LP (N1)	π* (C2–C3)	44.49	0.31	0.104
LP (O15)	π* (C13–C14)	33.18	0.38	0.100
LP (O21)	σ* (N16–C17)	30.88	0.63	0.124
LP (O20)	σ* (N18–C19)	30.75	0.63	0.124
LP (O15)	π* (C2–C3)	30.29	0.39	0.096
LP (O21)	σ* (C17–N18)	28.68	0.66	0.123
π (C13–C14)	π* (C19–O20)	25.11	0.32	0.079
π (C2–C3)	π* (C4–N5)	23.52	0.41	0.088
π (C8–C9)	π* (C7–C12)	22.61	0.28	0.071
LP (O20)	σ* (C13–C19)	22.08	0.68	0.110
π (C10–C11)	π* (C7–C12)	21.81	0.27	0.068
π (C10–C11)	π* (C8–C9)	21.27	0.27	0.068
π (C8–C9)	π* (C10–C11)	19.93	0.29	0.068
π (C7–C12)	π* (C10–C11)	19.03	0.30	0.067
π (C7–C12)	π* (C8–C9)	18.72	0.29	0.065
LP (Cl25)	π* (C8–C9)	16.39	0.32	0.065
LP (Cl29)	π* (C7–C12)	15.96	0.33	0.065

a *E*_2_ is the energy of the hyperconjugative interactions.

b Energy difference between the donor and acceptor *i* and *j* NBO orbitals.

c *F*(*i*, *j*) is the Fock matrix element between *i* and *j*. NBO orbitals.

Complementary π → π* delocalizations across the conjugated framework *e.g.*, π(C_13_–C_14_) → π*(C_19_–O_20_) (∼25.1 kcal mol^−1^), π(C_2_–C_3_) → π*(C_4_–N_5_) (∼23.5 kcal mol^−1^), and π(C8–C9) → π*(C_7_–C_12_) (∼22.6 kcal mol^−1^) extend electron density over the aryl/pyrimidinone surface, explaining the π–σ/π–alkyl contacts with Leu156, Val39, Ala52, and Ile31 observed in the interaction map. Moderate σ → σ* hyperconjugations around C_17_–O_21_/N_16_–C_17_ (*e.g.* σ(C_17_–O_21_) → σ*(N_16_–C_17_) ∼28.7 kcal mol^−1^) further tune the bond polarisation and help maintain the partially planar conjugated geometry observed in the optimised structure.

This orbital-level rationale was also aligned with the MEP map (positive potential over N–H hydrogens and negative potential over C=O and cyano regions), providing a coherent electronic basis for the observed binding mode of 2h.

### 2.3 ADME analysis

Following the evaluation of the electronic structure and reactivity descriptors (FMO, MEP, and NBO), the pharmacokinetic profiles and drug-likeness of the synthesised scaffolds were assessed.

The drug-likeness and pharmacokinetic profiles of the synthesised pyrano[2,3-*d*]pyrimidinone derivatives (2a–2h) were assessed using SwissADME and MolSoft web servers (Table 5). All compounds complied with Lipinski's rule of five, with no violations, and had molecular weights between 282–361 g mol^−1^, well below that of afatinib (487.9 g mol^−1^), indicating favourable oral bioavailability. The predicted bioavailability scores were consistently 0.55, which is consistent with the drug-like behaviour. The topological polar surface area (TPSA) values ranged from 124 to 149 Å^2^, balancing solubility and permeability. Compounds 2a, 2d, and 2f, with TPSA values of approximately 124 Å^2^, were well-suited for membrane transport, whereas 2c (148.6 Å^2^) showed the highest polarity and correspondingly lower gastrointestinal (GI) absorption. All other derivatives were predicted to have high GI absorption, and none was BBB-permeable, which is advantageous for anticancer therapy because it limits CNS side effects. Lipophilicity (log *P* = 1.1–1.5) suggests moderate hydrophobicity, which is favourable for passive diffusion, while solubility (log *S* = −2.28 to −3.52) is acceptable and higher than that of afatinib (−4.29). The predicted skin permeability (log *K*_p_ = −7.1 to −7.9 cm s^−1^) indicated a uniform pharmacokinetic behaviour.

**Table 5:** Physicochemical properties and pharmacokinetic descriptors of the synthesised scaffolds (2a–2h) and the standard drug afatinib^a^

Code	M.Wt	RB	HBA	HBD	TPSA (Å^2^)	log po/wi log *P*	log *S* (ESOL)	GI	BBB	log *K*_p_ cm s^−1^	Violations	BAS	DLS
2a	282.25	1	4	3	124.76	1.13	−2.34	High	No	−7.58	0	0.55	−0.62
2b	312.28	2	5	3	133.99	1.40	−2.40	High	No	−7.79	0	0.55	−0.43
2c	307.26	1	5	3	148.55	1.35	−2.28	Low	No	−7.93	0	0.55	−0.67
2d	316.70	1	4	3	124.76	1.40	−2.93	High	No	−7.34	0	0.55	−0.20
2e	361.15	1	4	3	124.76	1.50	−3.24	High	No	−7.57	0	0.55	−0.59
2f	296.28	1	4	3	124.76	1.39	−2.63	High	No	−7.41	0	0.55	−0.53
2g	300.24	1	5	3	124.76	1.22	−2.49	High	No	−7.62	0	0.55	−0.39
2h	351.14	1	4	3	124.76	1.47	−3.52	High	No	−7.11	0	0.55	−0.58
Afatinib	487.95	9	6	3	87.22	3.95	−4.29	High	No	−7.30	0	0.55	1.12

a Parameter definitions: M.Wt, molecular weight; RB, rotatable bonds; HBA, number of hydrogen bond acceptors; HBD-number, hydrogen bond donors; TPSA – topological polar surface area; log *P*, octanol–water partition coefficient; log *S*, solubility (ESOL); GI – gastrointestinal absorption; BBB, blood–brain barrier permeability; log *K*_p_, skin permeability; BAS, bioavailability score; DLS, drug-likeness score.

Among the series, compound 2h stood out, combining a favourable molecular weight, optimal log *P* (1.47), balanced polarity (TPSA = 124.8 Å^2^), high GI absorption, and good solubility, making it a promising lead candidate.

### 2.4 Molecular docking analysis

Although ADME Profiling has demonstrated that the pharmacokinetics of the synthesised scaffolds are acceptable, the therapeutic relevance of these synthesised scaffolds will depend on the degree to which they can interact with the biological target. Molecular docking was used to predict the binding affinity and types of interactions of the synthesised scaffolds (2a–2h) within the active site of the protein (PDB ID: 4ZXT). The results of the docking studies, listed in Table 6, indicated that the binding affinities ranged from −6.69 to −7.58 kcal mol^−1^, whereas the reference drug afatinib exhibited a stronger affinity (−8.01 kcal mol^−1^). Among the synthesised compounds, 2g (−7.58 kcal mol^−1^) and 2h (−7.34 kcal mol^−1^) were identified as the two most potent scaffolds with binding affinities comparable to that of afatinib. The binding mode of compound 2h (Fig. 7) demonstrated the defined positioning of the ligand within the active site of 4ZXT.

**Table 6:** Predicted binding affinities (kcal mol^−1^) of synthesised pyrano[2,3-*d*]pyrimidinone derivatives (2a–2h) and the reference drug afatinib with the (PDB ID: 4ZXT) obtained from AutoDock Vina docking studies

Ligand	2a	2b	2c	2d	2e	2f	2g	2h	Afatinib^a^
Binding affinity (kcal mol^−1^)	−7.20	−7.24	−6.69	−6.91	−6.83	−6.83	−7.58	−7.34	−8.01

a The IC_50_ value for afatinib was adapted from Yuanbiao Tu *et al.* Discovery of novel quinazoline derivatives bearing semicarbazones. *Computational and Structural Biotechnology Journal***16** (2018) 462–478.

The key stabilising interactions between compound 2h and 4ZXT included conventional hydrogen bonds between the amide/amine hydrogens of compound 2h and ASP111 and ASP167, consistent with the electrostatic predictions from the MEP Analysis. Furthermore, the π–cation interactions of compound 2h with LYS114 and the π–sigma/π–alkyl interactions of compound 2h with VAL39, LEU156, and ALA52 also contributed to ligand anchoring. Together, these hydrogen bonding and hydrophobic interactions suggest a dual stabilisation mechanism, consistent with kinase-ligand binding models.

Moreover, NBO analysis of 2h predicted strong donor–acceptor delocalisation between the Amide N–H and heteroaryl π-systems, directly correlating with the observed hydrogen bonding to acidic residues (ASP111/ASP167). Overall, the docking results indicated that 2g and 2h exhibited balanced hydrophilic and hydrophobic interactions in the active site of the protein. These findings provide a robust foundation for subsequent MD simulations to probe dynamic stability and MTT cytotoxic assays to confirm the cellular activity. However, it is important to note that although afatinib is used as a reference standard in MTT assays, it primarily targets EGFR and is considered a negative/organic control for ERK2 kinase domain PDB ID: 4ZXT docking. The steric clashes and donor–donor penalties in the binding site support its off-target profile, indicating that the favourable binding observed for 2g and 2h is more representative of active site engagement.

### 2.5 Molecular dynamics (MD) simulation

**Fig. 8:**
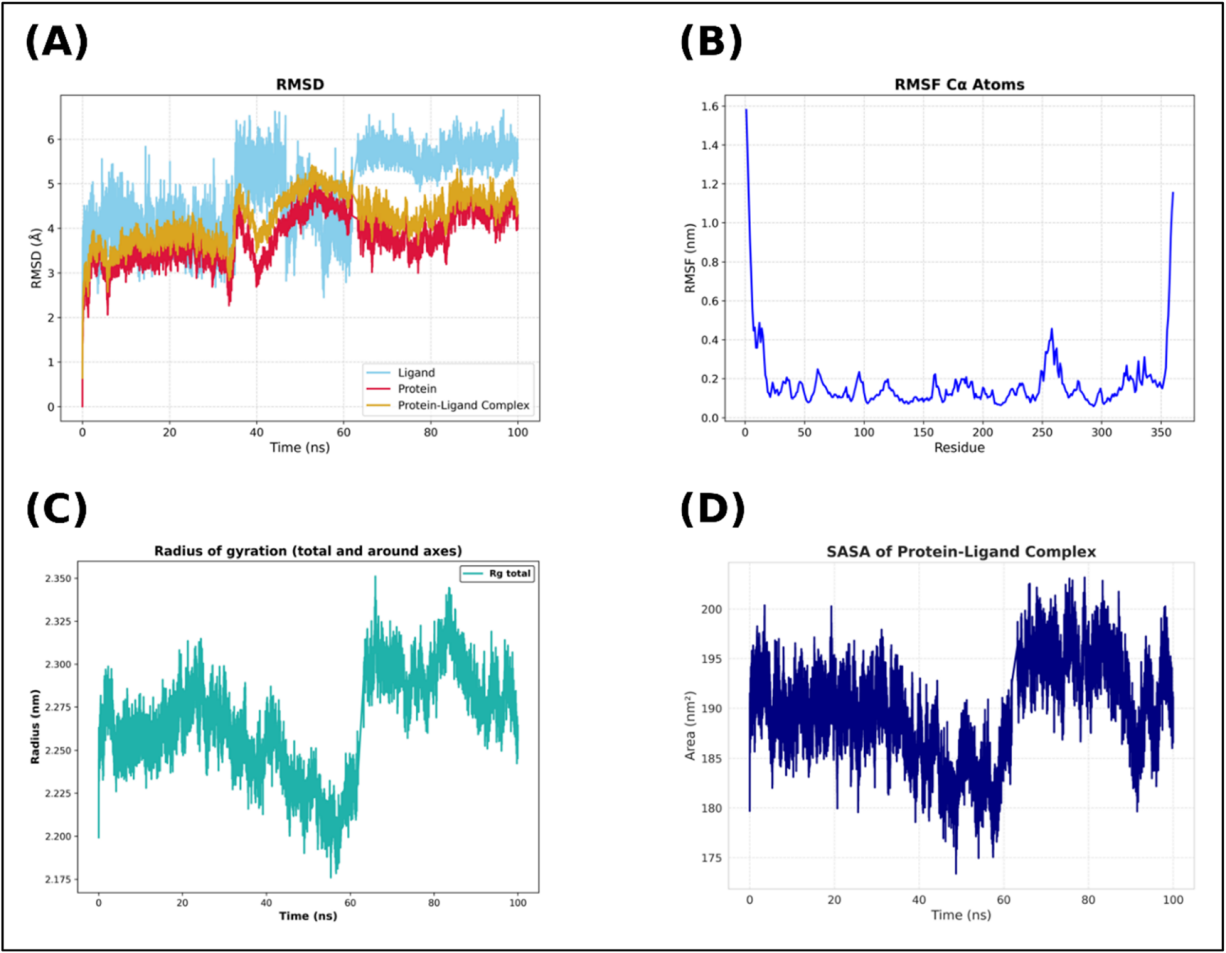
Molecular dynamics (MD) simulation analysis of the 2h-4ZXT complex over 100 ns. (A) RMSD profiles of the ligand, protein backbone, and protein–ligand complex. (B) RMSF of Cα atoms, showing residue-wise flexibility. (C) Solvent accessible surface area (SASA) trajectory of the protein–ligand complex. (D) Radius of gyration (*R*_g_), representing compactness of the protein structure.

Because docking can provide a very rigid view of how compounds bind, we further evaluated the 2h – 4ZXT complex using molecular dynamics (MD) simulations to determine whether the 2h – 4ZXT complex remains stable in a physiologically relevant environment. MD simulations were used in conjunction with docking analysis to assess the stability of the 2h – 4ZXT complex. Fig. 8A shows the RMSD traces for the 2h – 4ZXT complex. The RMSD traces showed that by the end of the first 10–15 ns of the simulation time, the protein backbone was stabilised within the 2.5–3.0 Å range, indicating a stable protein structure in the presence of 2h under physiological conditions. Furthermore, the ligand RMSD trace was very similar to that of the complex, indicating that during the course of the simulation, 2h did not undergo significant rotation away from the binding pocket or dissociate from the binding pocket.

The RMSF traces (Fig. 8B) provide information about localised structural flexibility on a per-residue basis. Consistent with our expectations, the loop and terminal regions showed moderate levels of motion. However, the residues near the active site of the protein showed minimal movement, demonstrating that binding for 2h had a stabilising effect on the active site of the protein. SASA traces (Fig. 8C) fluctuated between 185–200 nm^2^, suggesting that no large-scale unfolding of the protein occurred. Additionally, the radius of gyration (Fig. 8D) remained at approximately 2.25–2.30 nm, indicating that the protein remained folded around compound 2h. These data collectively demonstrate that compound 2h not only favourably interacts with 4ZXT but also maintains the stability of the 2h – 4ZXT complex over long periods of simulation. Therefore, these studies, when combined with docking and DFT-based descriptors, demonstrated that compound 2h is a chemically robust lead compound that should be assessed for its free binding energy (MM/GBSA) and subsequently validated biologically.

#### 2.5.1 Protein–ligand interaction energy

**Fig. 9:**
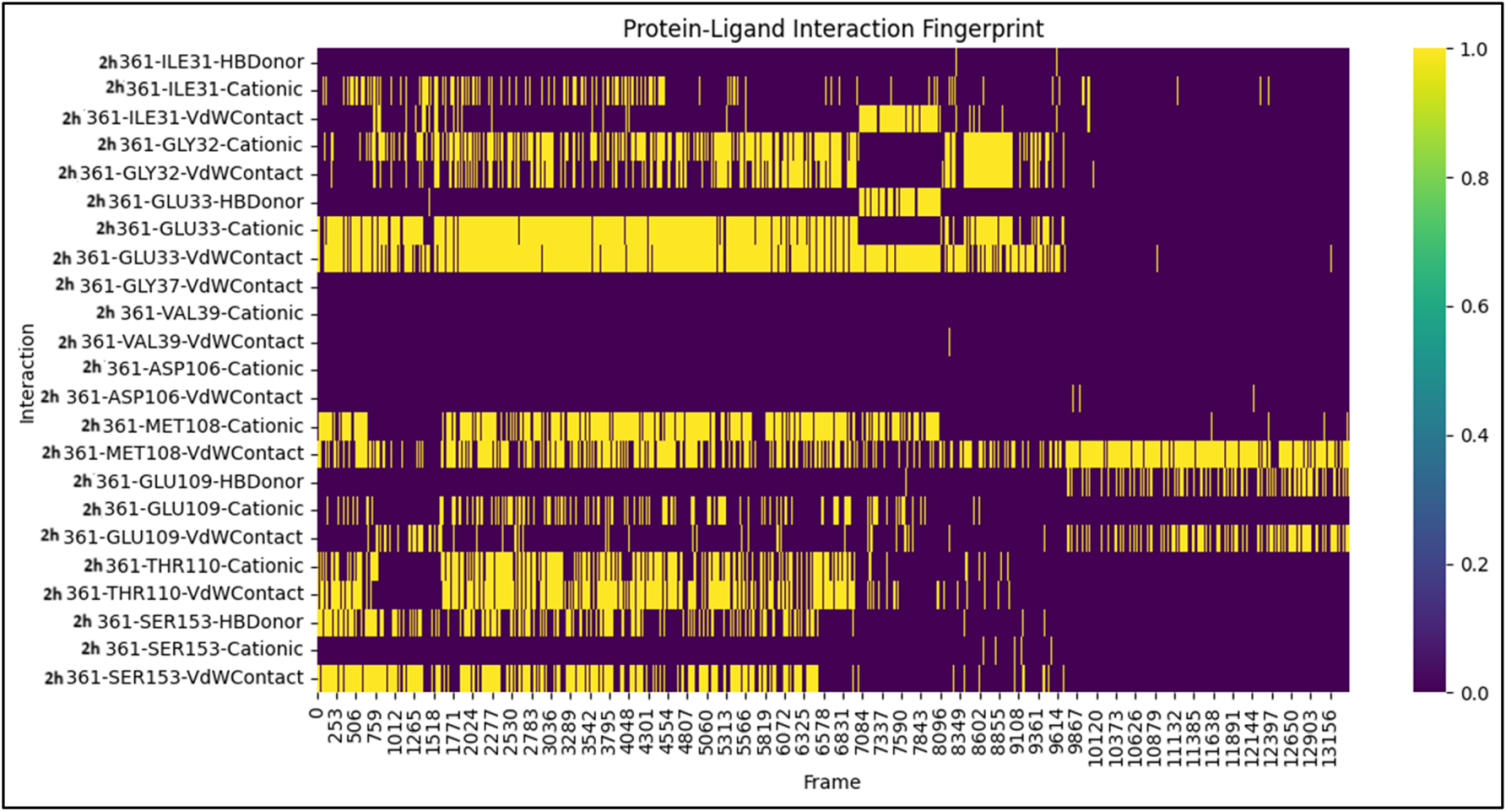
Protein–ligand interaction fingerprint (PLIF) plot for the 2h-4ZXT complex over a 100 ns MD simulation. The persistence and type of non-covalent interactions (hydrogen bonding, van der Waals contacts, and cationic interactions) between compound 2h and key residues within the 4ZXT active site are represented across simulation frames.

In addition to using global stability measures (such as RMSD, RMSF, Rg, and SASA), evaluating the molecular contacts involved in maintaining the structural integrity of a complex is essential. Therefore, Protein–Ligand Interaction Fingerprint (PLIF) analysis was performed on the 2h-4ZXT complex. PLIF analysis provided a time-dependent picture of the molecular contacts that support the structural integrity of the 2h-4ZXT complex along the 100 ns trajectory (Fig. 9). Compound 2h exhibited an extensive array of interactions, demonstrating its flexibility to fit into the binding site and its capacity to engage in multiple types of interactions that provide structural stabilisation. Hydrogen-bonding interactions between 2h and GLU33, GLU109, and SER153 were present in a high percentage of the simulated structures, indicating that hydrogen-bonding interactions significantly contribute to the anchoring of the ligand within the ATP-binding cleft. van der Waals and cationic interactions were commonly observed between compound 2h and ILE31, GLY32, VAL39, and MET108, demonstrating that hydrophobic residues play a supporting role in stabilising the binding interaction. Additionally, compound 2h interacted with ASP106, which has been shown to be important in regulating enzymatic activity, thus highlighting the functional relevance of 2h binding.

It is also important to note that the persistence of these interactions at various points during the simulation indicated that compound 2h did not experience significant displacement during the course of the simulation, which is consistent with the RMSD and *R*_g_ data. Overall, the PLIF data indicate that compound 2h forms a dynamic balance between hydrogen bonding and hydrophobic contacts, which can account for the favourable docking affinity and MD-derived stability of compound 2h.

#### 2.5.2 MM/GBSA binding energy, PCA and FEL analysis

**Fig. 10:**
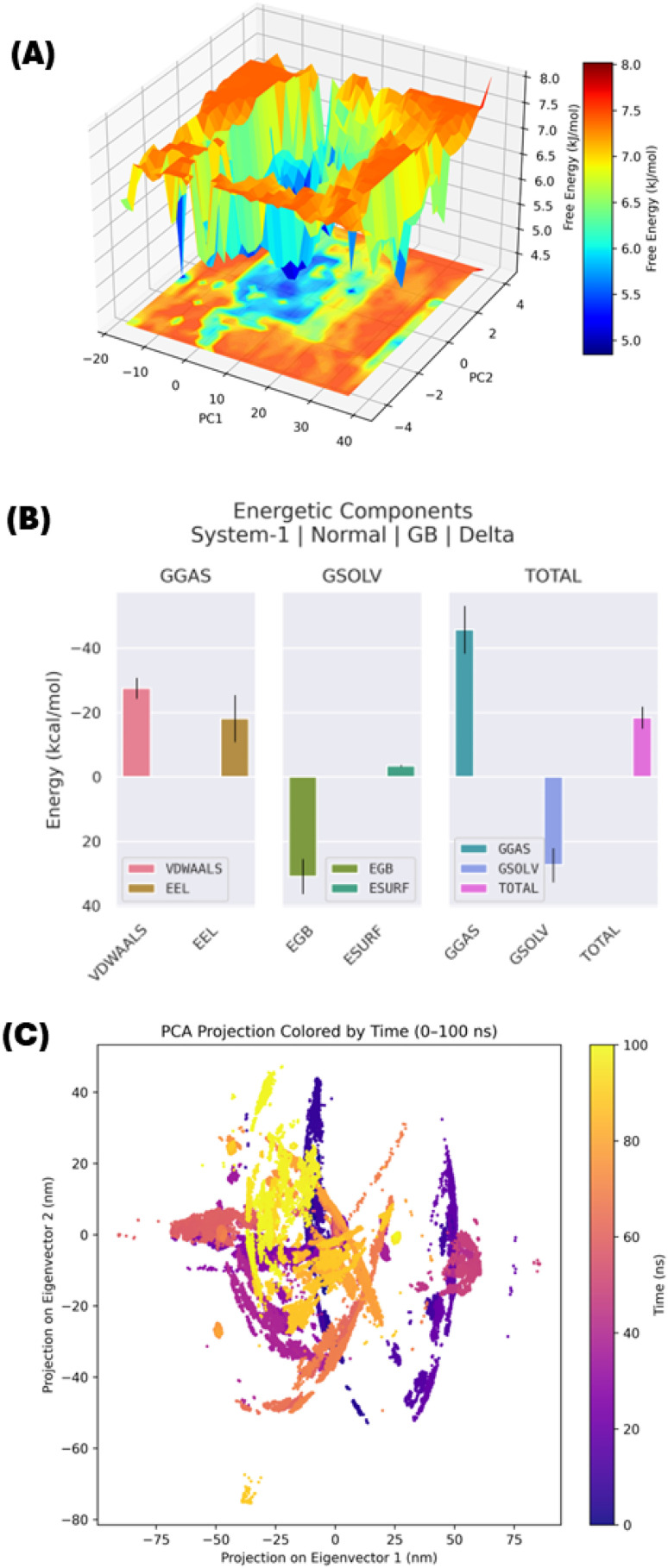
Integrated binding free energy and conformational dynamics analysis of the 4ZXT-2h complex. (A) MM/GBSA energy decomposition showing contributions from van der Waals, electrostatic, polar solvation, and non-polar solvation terms, confirming favourable binding energetics. (B) Free energy landscape (FEL) plotted along the first two principal components, highlighting deep and stable energy minima that indicate conformational stabilization of the complex. (C) Principal component analysis (PCA) projection coloured by simulation time (0–100 ns), illustrating the conformational sampling and clustering of stable states during the trajectory.

In addition to analysing RMSD, RMSF, Rg, SASA, and PLIF, we performed an additional quantitative analysis of the energy contributions and conformational space explored to confirm our initial findings. Therefore, we used MM/GBSA to compute the binding free energy of 2h to 4ZXT, as well as Principal Component Analysis (PCA) and Free Energy Landscape (FEL) analyses to identify the major motions and energy basins present in the complex (Fig. 10). From the MM/GBSA decomposition (Fig. 10B), it is clear that both van der Waals and electrostatic interactions dominate as stabilising forces for the 2h-4ZXT complex. However, the penalty due to solvent effects slightly reduces this contribution. These observations strongly support the idea that the binding pocket of 4ZXT can stably accommodate 2h spontaneously, thereby confirming the predictions made from our molecular docking studies.

Additionally, FEL analysis (Fig. 10A), projected onto the first two principal components, demonstrated low-energy basins of motion for the complex, each separated by moderate barriers, and therefore indicated that the majority of the simulation time (*i.e.* 100 ns) of the complex was spent exploring the most favourable energetics. The fact that the FEL analysis identified specific, well defined minima corresponds to the previously observed stable RMSD and *R*_g_ values, and therefore indicates that the 2h maintains the protein in a very constrained conformational space with little to no unfolding of the protein. This conclusion is further supported by the observation of PCA clustering, which showed that the complex converges into a small conformational subspace over time. In addition, trajectory progression (Fig. 10C) clearly shows that the active site residues of 4ZXT are unable to move freely when bound for 2h, and therefore support the conclusions drawn from the SASA fluctuations. Overall, these additional analyses demonstrate that not only does compound 2h binds to 4ZXT with energetically favourable binding properties and holds the kinase in a restrained state of motion, which is a highly desirable property for selective inhibition.

### 2.6 *In vitro* cytotoxicity studies by MTT assay

Although computational studies have provided strong evidence for the stability, binding energetics, and pharmacokinetic potential of pyrano[2,3-*d*]pyrimidinone derivatives, it is essential to validate these predictions in a biological context. Therefore, the cytotoxic potential of the synthesised scaffolds was evaluated *in vitro* using the A549 lung carcinoma cell line. The cytotoxic effects of the scaffolds (2a–2h) were thoroughly assessed in the A549 human lung carcinoma cell line using theMTT assay. Afatinib served as a positive control in the cytotoxicity tests against A549 cells, enabling direct comparison of the growth-inhibitory effects of the synthesized derivatives within a biologically relevant cellular environment.

The IC_50_ values summarised in Table 7 reveal a clear structure–activity relationship among the derivatives. Compound 2h exhibited the most potent inhibitory effect (IC_50_ = 39.29 µM), followed by 2d (47.91 µM), 2f (58.15 µM), and 2c (59.13 µM), indicating that halogen and pseudohalogen substitutions at the phenyl ring significantly enhanced antiproliferative activity. In contrast, the derivatives 2a (120.65 µM) and 2e (90.17 µM) displayed weaker activities. Afatinib was considered the reference drug and served two complementary roles in this study: as a biological positive control in MTT assays (EGFR-driven cytotoxicity in A549 cells) and as a docking comparator against the crystal structure 4ZXT, which corresponds to the ERK2 kinase domain. In this context, afatinib was used as a negative control to highlight the potential off-target interactions. This dual consideration allowed us to place the newly synthesised derivatives in perspective, comparing their anticancer activity with that of a clinically validated drug while also probing their putative engagement with the ERK2 signalling framework represented by 4ZXT.

**Table 7:** *In vitro* cytotoxic activity (IC_50_, µM) of synthesised scaffolds (2a–2h) against the A549 lung carcinoma cell line, as determined by MTT assay

Code	2a	2b	2c	2d	2e	2f	2g	2h	Afatinib^a^
IC_50_ (µM)	120.65	89.09	59.13	47.91	90.17	58.15	69.97	39.29	1.4

a The IC_50_ value of afatinib was adapted from the study by Tsai *et al.* Afatinib triggers a Ni^2+^-resistant Ca^2+^ influx pathway in A549 non-small cell lung cancer cells. *Fundamental & Clinical Pharmacology* 2022, **37**, 253–262.

**Fig. 11:**
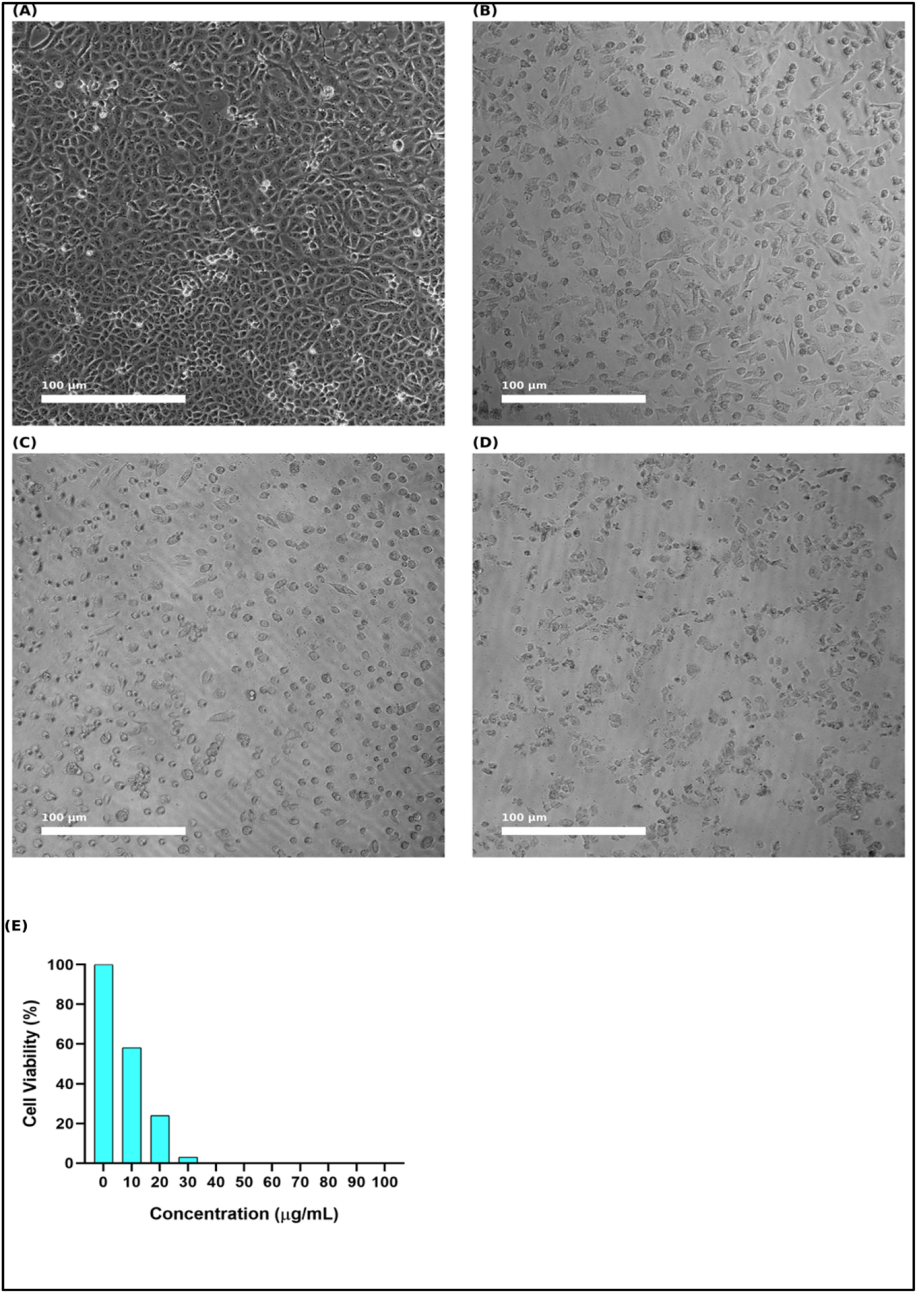
*In vitro* cytotoxic effect of compound 2h against against A549 lung carcinoma cells. (A) Untreated A549 control cells showed a normal morphology and confluency. (B–D) Microscopic images of A549 cells treated with increasing concentrations of compound 2h, showing progressive loss of cell density and morphological distortion. (E) Quantitative representation of cell viability (%).

The reference drug afatinib demonstrated superior potency (IC_50_ = 1.4 µM);^29^ however, compound 2h exhibited a reasonable cytotoxic profile within the micromolar range, highlighting its potential as a lead scaffold for further optimisation. Microscopic imaging of A549 cells treated for 2h (Fig. 11) provided direct morphological evidence of the cytotoxicity. Untreated control cells exhibited a dense, confluent monolayer with intact morphology, whereas the treated cells displayed progressive dose-dependent changes, including cell shrinkage, rounding, detachment, and loss of adherence at higher concentrations. These visual alterations correlated well with the quantitative viability assay, where cell survival sharply declined at concentrations above 20 µg mL^−1^, consistent with the IC_50_ value. Representative cell viability plots and microscopic triplicates for compounds 2a–2h are shown in Fig. S36.

Thus, the MTT assay confirmed that 2h exerted significant antiproliferative effects on A549 cells, with potency aligned with its favourable ADME profile, docking interactions within the active site, and dynamic stability observed in MD simulations. This integrative evidence strengthens the case of 2h as a promising candidate for further preclinical evaluation.

### 2.7 Structure–activity relationship (SAR) analysis

While docking scores provided the first indication of how well ligands would bind, regression analysis of the MTT cytotoxicity data for compounds 2a–2h in Fig. S35 indicated that there was no correlation between docking energy and cytotoxicity, indicating that substituent-dependent structure–activity relationships (SAR) and other pharmacokinetic properties are better indicators of biological responses than docking energy. The combination of theoretical insights from docking, MD simulations, MMGBSA, and free energy landscape calculations with experimental cytotoxicity data supports this view, where compound 2h was identified as the most promising compound based on both docking energy and cytotoxicity.

Compound 2a exhibited very low activity (IC_50_ = 120.65 µM), establishing the importance of incorporating a phenyl ring into the scaffold to improve its bioactivity. Compouns 2b exhibited a moderately higher activity owing to the addition of an electron-donating methoxy group (IC_50_ = 89.09 µm). Addition of an electron-withdrawing cyano group to compound 2c resulted in increased polarity and only a moderate increase in cytotoxicity (IC_50_ = 59.13 µm). Among the halo-substituted analogues, the introduction of a chlorine atom (compound 2d) significantly increased cytotoxicity (IC_50_ = 47.91 µm); however, the introduction of a larger bromine atom (compound 2e) decreased cytotoxicity, which could be attributed to sterically hindered access to the active site of the protein. Compounds 2f (methoxy) and 2g (fluoro) exhibited moderate cytotoxicity (IC_50_ = 58.15 and 65.97 µm, respectively). The high level of cytotoxicity observed for compound 2h (IC = 39.29 µm) can be explained by the optimal hydrophobic packing and steric complementarity afforded by the two chlorine atoms present at the ortho position, validating its identification as the preferred lead scaffold.

## 3 Conclusions

In this study, a green and efficient methodology was developed for the one-pot synthesis of pyrano[2,3-*d*]pyrimidinone derivatives (2a–2h) using silver tungstate (Ag_2_WO_4_) nanoparticles as a heterogeneous catalyst. This approach successfully combined synthetic innovation with computational and biological validation to explore the anticancer potential of the resulting scaffolds in the A549 lung carcinoma cell line. Future studies will extend the cytotoxic evaluation of the synthesized pyrano[2,3-*d*]pyrimidinones to additional cancer cell lines in order to further validate their anticancer potential and broaden the biological relevance of the present findings.

Silver tungstate nanoparticles exhibit a very high catalytic performance and allow for fast and selective transformations at low temperatures and under non-toxic conditions. Compound 2h demonstrated the highest cytotoxic activity among the synthesised compounds, with an IC_50_ of 39.29 µM, which was significantly higher than that of unsubstituted parent scaffold 2a (IC_50_ = 120.65). Complementary *in silico* analyses, including DFT, molecular docking, MD simulations, and ADME screening, provided insights into the structural stability, interactions with biological targets, and pharmacokinetics of the synthesised compounds, thereby revealing the relationship between the predicted theoretical data and experimental results.

This study is the first to report the application of Ag_2_WO_4_ nanoparticles as nanocatalysts for the construction of pyrano[2,3-*d*]pyrimidinone frameworks, establishing a sustainable route for generating bioactive heterocyclic scaffolds with promising anticancer profiles. Although the compounds described herein are foundational scaffolds, further structural modification could improve the selectivity, potency, and drug-like properties to enable the synthesis of clinically viable anticancer drugs derived from the green synthetic platform used in this study.

## 4 Materials and methods

### 4.1 Preparation of Ag_2_WO_4_ catalyst

Stoichiometric aqueous solutions of sodium tungstate dihydrate (Na_2_WO_4_·2H_2_O, 5 mmol) and silver nitrate (AgNO_3_, 10 mmol) were prepared. The silver nitrate solution was slowly introduced into the sodium tungstate solution with continuous stirring until complete precipitation occurred. The mixture was then placed in a 100 mL Teflon-lined stainless-steel autoclave and subjected to hydrothermal processing at 180 °C for 16 h. After natural cooling to room temperature, the precipitate was separated by centrifugation, thoroughly rinsed with deionised water and ethanol, and dried at 60 °C overnight.

### 4.2 General procedure for the synthesis of pyrano[2,3-*d*]pyrimidine-6 carbonitrile

A 100 mL round-bottom flask was loaded with malononitrile (1.1 mmol), aldehyde (1.0 mmol), barbituric acid (1.0 mmol), and Ag_2_WO_4_ (2.5 mol%) in a 1 : 1 ethanol–water mixture. The mixture was stirred at 70 °C for the required duration, and its progress was monitored using thin-layer chromatography (TLC) with ethyl acetate/petroleum ether (3 : 1) as the mobile phase. Once the reaction was complete, the mixture was allowed to cool to room temperature, and the precipitate was separated by vacuum filtration. The crude solid was dissolved in ethanol with a few drops of DMSO to enhance the solubility and filtered again to eliminate any remaining Ag_2_WO_4_ catalyst. The resulting filtrate was poured into cold water to re-precipitate the product, which was collected and further purified by recrystallisation from ethanol to yield pure pyrano[2,3-*d*]pyrimidinone derivatives (2a–2h). The structures of the obtained compounds were characterised using FT-IR, ^1^H NMR, and ^13^C NMR spectroscopy.

### 4.3 Optimization of reaction

**Scheme 1:**
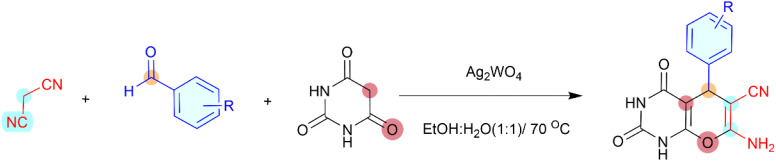
Multicomponent reaction synthesis of pyrano[2,3-*d*]pyrimidinone derivatives (2a–2h).

To identify the most suitable eco-friendly solvent system and catalyst loading, a model multicomponent reaction (Scheme 1) was performed using various green solvents and temperature in the presence of Ag_2_WO_4_. The optimisation results are listed in Table 8.

**Scheme 2:**
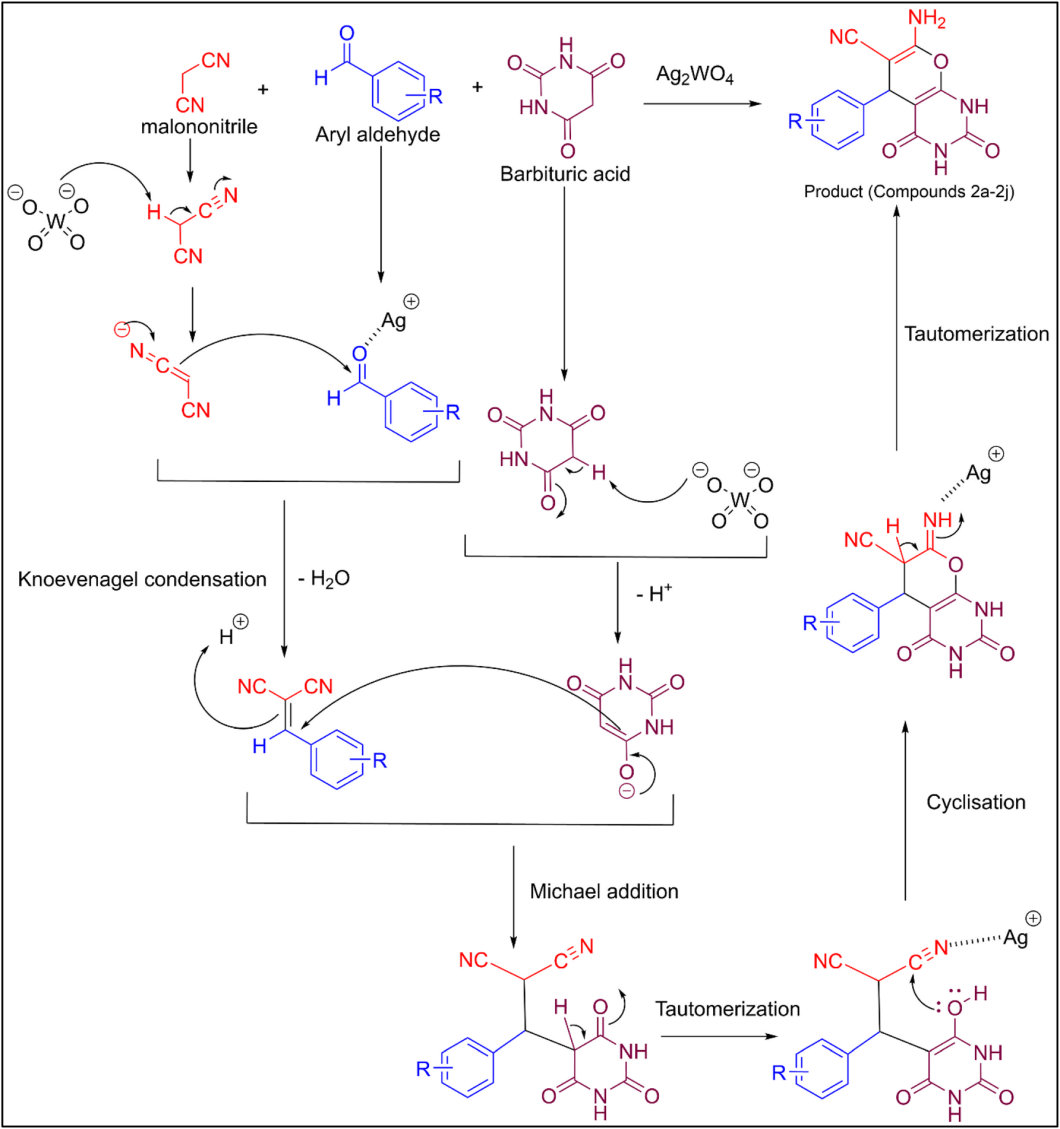
Proposed mechanism for the Ag_2_WO_4_-catalyzed synthesis of pyrano[2,3-*d*]pyrimidinone.

**Table 8:** Optimisation of the reaction with different green solvents and conditions

Entry	Ag_2_WO_4_ (mol%)	Solvent/temperature^a^ (°C)	Time (min)	Yield^b^ (%)
1	2.5	H_2_O/RT	30	71
2	2.5	EtOH/RT	30	75
3	2.5	EtOH–H_2_O (1 : 1)/RT	5–8	80
4	2.5	H_2_O/100	15	78
5	2.5	EtOH/80	10	84
6	2.5	EtOH–H_2_O (1 : 1)/70	5–8	95
7	2.5	PEG-400/RT	30	78
8	2.5	PEG-400/100	10	81
9	5.0	EtOH–H_2_O (1 : 1)/70	5–8	95

a Reaction conditions: barbituric acid (1.0 mmol), benzaldehyde (1.0 mmol), and malononitrile (1.1 mmol) were combined with different solvents and catalyst amounts at different temperatures.

b Isolated yields.

Reactions performed in pure aqueous medium at room temperature afforded only a moderate yield (71% in 30 min), whereas ethanol under identical conditions resulted a marginal improvement (yield = 75%). In contrast, a mixed protic solvent system (EtOH–H_2_O, 1 : 1 v/v) at 70 °C was highly efficient, delivering the target product within 5–8 min in excellent yield (95%). This remarkable enhancement can be attributed to the synergistic hydrogen-bonding environment of the binary solvent, in which the high surface energy of water promotes proton transfer and facilitates rapid product precipitation, thereby accelerating reaction completion. To evaluate the influence of catalyst loading, the amount of Ag_2_WO_4_ was increased from 2.5 mol% to 5.0 mol% in the EtOH–H_2_O (1 : 1) system. However, no improvement in yield was observed (remaining at 95%), confirming that 2.5 mol% Ag_2_WO_4_ was sufficient to efficiently promote the transformation.

Thus, EtOH–H_2_O (1 : 1) at 70 °C with 2.5 mol% Ag_2_WO_4_ was established as the optimal condition, affording the desired products in excellent yields within a few minutes (Table 8). These findings underscore the critical role of binary protic solvents in enhancing the reactivity and highlight the sustainability advantages of this protocol, including minimal catalyst usage, reduced energy demand, and excellent efficiency.

### 4.4 General experimental procedures

The synthesised pyrano[2,3-*d*]pyrimidine scaffolds were characterised using spectroscopic techniques. Infrared spectra were obtained using a PerkinElmer Spectrum Two FTIR spectrometer equipped with an attenuated total reflectance (ATR) accessory that included a diamond crystal and a deuterated triglycine sulfate (DTGS) detector. The instrument was operated within the range of 4000–400 cm^−1^, with four scans per sample and a resolution of 4 cm^−1^. The spectra were collected in ATR mode without any additional sample preparation. ^1^H and ^13^C NMR spectra were recorded in DMSO-d_6_ using a JEOL 600 MHz spectrometer.

### 4.5 Spectral results

#### 4.5.1 7-amino-2,4-dioxo-5-phenyl-1,3,4,5-tetrahydro-2*H*-pyrano[2,3-*d*]pyrimidine-6 carbonitrile (2a, C_14_H_10_N_4_O_3_)

Off-white solid; mp 218–219 °C; ^1^H NMR (600 MHz, DMSO-*d*_6_): *δ* = 12.06 (s, 1H), 11.06 (s, *J* = 1.2 Hz, 1H), 7.32–7.26 (m, 2H), 7.23–7.17 (m, 3H), 7.10, 4.22 (d, *J* = 1.2 Hz, 2H) ppm; ^13^C NMR(151 MHz, DMSO-*d*_6_)): *δ* = 166.28, 161.46, 156.11, 153.34, 147.99, 132.12, 131.12, 130.56, 123.03, 92.32, 62.72, 39.50 ppm; FT-IR (*ν*, cm^−1^): 3404 (Primary N–H), 3190 (Amide N–H), 2198 (C≡N), 1683 & 1634 (C=O), 1094 (C–O–C); HRMS (ESI) *m*/*z* [M + Na]^+^ calcd for C_14_H_10_N_4_O_3_Na^+^:305.0645, found: 305.0641.

#### 4.5.2 7-amino-5-(4-methoxyphenyl)-2,4-dioxo-1,3,4,5-tetrahydro-2*H*-pyrano[2,3-*d*]pyrimidine-6-carbonitrile (2b, C_15_H_12_N_4_O_4_)

Off-white solid; mp 270–271 °C; ^1^H NMR (600 MHz, DMSO-*d*_6_): *δ* = 12.02 (s, 1H), 11.03 (s, 1H), 7.10 (dd, *J* = 14.3 Hz, 10.2 Hz, 6H), 4.16 (s, 1H), 3.72 (s, 3H) ppm; ^13^C NMR(151 MHz, DMSO-*d*_6_)): *δ* = 167.06, 162.68, 162.13, 156.64, 154.11, 140.86, 137.07, 132.97, 123.88, 119.44, 118.24, 93.38, 63.76, 59.64, 39.50 ppm; FT-IR (*ν*, cm^−1^): 3448 (Primary N–H), 3185 (Amide N–H), 2195 (C≡N), 1716 &1674 (C=O), 1096 (C–O–C); HRMS (ESI) *m*/*z* [M + H]^+^ calcd for C_15_H_13_N_4_O_4_^+^:313.0931, found: 313.0933.

#### 4.5.3 7-amino-5-(4-cyanophenyl)-2,4-dioxo-1,3,4,5-tetrahydro-2*H*-pyrano[2,3-*d*]pyrimidine-6-carbonitrile (2c, C_15_H_9_N_5_O_3_)

Off-white solid; mp 250–252 °C; ^1^H NMR (600 MHz, DMSO-*d*_6_): *δ* = 12.12 (s, 1H), 11.08 (s, 1H), 7.75 (dd, *J* = 4.1 Hz, 1.2 Hz, 2H), 7.54–7.33 (m, 2H), 7.21 (s, 2H), 4.33 (d, *J* = 1.2, 1H) ppm; ^13^C NMR(151 MHz, DMSO-*d*_6_)): *δ* = 166.13, 161.40, 156.29, 153.37, 153.17, 135.97, 132.25, 122.56, 122.52, 113.23, 91.10, 61.32, 39.50 ppm; FT-IR (*ν*, cm^−1^): 3373 (Primary N–H), 3184 (Amide N–H), 2200 (C≡N), 1719 &1670 (C=O), 1100 (C–O–C); HRMS (ESI) *m*/*z* [M + H]^+^ calcd for C_15_H_10_N_5_O_3_^+^:308.0778, found: 308.0771.

#### 4.5.4 7-amino-5-(4-chlorophenyl)-2,4-dioxo-1,3,4,5-tetrahydro-2*H*-pyrano[2,3-*d*]pyrimidine-6-carbonitrile (2d, C_14_H_9_ClN_4_O_3_)

Off-white solid; mp 240–242 °C; ^1^H NMR (600 MHz, DMSO-*d*_6_): *δ* = 12.09 (s, 1H), 11.08 (s, 1H), 7.34 (d, *J* = 3.9 Hz, 2H), 7.24 (d, *J* = 4.0 Hz, 2H), 7.16 (s, 2H), 4.24 (s, 1H) ppm; ^13^C NMR(151 MHz, DMSO-*d*_6_)): *δ* = 162.45, 160.14, 157.61, 152.34, 149.49, 143.16, 132.14, 129.73, 129.37, 129.30, 128.19, 119.05, 88.01, 58.34, 35.18 ppm; FT-IR (*ν*, cm^−1^): 3389 (Primary N–H), 3187 (Amide N–H), 2196 (C≡N), 1717 &1673 (C=O), 1096 (C–O–C); HRMS (ESI) *m*/*z* [M + H]^+^ calcd for C_14_H_10_ClN_4_O_3_^+^:317.0436, found: 317.0430.

#### 4.5.5 7-amino-5-(4-bromophenyl)-2,4-dioxo-1,3,4,5-tetrahydro-2*H*-pyrano[2,3-*d*]pyrimidine-6-carbonitrile (2e, C_14_H_9_BrN_4_O_3_)

Grey solid; mp 231–233 °C; ^1^H NMR (600 MHz, DMSO-*d*_6_): *δ* = 12.09 (s, 1H), 11.07 (s, 1H), 7.67–7.38 (m, 2H), 7.26–7.08 (m, 4H), 4.23 (d, *J* = 1.2 Hz, 1H) ppm; ^13^C NMR(151 MHz, DMSO-*d*_6_)): *δ* = 162.42, 157.59, 152.33, 149.46, 143.57, 131.09, 129.67, 119.73, 119.02, 87.93, 58.25, 35.23 ppm; FT-IR (*ν*, cm^−1^): 3384 (Primary N–H), 3184 (Amide N–H), 2195 (C≡N), 1717 &1673 (C=O), 1098 (C–O–C); HRMS (ESI) *m*/*z* [M + H]^+^ calcd for C_14_H_10_BrN_4_O_3_^+^:360.9931, found: 360.9933.

#### 4.5.6 7-amino-2,4-dioxo-5-(*p*-tolyl)-1,3,4,5-tetrahydro-2*H*-pyrano[2,3-*d*]pyrimidine-6-carbonitrile (2f, C_15_H_12_N_4_O_3_)

Off-white solid; mp 224–226 °C; ^1^H NMR (600 MHz, DMSO-*d*_6_): *δ* = 12.04 (s, 1H), 11.04 (d, *J* = 1.4 Hz, 1H), 7.23–6.92 (m, 6H), 4.17 (d, *J* = 1.3 Hz, 1H), 2.25 (d, *J* = 1.4 Hz, 3H) ppm; ^13^C NMR(151 MHz, DMSO-*d*_6_)): *δ* = 162.57, 157.70, 152.29, 149.64, 141.36, 135.92, 128.97, 127.32, 119.36, 88.77, 59.18, 35.42, 20.76 ppm; FT-IR (*ν*, cm^−1^): 3389 (Primary N–H), 3187 (Amide N–H), 2196 (C≡N), 1717 &1673 (C=O), 1096 (C–O–C); HRMS (ESI) *m*/*z* [M + H]^+^ calcd for C_15_H_13_N_4_O_3_^+^:297.0982, found: 297.0985.

#### 4.5.7 7-amino-5-(4-fluorophenyl)-2,4-dioxo-1,3,4,5-tetrahydro-2*H*-pyrano[2,3-*d*]pyrimidine-6-carbonitrile (2g, C_14_H_9_FN_4_O_3_)

Off-white solid; mp 259–261 °C; ^1^H NMR (600 MHz, DMSO-*d*_6_): *δ* = 12.07 (s, 1H), 11.06 (s, 1H), 7.24 (tt, *J* = 2.6 Hz, 1.3 Hz, 2H), 7.11 (dd, *J* = 7.9 Hz, 3.4 Hz, 4H), 4.24 (s, 1H) ppm; ^13^C NMR(151 MHz, DMSO-*d*_6_)): *δ* = 162.45, 160.25, 157.57, 152.24, 149.49, 140.32, 129.68, 128.82, 119.12, 115.31, 114.52, 88.29, 58.65 ppm; FT-IR (*ν*, cm^−1^): 3389 (Primary N–H), 3186 (Amide N–H), 2197 (C≡N), 1721 &1675 (C=O), 1099 (C–O–C); HRMS (ESI) *m*/*z* [M + H]^+^ calcd for C_14_H_10_FN_4_O_3_^+^:301.0731, found: 307.0726.

#### 4.5.8 7-Amino-5-(2,6-dichlorophenyl)-2,4-dioxo-1,3,4,5-tetrahydro-2*H*-pyrano[2,3-*d*]pyrimidine-6-carbonitrile (2h, C_14_H_8_Cl_2_N_4_O_3_)

Off-white solid; mp 226–228 °C; ^1^H NMR (600 MHz, DMSO-*d*_6_): *δ* = 12.06 (s, 1H), 11.02 (d, *J* = 1.8 Hz, 1H), 7.49–7.11 (m, 5H), 5.32–5.13 (m, 1H). ppm; ^13^C NMR(151 MHz, DMSO-*d*_6_)): *δ* = 162.13, 158.68, 153.03, 149.46, 135.87, 135.70, 134.11, 130.33, 129.26, 128.57, 118.46, 86.07, 54.09, 32.09 ppm; FT-IR (*ν*, cm^−1^): 3389 (Primary N–H), 3213 (Amide N–H), 2196 (C≡N), 1717 &1673 (C=O str), 1096 (C–O–C); HRMS (ESI) *m*/*z* [M + H]^+^ calcd for C_14_H_9_Cl_2_N_4_O_3_^+^:349.9973 found: 349.9971.

### 4.6 Computational studies

#### 4.6.1 Density functional theory (DFT) calculations

The initial geometries of the synthesised pyrano[2,3-*d*]pyrimidinone derivatives (2a–2h) were constructed using the Avogadro software.^30^ Conformational analysis was performed using the conformer–rotamer sampling tool (CREST) at the GFN-xTB level to efficiently sample the potential energy surface (PES) and identify low-energy conformers.^31^ PES exploration was performed for all synthesised molecules (2a–2h), PES exploration was performed by scanning the key dihedral angles relevant to ring flexibility. This sampling approach enabled the identification of the most stable conformers and energetically feasible rotamers based on the free energy rankings. The conformers with the lowest energy identified by CREST were then used as the initial geometries for comprehensive geometry optimisation and electronic structure analysis using ORCA 6.0.1, applying the B3LYP/def2-TZVP theoretical framework.^32,33^ This process utilised the def2/J auxiliary basis set within the resolution of the identity (RI) approximation.^34–37^ The optimised geometries of the molecules are shown in Fig. S33. Following geometry optimisation, the NMR shielding tensors in DMSO medium and IR vibrational frequencies were calculated to corroborate the experimental assignments.^38,39^ Moreover, the electronic structure parameters and global reactivity descriptors were derived using the Koopmans' theorem.^40^ Natural Bond Orbital (NBO) analysis was performed using NBO 7.0 interfaced with ORCA to analyse intra- and intermolecular donor and acceptor interactions and evaluate the second-order perturbation energies.^41,42^ All NBO calculations were performed at the B3LYP/def2-TZVP level using the TightSCF and RI (def2/J) settings.^43^ Molecular visualisations, including HOMO–LUMO surfaces and Molecular Electrostatic Potential (MEP) maps, were generated using Chemcraft.^44^

#### 4.6.2 Molecular docking

Molecular docking analyses were performed to evaluate the binding strengths and interaction characteristics of the synthesised ligands 2a–2h with the reference drug afatinib, focusing on the ERK2 serine/threonine kinase domain (PDB ID: 4ZXT).^45^ The crystal structure of the protein, resolved at 1.50 Å, was obtained from the RCSB Protein Data Bank.^46^ Protein preparation was performed using AutoDock Tools 1.5.7.^47^

All ligand structures were first geometry-optimised using ORCA 6.0.1 (B3LYP/def2-TZVP). The optimised geometries were converted into 3D structures and further processed for docking by assigning Gasteiger charges and defining rotatable bonds using Open Babel,^48^ RDKit,^49^ and Meeko^50^ in a Google Colab environment. The final ligand files were prepared in PDBQT format.

Docking simulations were performed using AutoDock Vina.^51^ A grid box was defined to encompass the active site of 4ZXT, ensuring coverage of all key residues involved in ligand binding. Each ligand was docked independently, and the lowest-energy binding pose was selected based on the binding affinity and favourable interaction profiles. The resulting protein-ligand complexes were analysed using Discovery Studio Visualiser^52^ and UCSF ChimeraX^53^ to examine the binding orientation, hydrophobic contacts, and hydrogen bonding within the active site.

#### 4.6.3 Molecular dynamic simulation

Molecular dynamics simulations were performed using GROMACS version 2024.4, which was installed and run in a Google Colab Pro environment.^54^ The simulation employed two Intel® Xeon® virtual CPUs (2.20 GHz) and an NVIDIA Tesla T4 GPU with CUDA version 12.4 and 16 GB VRAM, which were optimised for parallel GPU-accelerated computation. The protein-ligand complex topology was created using the CHARMM36m all-atom force field.^55^ The ligand topology was generated using the CGenFF server and converted into a format compatible with the GROMACS software. The complex was immersed in a triclinic TIP3P water box.^56^ Na^+^ and Cl^−^ counter ions were added to neutralise the solvated system. Energy minimisation was performed using the steepest descent algorithm for 500 000 steps or until a tolerance of 1000 kJ mol^−1^ nm^−1^ was achieved. This was followed by position-restrained equilibration under the NVT and NPT ensembles at 298 K and 1 atm pressure, utilising the V-rescale thermostat and Parrinello-Rahman barostat, respectively.^57,58^ Post-simulation analyses, were primarily performed using the built-in GROMACS utilities, whereas the processed data were further visualised and plotted using Python 3 libraries. Principal Component Analysis (PCA), FEL, and MM/GBSA analyses were performed on the 100 ns MD trajectories to evaluate the essential dynamics, conformational free-energy basins, and binding free energies of the 4ZXT–ligand complexes. The free energy landscape (FEL) and principal component analysis (PCA) were constructed from covariance matrices of the backbone atoms, whereas MM/GBSA calculations were conducted using gmx_MMPBSA to estimate the van der Waals, electrostatic, and solvation energy contributions.

#### 4.6.4 Pharmacokinetics and drug-likeness prediction

The SwissADME portal (http://www.swissadme.ch/)^59^ and MolSoft drug-likeness calculator (https://molsoft.com/mprop/)^60^ were used to evaluate the pharmacokinetic characteristics and drug-likeness of the synthesised scaffolds (2a–2h). The SMILES notation for each compound was used as the input. SwissADME was used to predict the essential pharmacokinetic attributes. MolSoft web server was used to compute drug-likeness scores. These computational predictions are instrumental in evaluating the oral druggability and pharmacokinetic potential of designed compounds at an early stage of development.

### 4.7 MTT assay

#### 4.7.1 A549 cell culture

A549 cells (ATCC) were grown in DMEM/F-12 medium supplemented with 10% heat-inactivated foetal bovine serum (FBS), 100 IU per mL penicillin, and 100 µg mL^−1^ of streptomycin. The cells were cultured at 37 °C in a humidified atmosphere of 5% CO_2_ and subcultured when they reached confluence. At the time of subculture, the A549 cells were treated with a solution containing 0.2% trypsin, 0.02% EDTA, and 0.05% glucose dissolved in PBS. Following treatment, cells were centrifuged to determine cell viability. Approximately 5 × 10^4^ cells were placed per well in 96-well plates and incubated at 37 °C under normal culture conditions for 24 hours.^61^

#### 4.7.2 MTT cell proliferation assay

A549 cells were plated at a concentration of 5 × 10^4^ cells/well into 96-well microtitre plates and cultured for 24 h in DMEM/F12 containing 10% FBS at the appropriate temperature (37 °C), atmospheric oxygen levels (5%), and CO_2_ levels to facilitate cell attachment. The medium was replaced with a fresh medium containing different amounts of various test compounds and cultured for an additional 24 h. Following this incubation period, 100 µL MTT solution (5 mg mL^−1^ in PBS) was added to each well of the plate and incubated for 4 h to permit the production of insoluble purple crystals known as formazan. The formazan crystals were dissolved by removing the supernatant and adding 100 µL of DMSO, followed by measurement of the absorbance at 590 nm using a microplate reader. Percentages of growth inhibition were determined and IC_50_ values were determined based on data from dose–response curves.^62^

## Author contributions

All authors contributed significantly to the design, execution, and interpretation of experiments. Sathiaseelan Perumal: writing – original draft, methodology, investigation, and data curation. Perumal Muthuraja: resources, writing – review & editing M. Sasikumar: writing – review & editing. R. Hari Krishna: investigation Manisankar Paramasivam: project administration and resources, conceptualization. Viswanathan Subramanian: project administration and resources, writing – review & editing, and supervision.

## Conflicts of interest

There are no conflicts of interest to declare.

## Supplementary Material

RA-016-D5RA08635C-s001

## Data Availability

All data generated or analysed during this study are included in this published article and its supporting information (SI). Supplementary information is available. See DOI: https://doi.org/10.1039/d5ra08635c.
