# Supplementary material for: Ag2WO4 nanocatalyst-driven green synthesis of pyrano[2,3-d]pyrimidinones: an integrated experimental, DFT, and cytotoxic investigation
Source: RSC Adv. 2026 Jan 16;16(4):3580–601. doi: 10.1039/d5ra08635c (PMC12809388; doi:10.1039/d5ra08635c)
Supplement: RA-016-D5RA08635C-s001 [file RA-016-D5RA08635C-s001.pdf]

Supplementary Information (SI) for RSC Advances.
This journal is © The Royal Society of Chemistry 2026

Supporting Information

Ag_2WO_4 nanocatalyst-driven green synthesis of pyrano[2,3-d]pyrimidinones: an integrated experimental, DFT, and cytotoxic investigation

Sathiaseelan Perumal [a,b], Perumal Muthuraja [c], M. Sasikumar [b], R. Hari Krishna [d], Paramasivam Manisankar [a*], Viswanathan Subramanian [a*]

[a] Department of Industrial Chemistry, Alagappa University, Karaikudi – 630 006, Tamil Nadu, India.

[b] Department of Chemistry, Bishop Heber College, Tiruchirappalli – 620 017, Tamil Nadu, India.

[c] Department of Chemistry, Indian Institute of Technology Guwahati, Guwahati – 781039, India.

[d] Department of Chemistry, M.S. Ramaiah Institute of Technology, Bangalore-560 054, India

*Correspondence: manisankarp@alagappauniversity.ac.in (P.M), rsviswa@gmail.com (V.S)

<u>Table of Contents – Supplementary Information</u>

1. NMR and IR Spectra

Fig. S1–S3: NMR (^{1}H, ^{13}C) and IR spectra of compound 2a

Fig. S4–S6: NMR (^{1}H, ^{13}C) and IR spectra of compound 2b

Fig. S7–S9: NMR (^{1}H, ^{13}C) and IR spectra of compound 2c

Fig. S10–S12: NMR (^{1}H, ^{13}C) and IR spectra of compound 2d

Fig. S13–S15: NMR (^{1}H, ^{13}C) and IR spectra of compound 2e

Fig. S16–S18: NMR (^{1}H, ^{13}C) and IR spectra of compound 2f

Fig. S19–S21: NMR (^{1}H, ^{13}C) and IR spectra of compound 2g

Fig. S22–S24: NMR (^{1}H, ^{13}C) and IR spectra of compound 2h

Fig. S25-S32**:** Comparative IR spectra of compound 2a-2h**:** Experimental vs Theoretical

2. DFT

Fig. S33 (HOMO, LUMO) orbitals, Molecular Electrostatic Potential plots (MEP) and optimized structures (2a–2h and afatinib) at B3LYP/def2-TZVP level in gas phase.

2. Molecular Docking

Fig. S34 AutoDock Vina binding affinities (kcal/mol) of synthesised pyrano[2,3-d]pyrimidinone derivatives derivatives (1a–1j) and the reference drug afatinib against PDB ID: 4ZXT.

3. MTT Assay

Fig. S35 Linear regression analysis correlating the molecular docking binding affinities (kcal/mol) with the experimental IC_{50} values (μM) of the synthesized compounds (2a–2h and afatinib).

Fig. S36 *MTT assay evaluation of cytotoxic effects of synthesized compounds (2a–2h) on A549 cell line.*

6. Tables

Table S1: Experimental and calculated ^{1H} NMR chemical shifts (□ in ppm) of compound **2a** at the B3LYP/def2-TZVP level of theory in DMSO phase

Table S2: Experimental and calculated ^{1H} NMR chemical shifts (□ in ppm) of compound **2b** at the B3LYP/def2-TZVP level of theory in DMSO phase

Table S3: Experimental and calculated ^{1H} NMR chemical shifts (□ in ppm) of compound **2c** at the B3LYP/def2-TZVP level of theory in DMSO phase

Table S4: Experimental and calculated ^{1H} NMR chemical shifts (□ in ppm) of compound **2d** at the B3LYP/def2-TZVP level of theory in DMSO phase

Table S5: Experimental and calculated ^{1H} NMR chemical shifts (□ in ppm) of compound **2e** at the B3LYP/def2-TZVP level of theory in DMSO phase

Table S6: Experimental and calculated ^{1H} NMR chemical shifts (□ in ppm) of compound **2f** at the B3LYP/def2-TZVP level of theory in DMSO phase

Table S7: Experimental and calculated ^{1H} NMR chemical shifts (□ in ppm) of compound **2g** at the B3LYP/def2-TZVP level of theory in DMSO phase

Table S8: Experimental and calculated ^{1H} NMR chemical shifts (□ in ppm) of compound **2h** at the B3LYP/def2-TZVP level of theory in DMSO phase

Table S9: Experimental and calculated ^{13}C NMR chemical shifts (□ in ppm) of compound **2a** at the B3LYP/def2-TZVP level of theory in DMSO phase

Table S10: Experimental and calculated ^{13}C NMR chemical shifts (□ in ppm) of compound **2b** at the B3LYP/def2-TZVP level of theory in DMSO phase

Table S11: Experimental and calculated ^{13}C NMR chemical shifts (□ in ppm) of compound **2c** at the B3LYP/def2-TZVP level of theory in DMSO phase

Table S12: Experimental and calculated ^{13}C NMR chemical shifts (□ in ppm) of compound **2d** at the B3LYP/def2-TZVP level of theory in DMSO phase

Table S13: Experimental and calculated ^{13}C NMR chemical shifts (□ in ppm) of compound **2e** at the B3LYP/def2-TZVP level of theory in DMSO phase

Table S14: Experimental and calculated ^{13}C NMR chemical shifts (□ in ppm) of compound **2f** at the B3LYP/def2-TZVP level of theory in DMSO phase

Table S15: Experimental and calculated ^{13}C NMR chemical shifts (□ in ppm) of compound **2g** at the B3LYP/def2-TZVP level of theory in DMSO phase

Table S16: Experimental and calculated ^{13}C NMR chemical shifts (□ in ppm) of compound **2h** at the B3LYP/def2-TZVP level of theory in DMSO phase

Table S17: Comparative overview of reported catalytic systems for the synthesis of pyrano[2,3-d]pyrimidinone derivatives under different reaction conditions, along with the present Ag_2WO_4-catalyzed protocol.

Table S18**:** Structure-Activity Relationship (SAR) analysis of synthesized pyrano[2,3-d]pyrimidinone derivatives (2a-2h) against the A549 cell line, correlating IC_{50} values, physicochemical descriptors, and docking scores with substituent effects

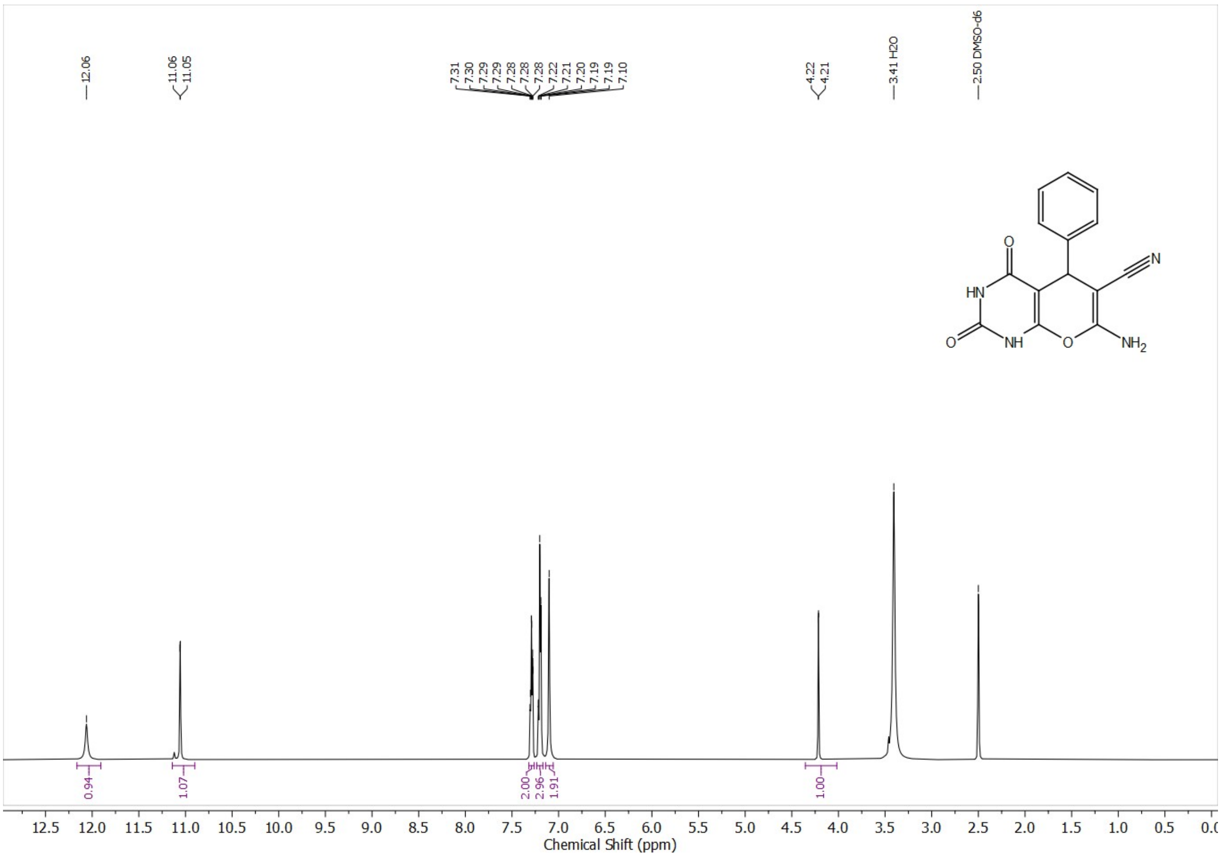

Fig. S1. *^{1}H NMR spectrum of **compound 2a** recorded in DMSO-d₆ (600 MHz).*

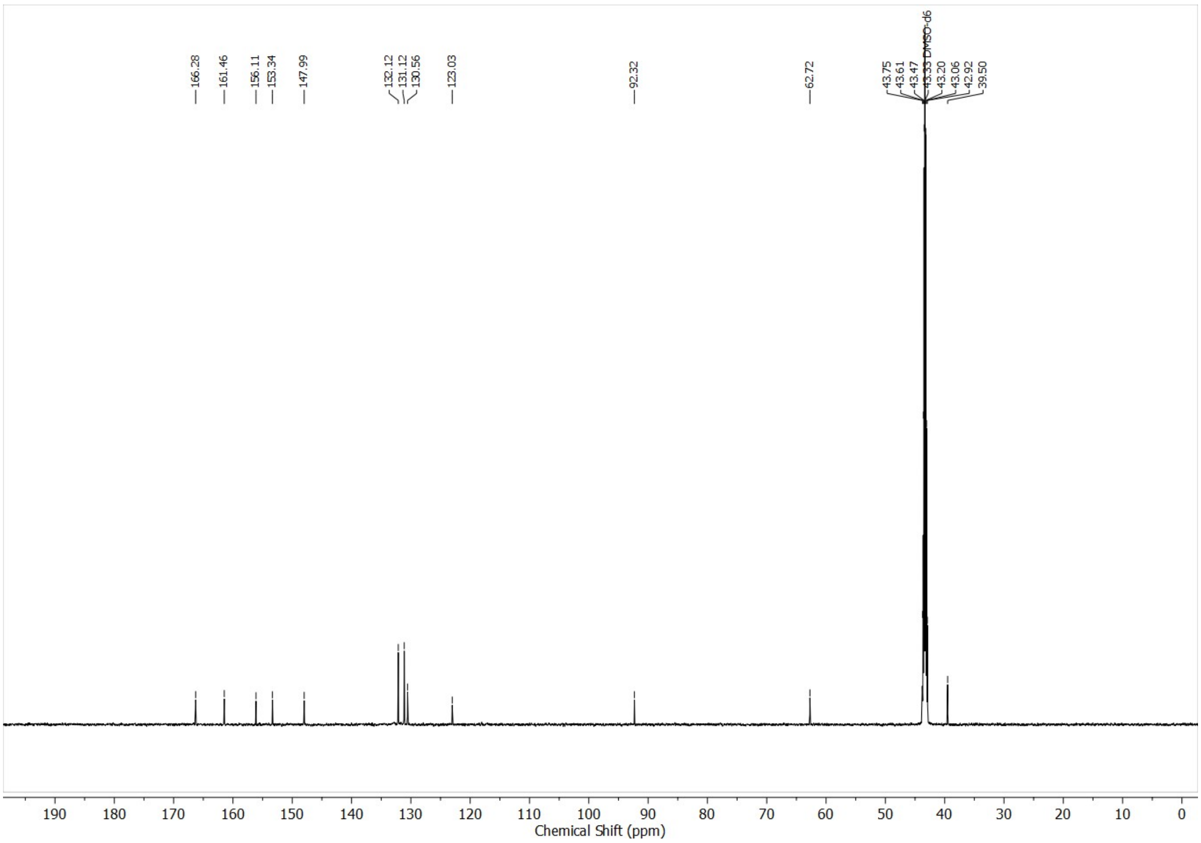

Fig. S2. *^{13}C NMR spectrum of* ***compound 2a*** *in DMSO-d$_6$ (151 MHz).*

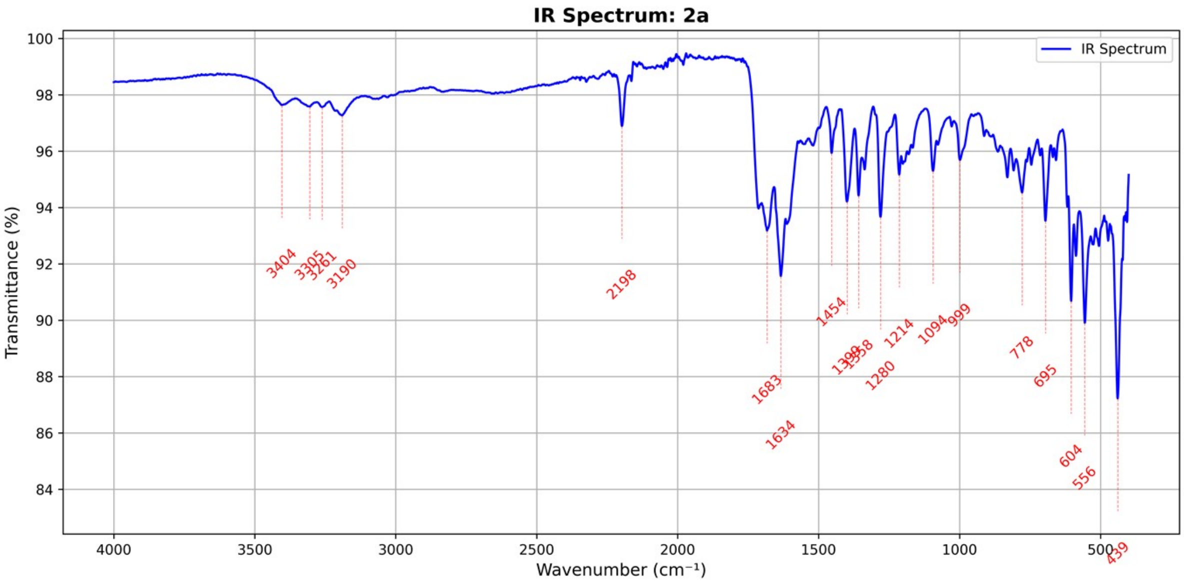

Fig. S3. *IR spectrum of* ***compound 2a*** *(FT-IR).*

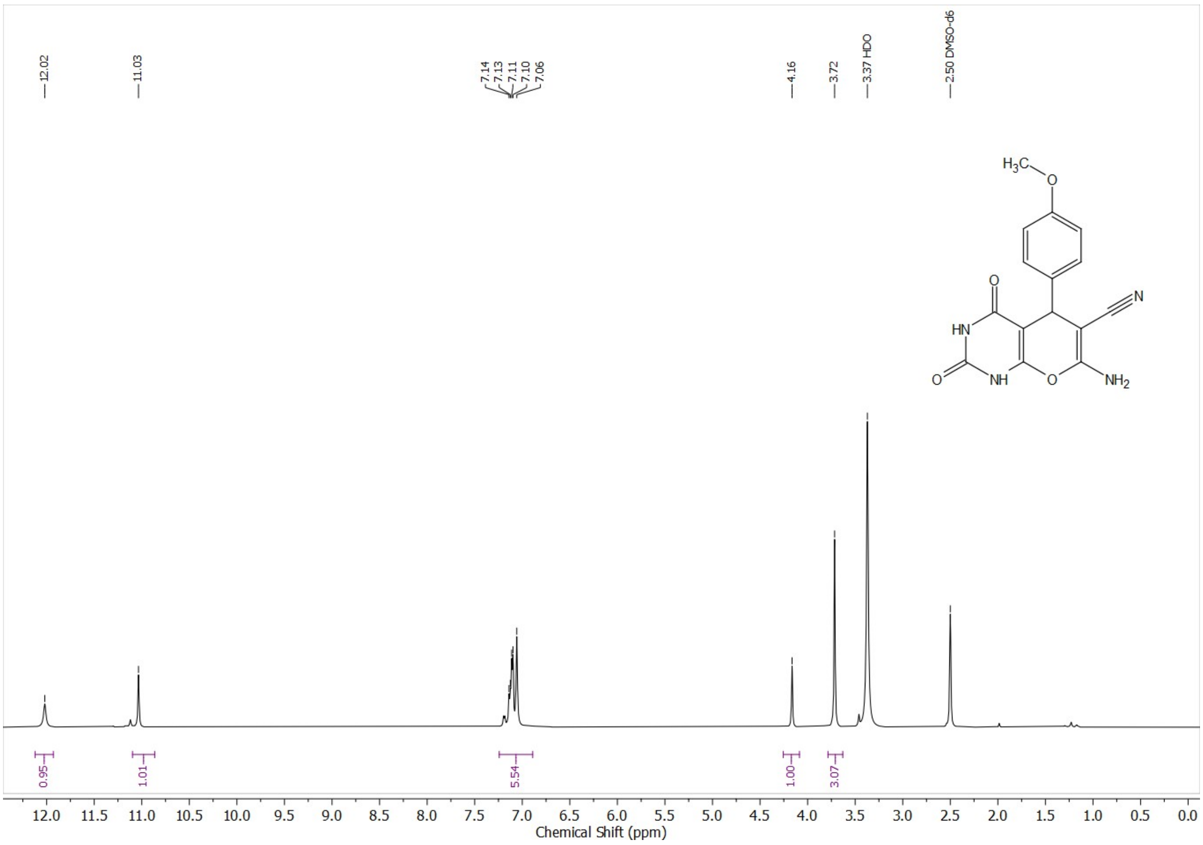

Fig. S4. *^{1}H NMR spectrum of* ***compound 2b*** *recorded in DMSO-d₆ (600 MHz).*

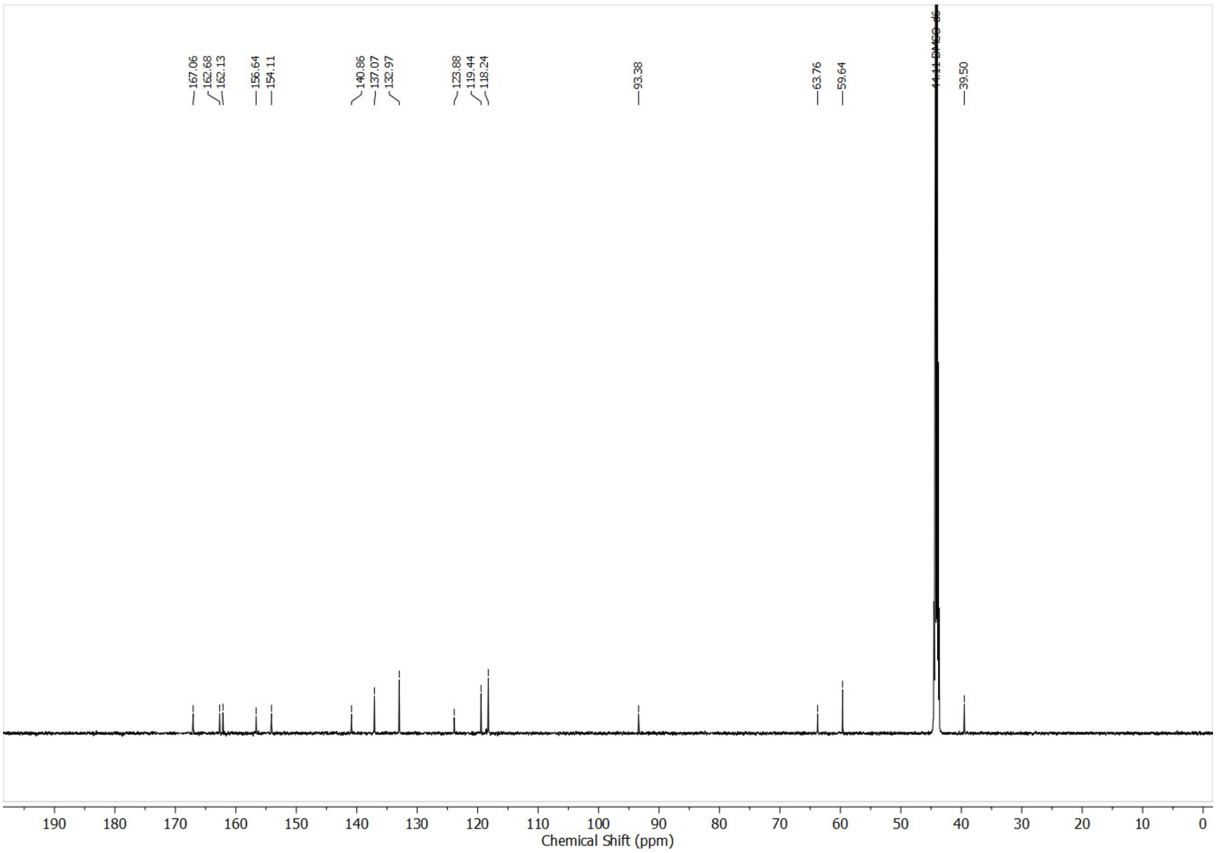

Fig. S5. *^{13}C NMR spectrum of* ***compound 2b*** *in DMSO-d_6 (151 MHz).*

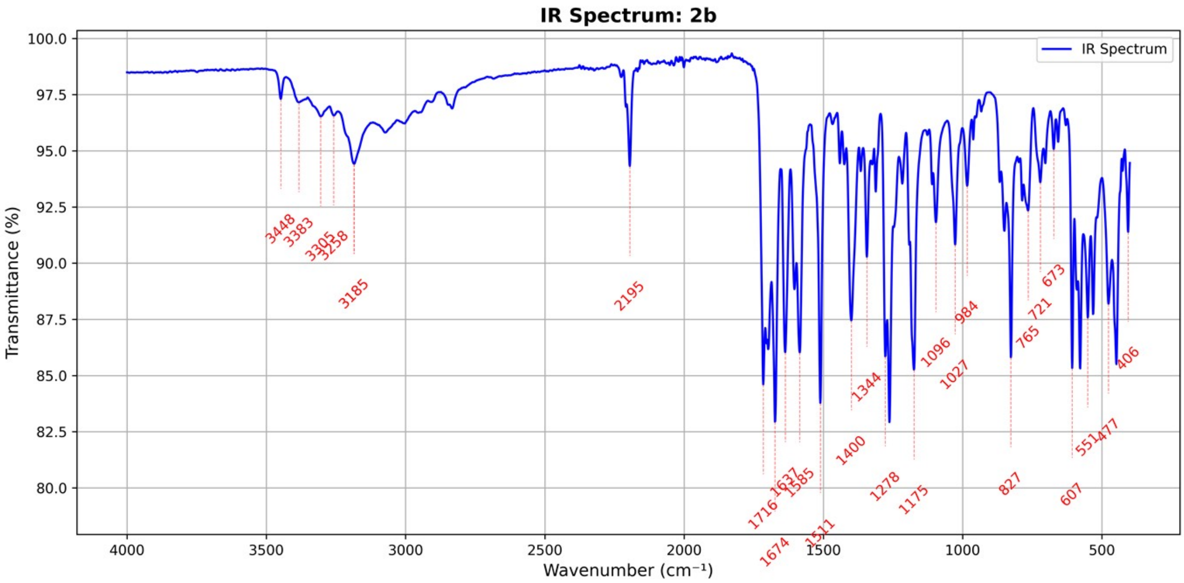

Fig. S6. *IR spectrum of* ***compound 2b*** *(FT-IR).*

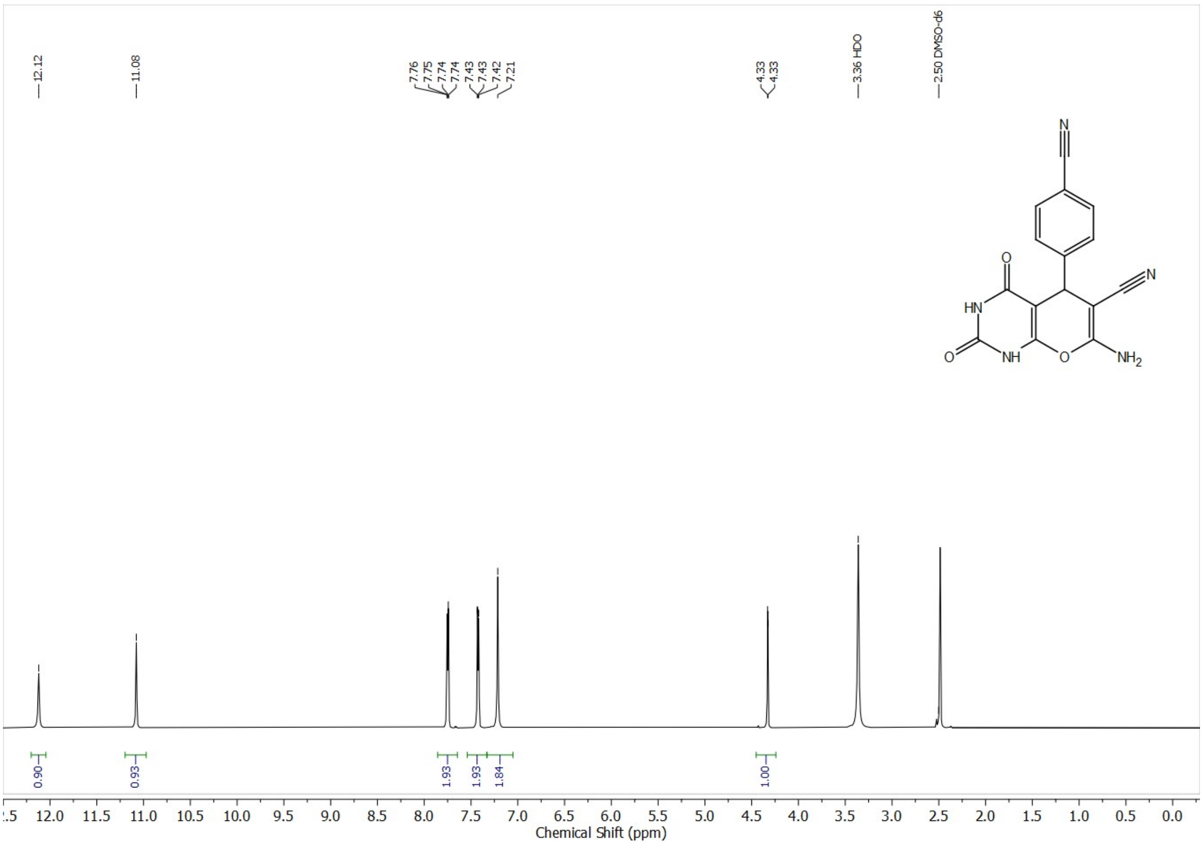

Fig. S7. *^{1}H NMR spectrum of* ***compound 2c*** *recorded in DMSO-d_6 (600 MHz).*

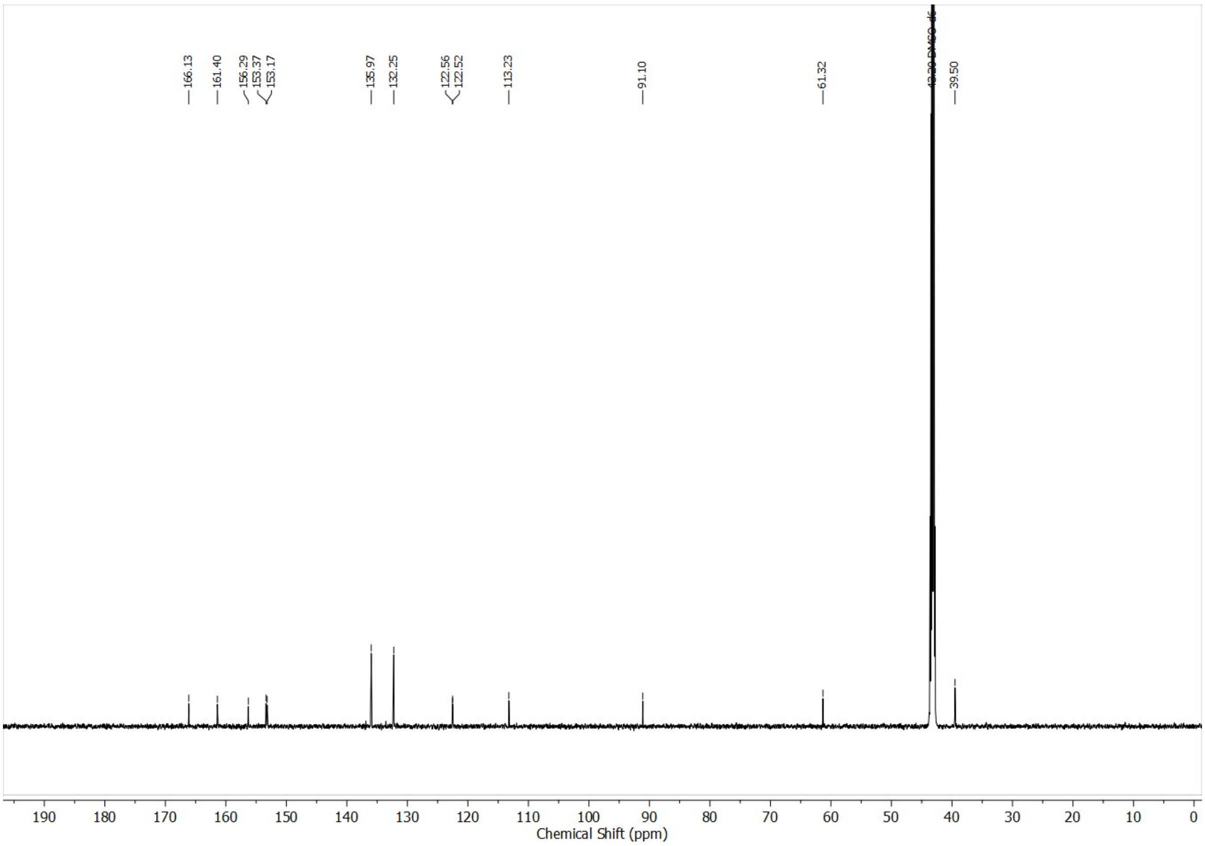

Fig. S8. *^{13}C NMR spectrum of **compound 2c** in DMSO-d_6 (151 MHz).*

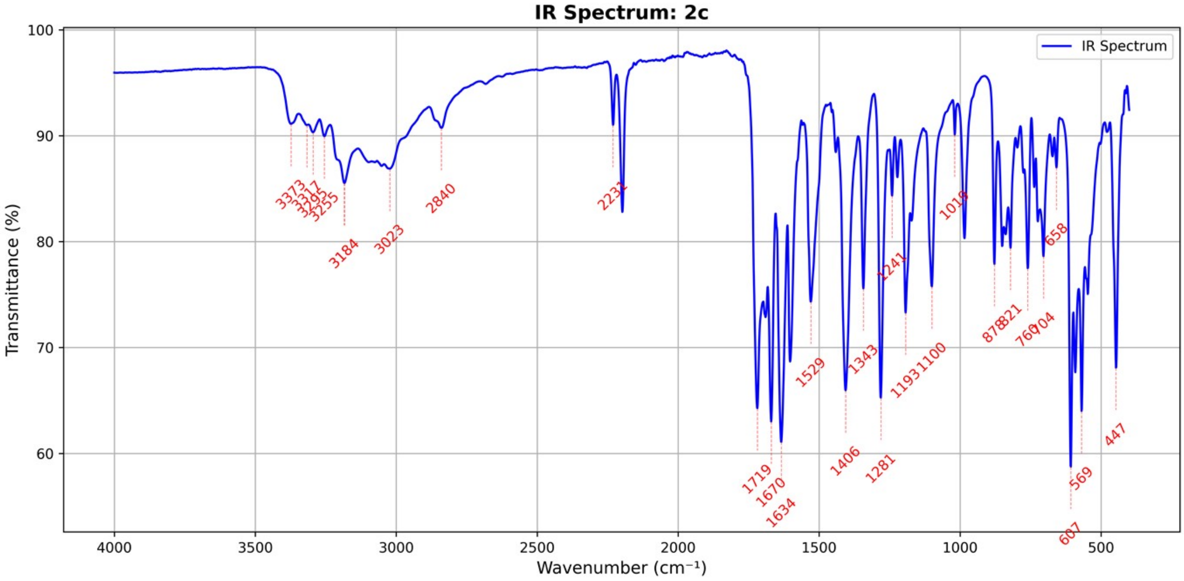

Fig. S9. *IR spectrum of* ***compound 2c*** *(FT-IR).*

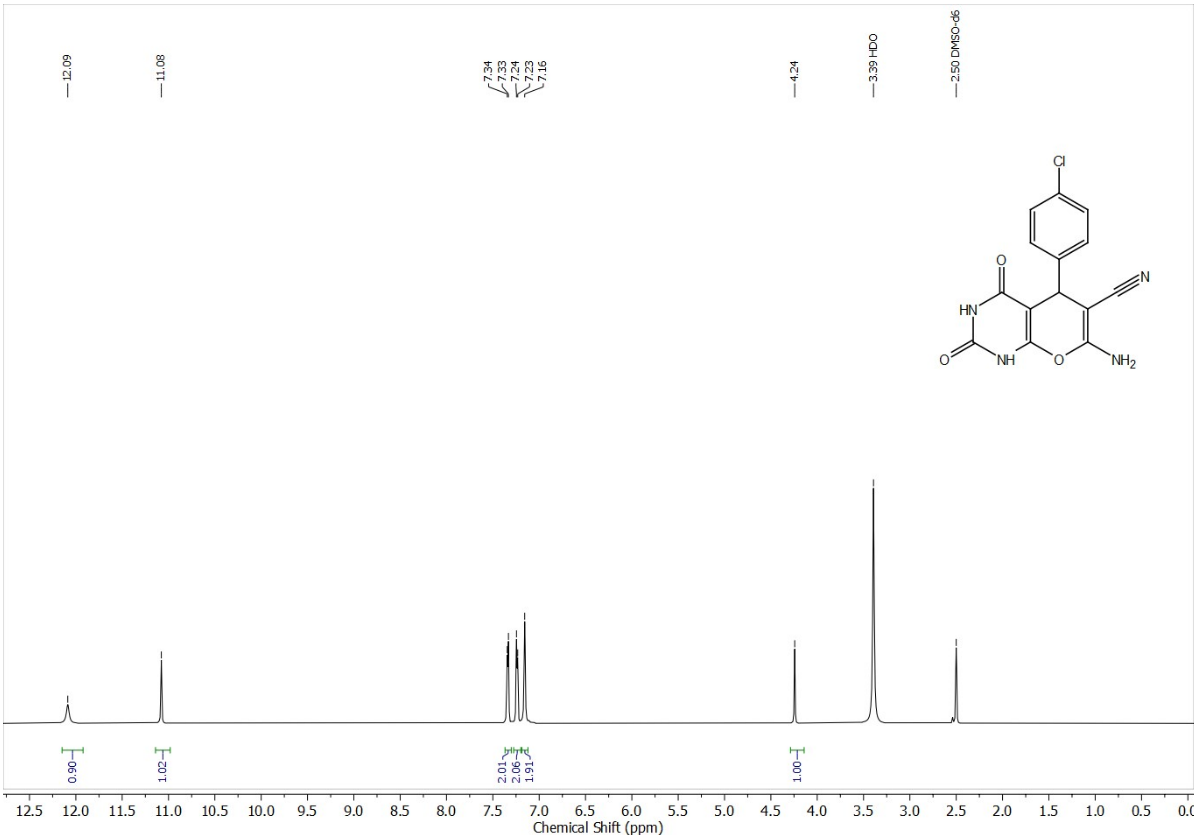

Fig. S10. *^{1}H NMR spectrum of* ***compound 2d*** *recorded in DMSO-d$_6$ (600 MHz).*

\

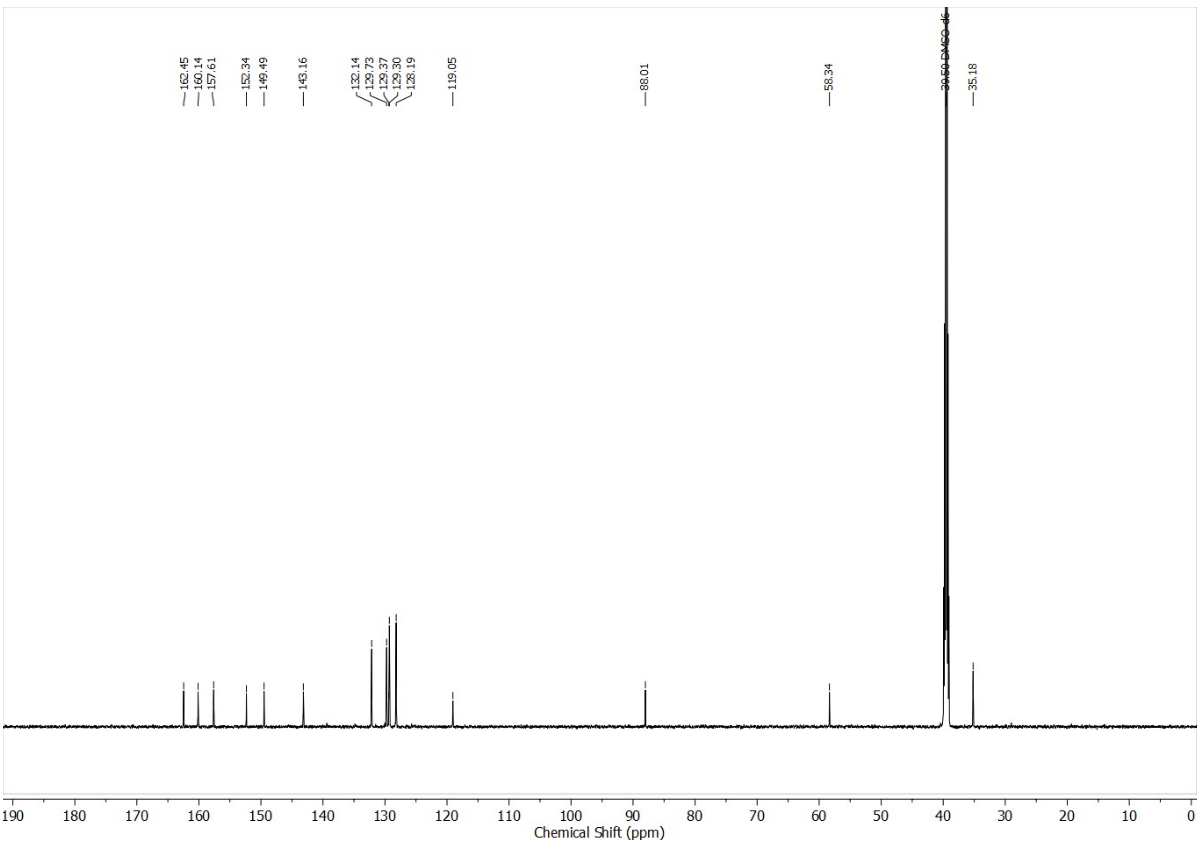

Fig. S11. *^{13}C NMR spectrum of* ***compound 2d*** *in DMSO-d*$_6$ *(151 MHz).*

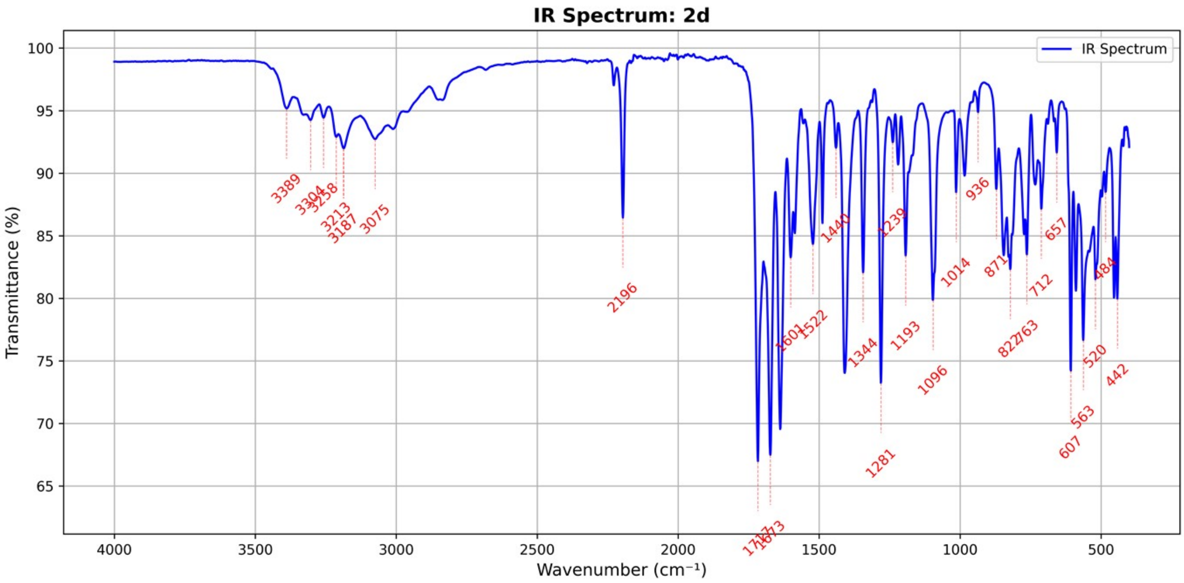

Fig. S12. *IR spectrum of* ***compound 2d*** *(FT-IR).*

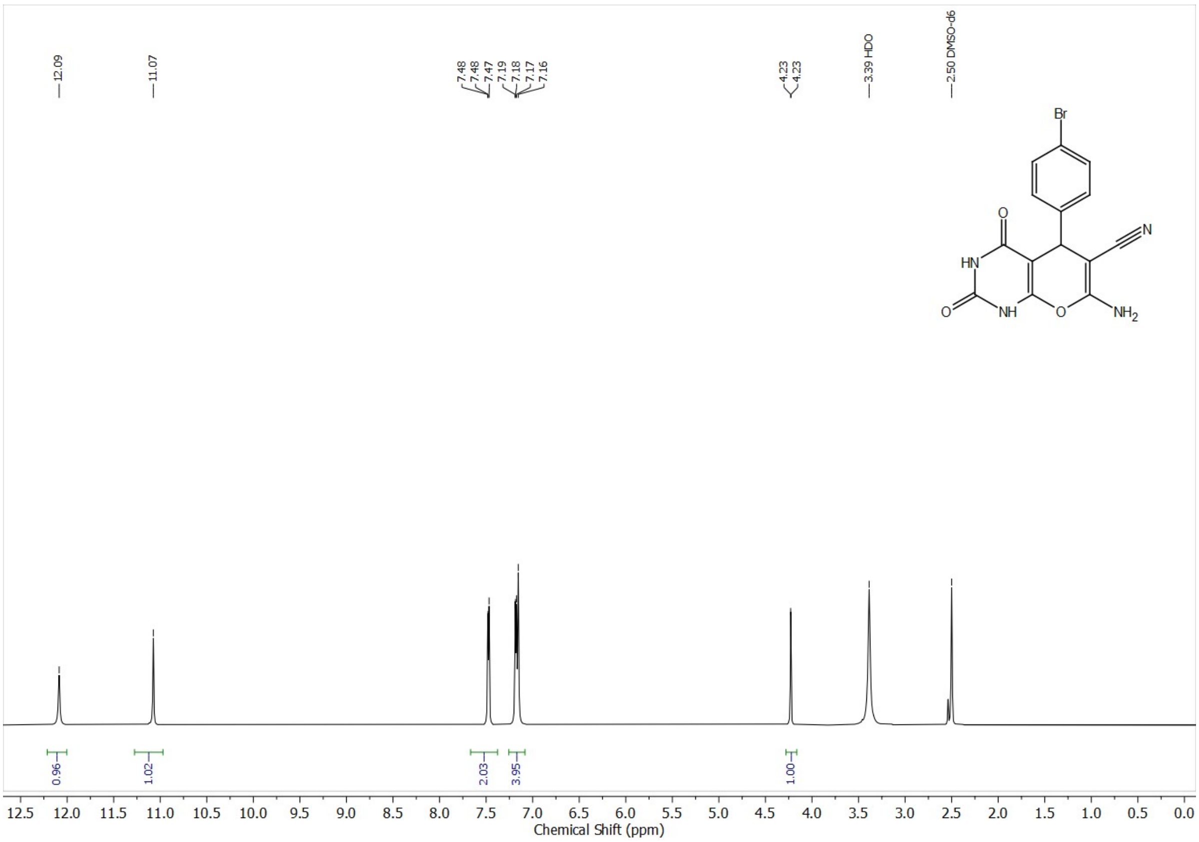

Fig. S13. *^{1}H NMR spectrum of* ***compound 2e*** *recorded in DMSO-d_6 (600 MHz).*

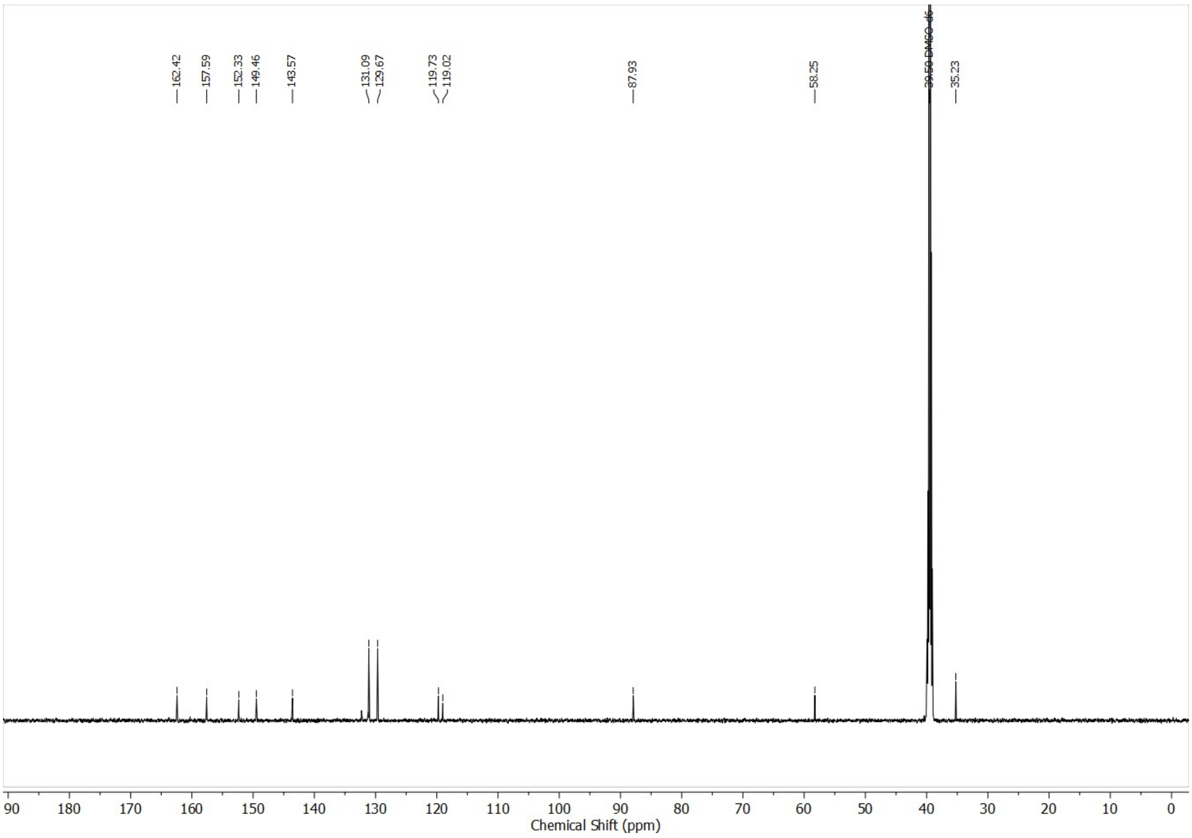

Fig. S14. *^{13}C NMR spectrum of* ***compound 2e*** *in DMSO-d_6 (151 MHz).*

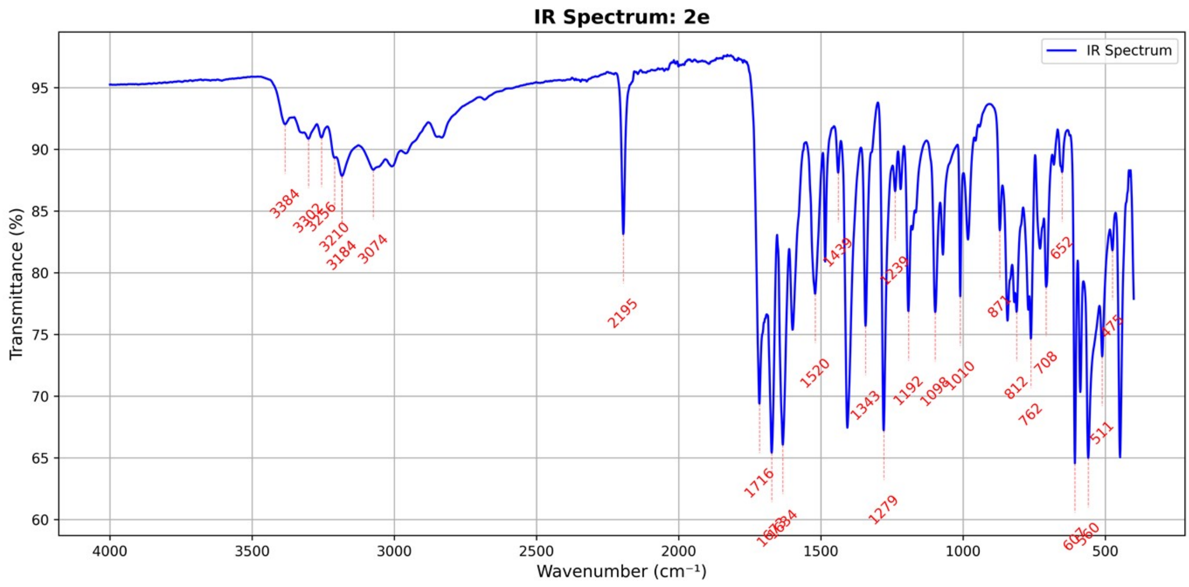

Fig. S15. *IR spectrum of* ***compound 2e*** *(FT-IR).*

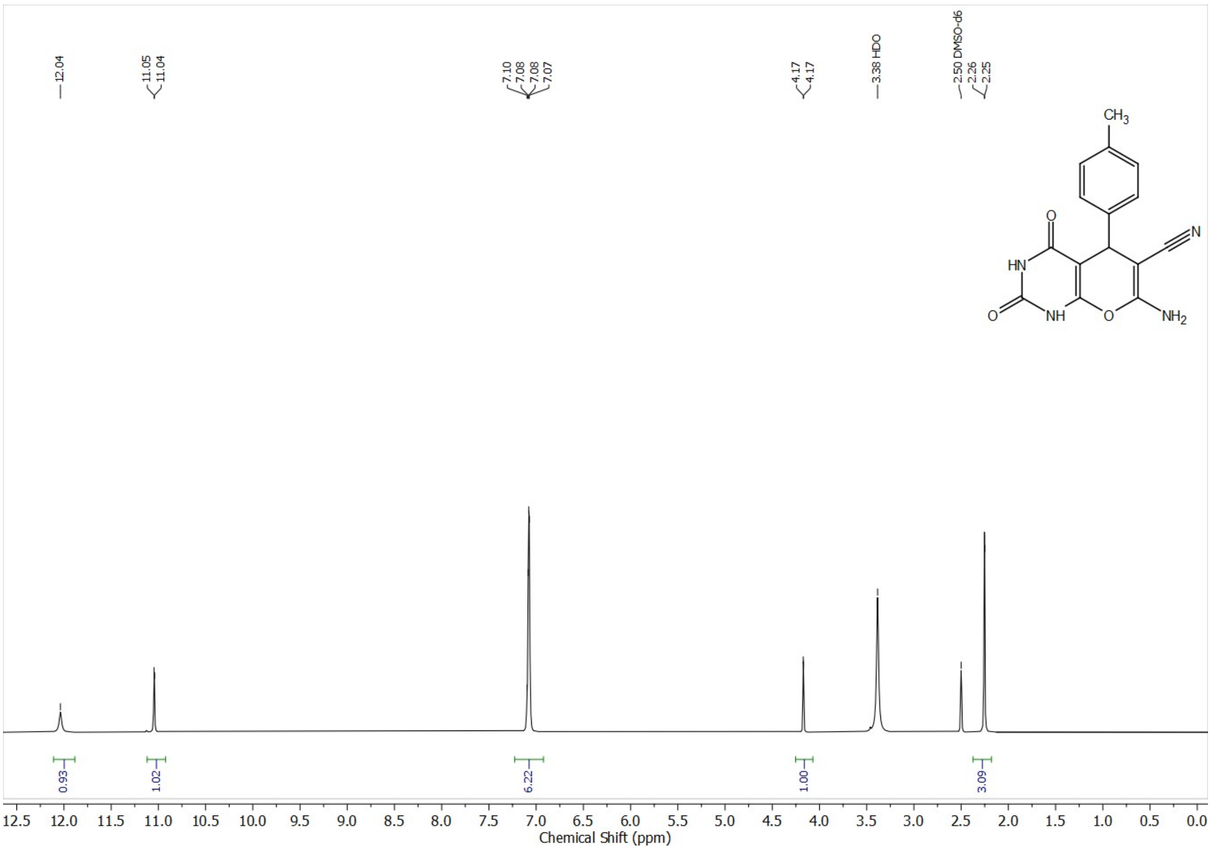

Fig. S16. *^{1}H NMR spectrum of* ***compound 2f*** *recorded in DMSO-d$_6$ (600 MHz).*

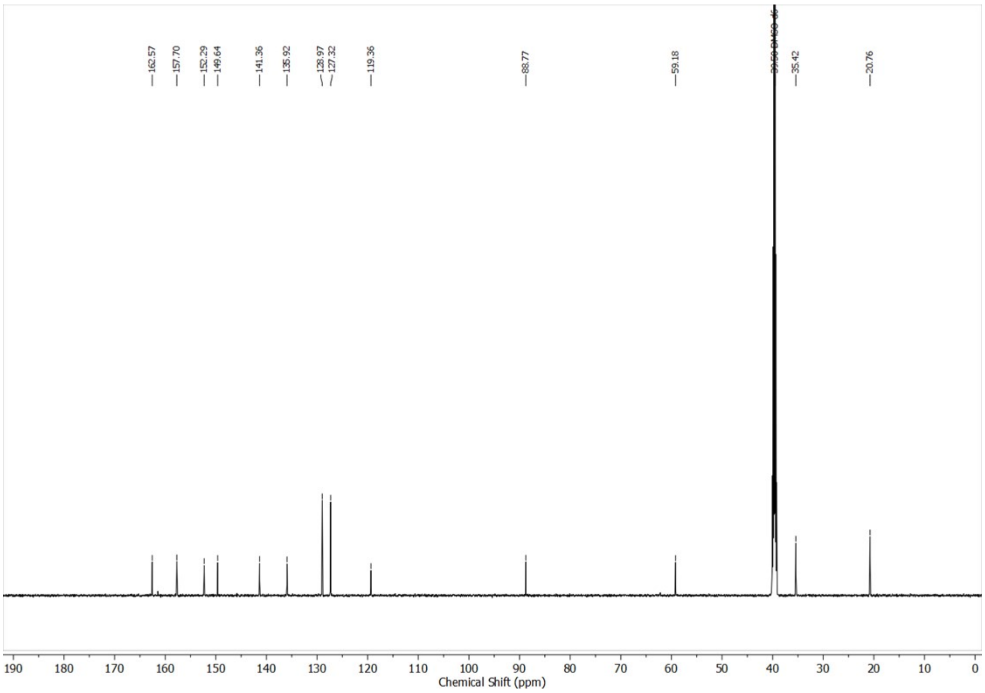

Fig. S17. *^{13}C NMR spectrum of* ***compound 2f*** *in DMSO-d₆ (151 MHz).*

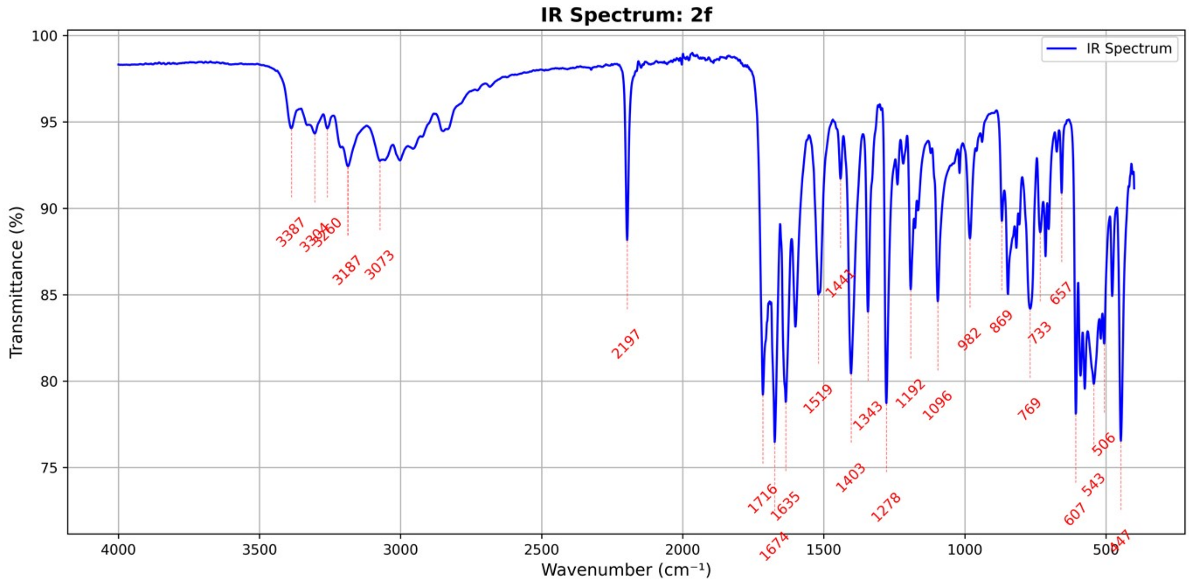

Fig. S18. *IR spectrum of* ***compound 2f*** *(FT-IR).*

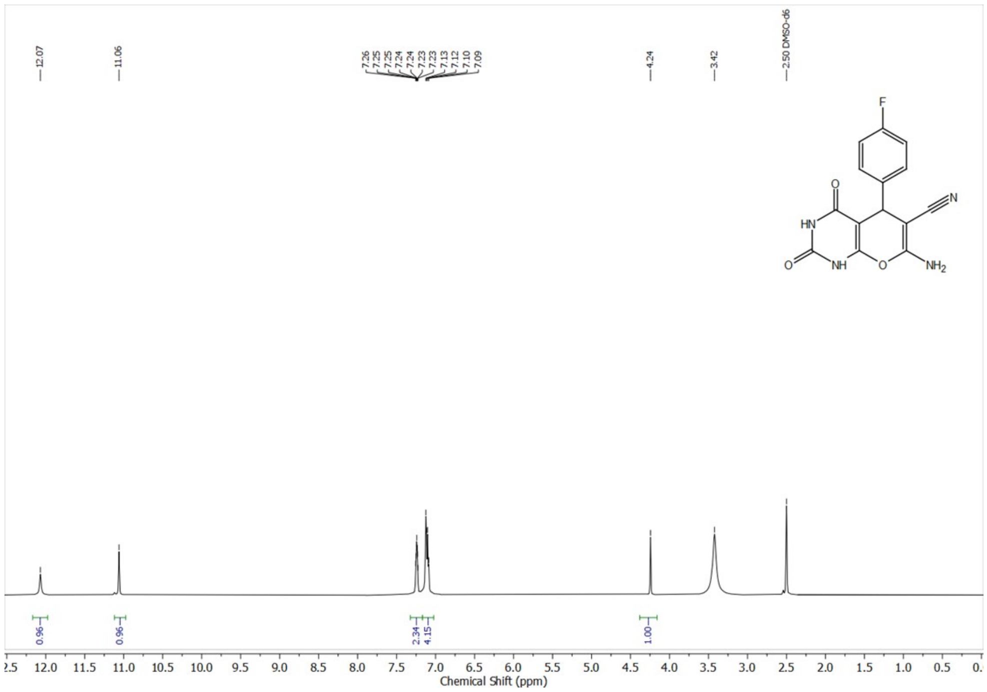

Fig. S19. *^{1}H NMR spectrum of* ***compound 2g*** *recorded in DMSO-d₆ (400 MHz).*

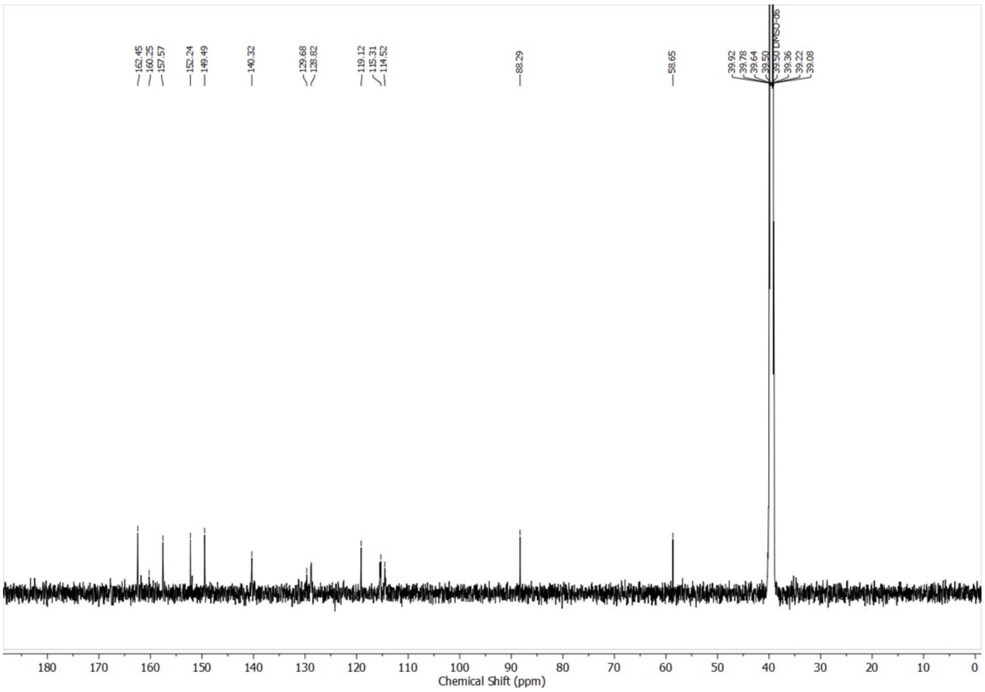

Fig. S20. *^{13}C NMR spectrum of* ***compound 2g*** *in DMSO-d₆ (151 MHz).*

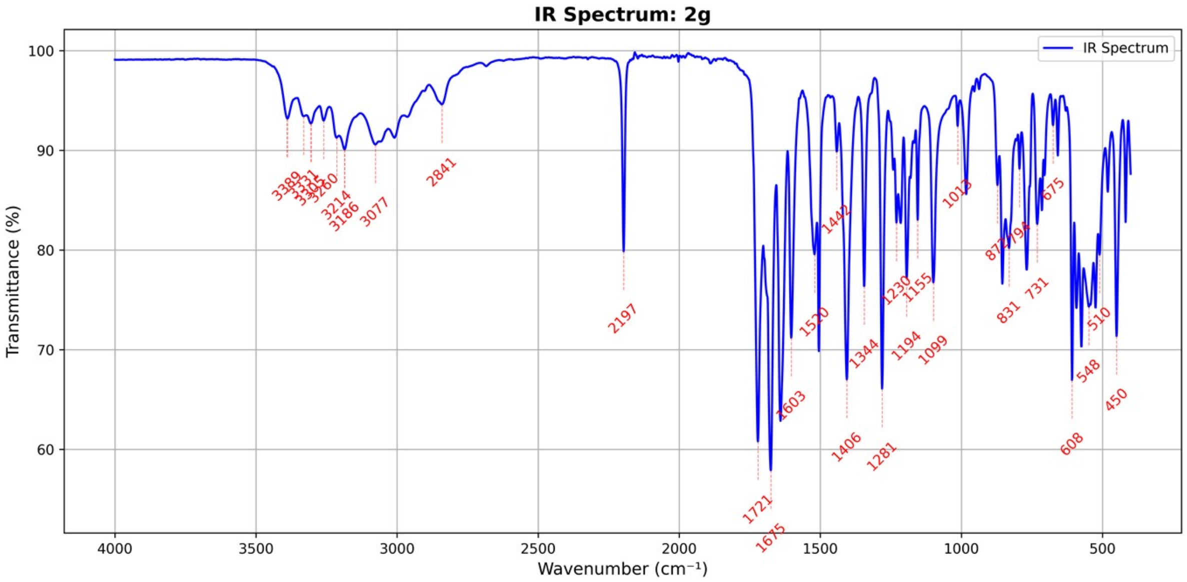

Fig. S21. *IR spectrum of* ***compound 2g*** *(FT-IR).*

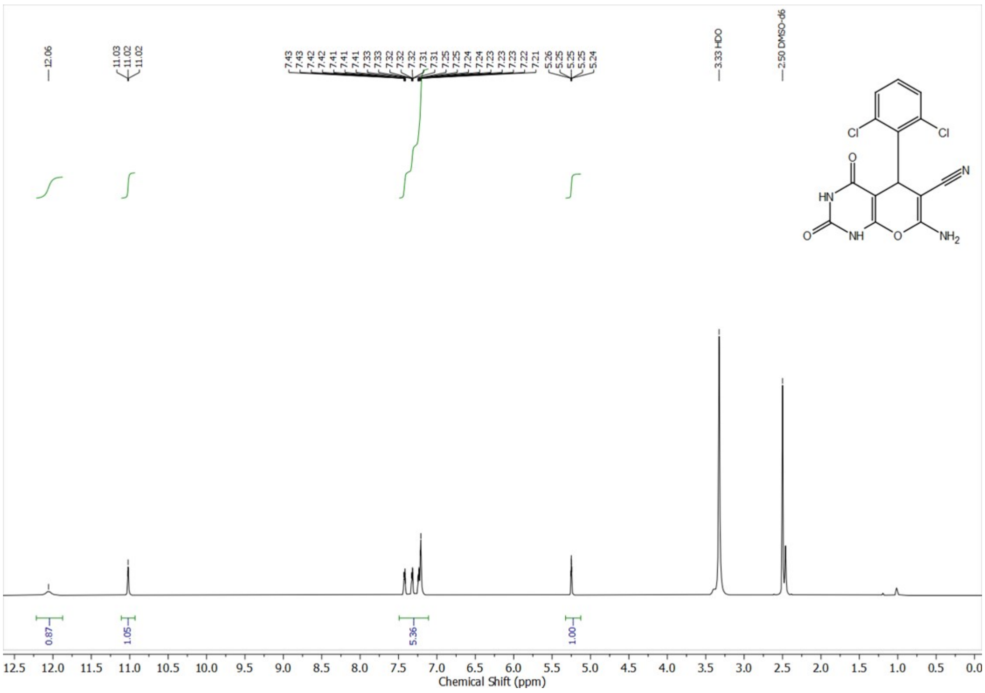

Fig. S22. *^{1}H NMR spectrum of* ***compound 2h*** *recorded in DMSO-d₆ (600 MHz).*

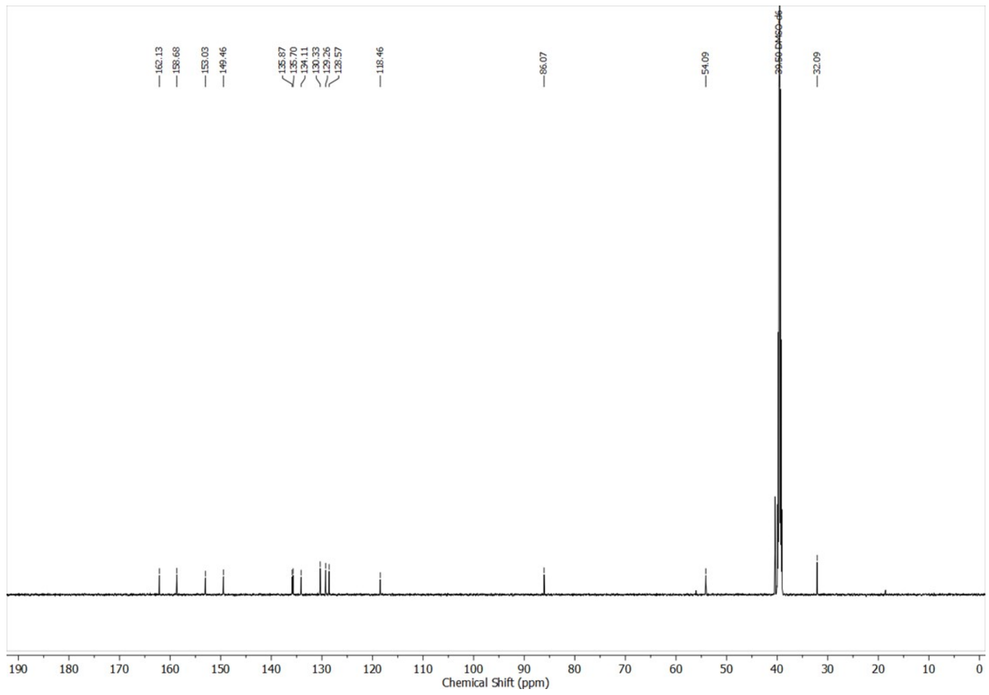

Fig. S23. *^{13}C NMR spectrum of* ***compound 2h*** *in DMSO-d₆ (151 MHz).*

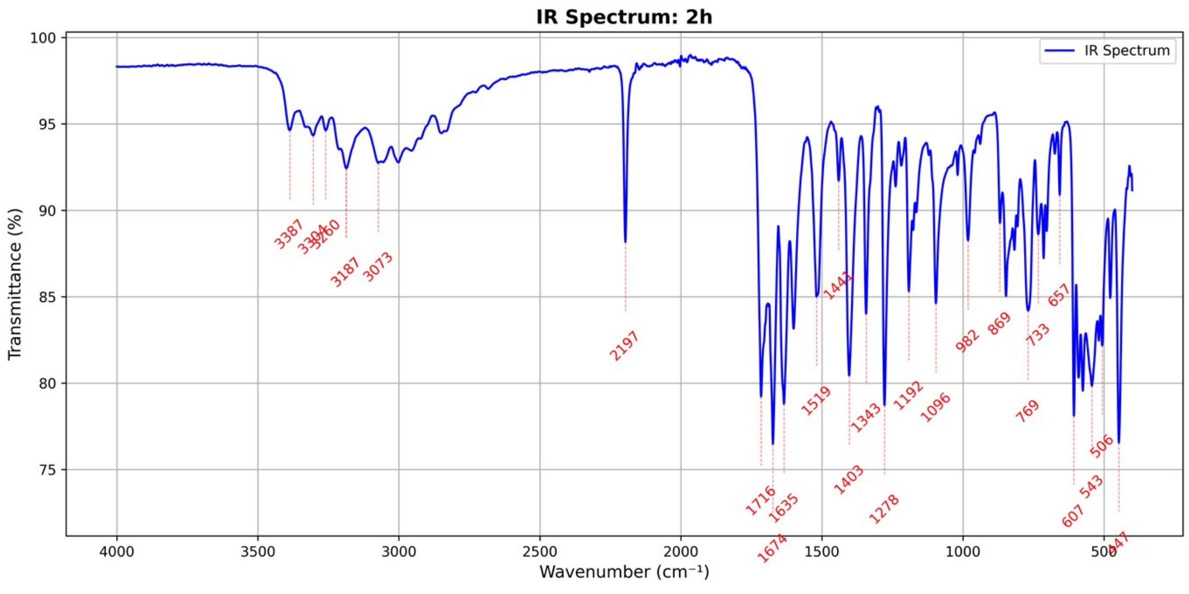

Fig. S24. *IR spectrum of* ***compound 2h*** *(FT-IR).*

Fig. S25 *Comparative IR spectra of compound* ***2a****: experimental (black) vs theoretical (red) transmittance. Theoretical spectrum was computed using DFT (B3LYP/def2-TZVP) and plotted with a Gaussian convolution to simulate peak broadening.*

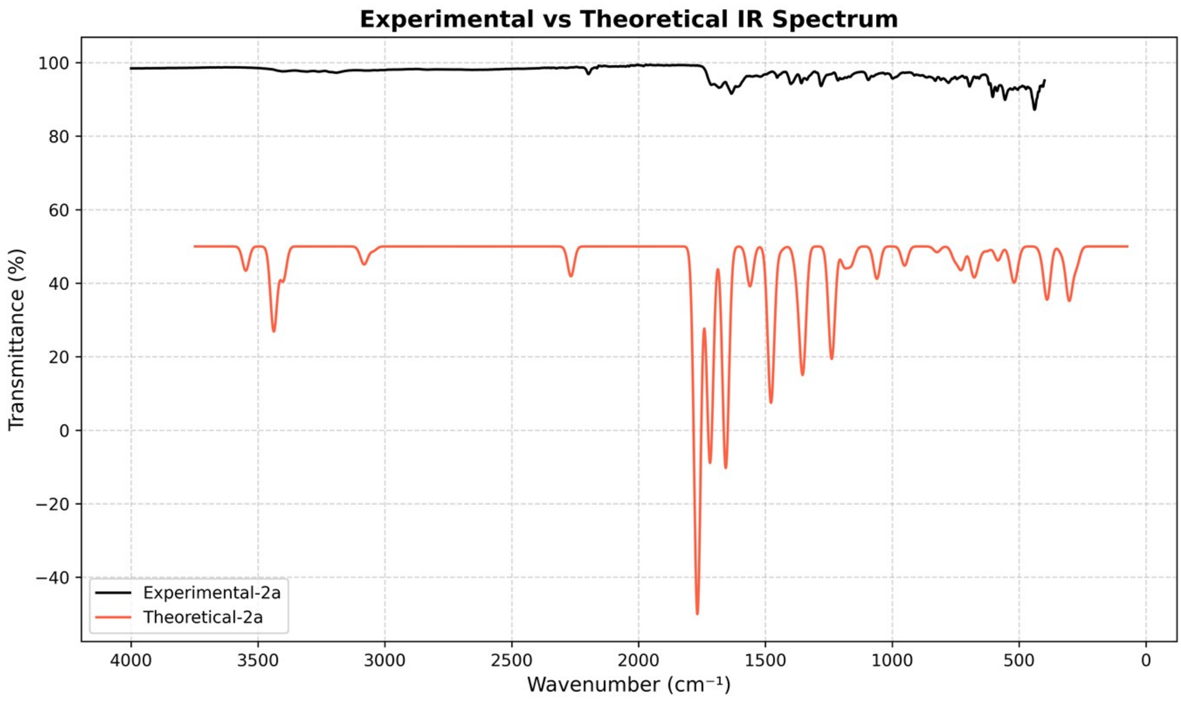

Fig. S26 *Comparative IR spectra of compound* ***2b****: experimental (black) vs theoretical (red) transmittance. Theoretical spectrum was computed using DFT (B3LYP/def2-TZVP) and plotted with a Gaussian convolution to simulate peak broadening.*

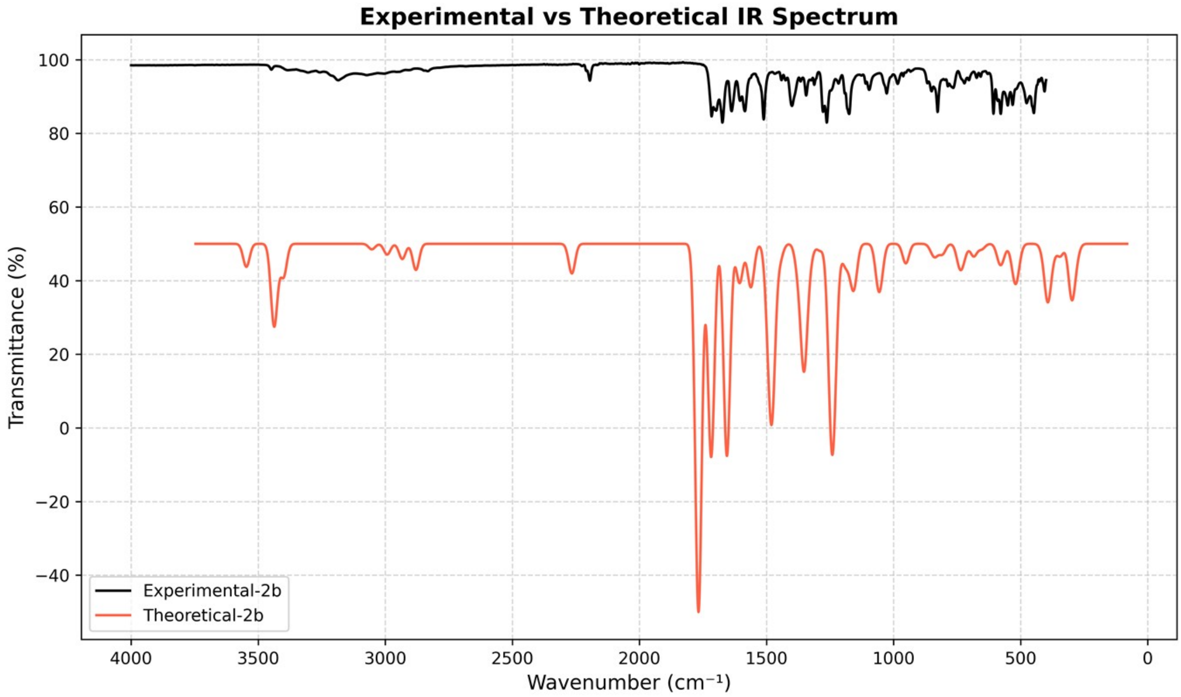

Fig. S27 *Comparative IR spectra of compound* ***2c****: experimental (black) vs theoretical (red) transmittance. Theoretical spectrum was computed using DFT (B3LYP/def2-TZVP) and plotted with a Gaussian convolution to simulate peak broadening.*

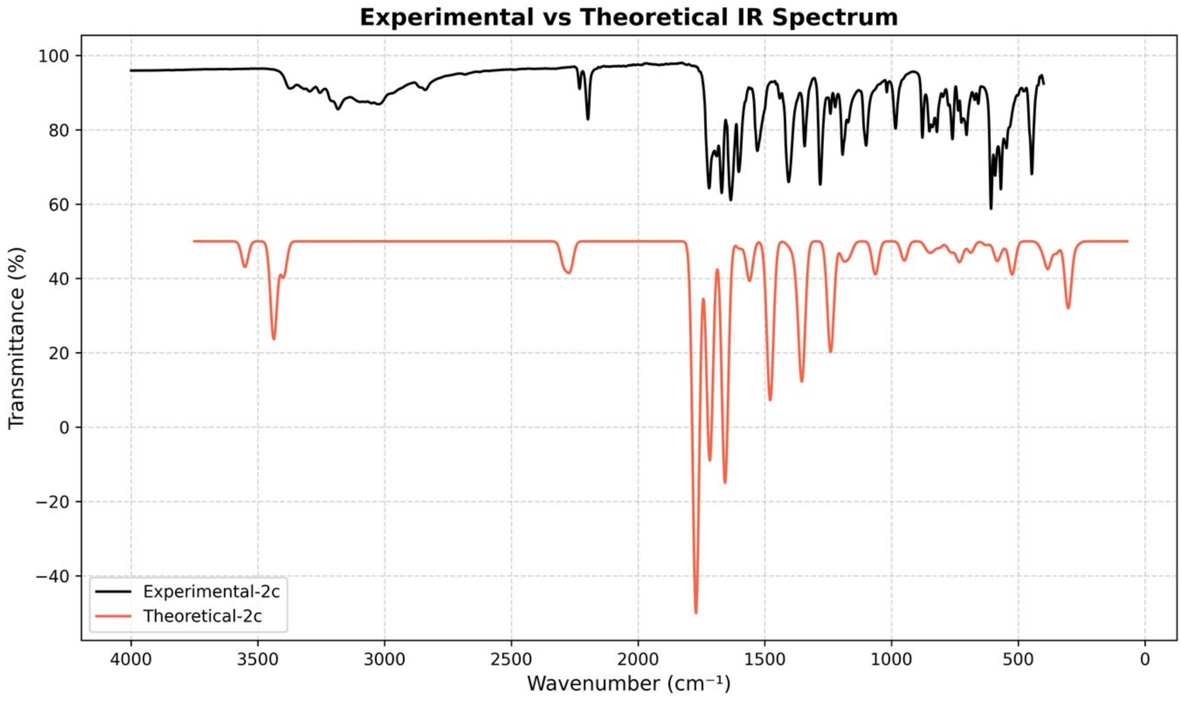

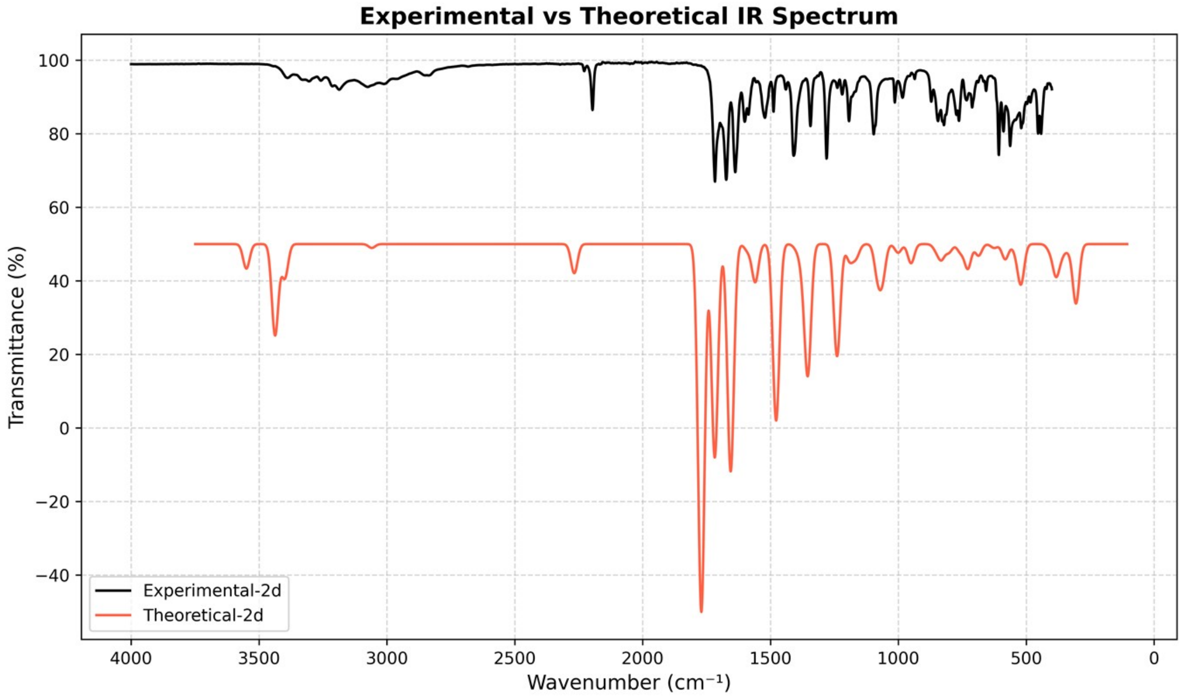

Fig. S28 *Comparative IR spectra of compound* ***2d****: experimental (black) vs theoretical (red) transmittance. Theoretical spectrum was computed using DFT (B3LYP/def2-TZVP) and plotted with a Gaussian convolution to simulate peak broadening.*

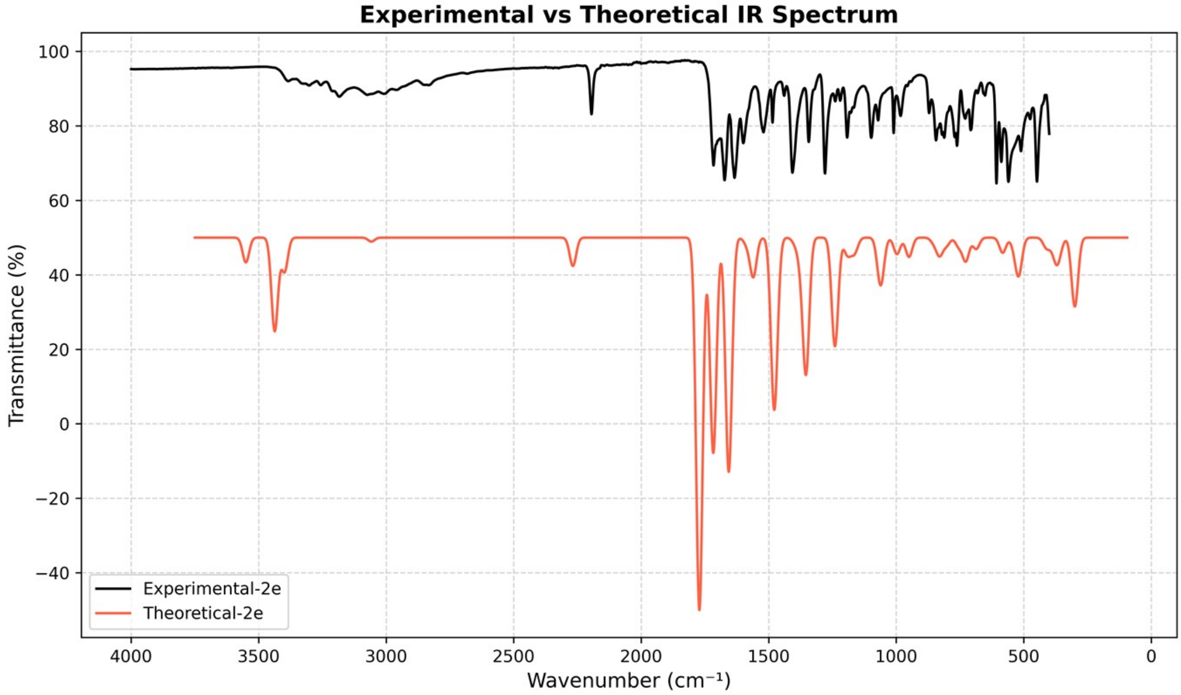

Fig. S29. *Comparative IR spectra of compound* ***2e****: experimental (black) vs theoretical (red) transmittance. Theoretical spectrum was computed using DFT (B3LYP/def2-TZVP) and plotted with a Gaussian convolution to simulate peak broadening.*

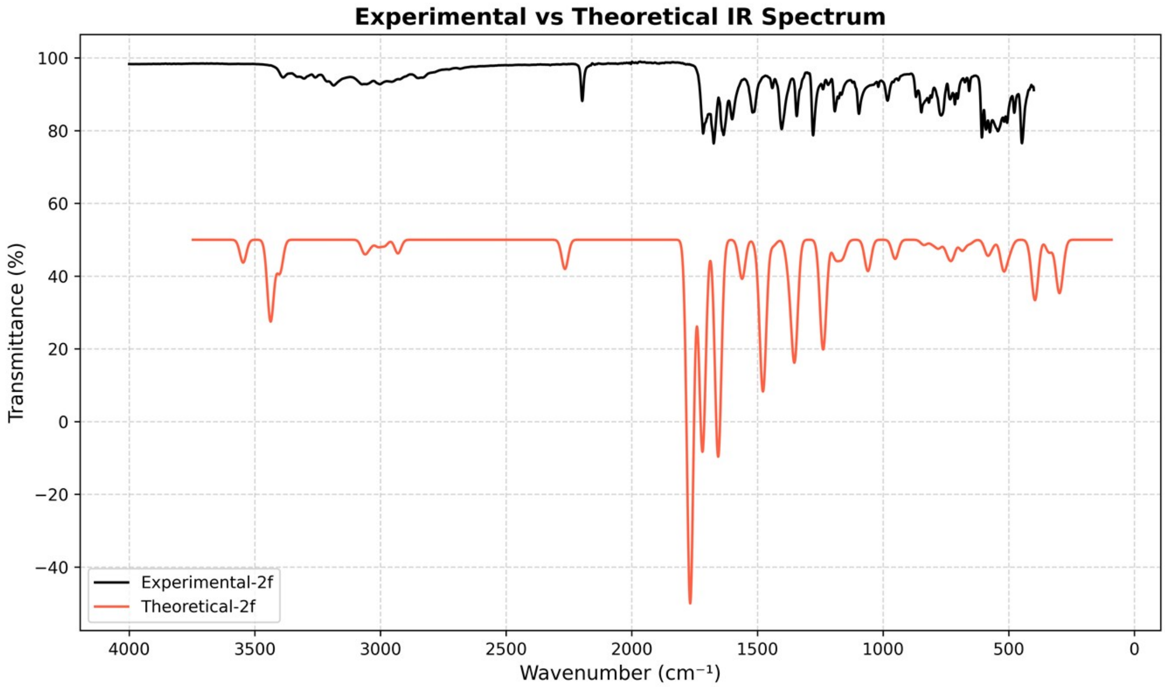

Fig. S30. *Comparative IR spectra of compound **2f**: experimental (black) vs theoretical (red) transmittance. Theoretical spectrum was computed using DFT (B3LYP/def2-TZVP) and plotted with a Gaussian convolution to simulate peak broadening.*

Fig. S31. *Comparative IR spectra of compound* ***2g****: experimental (black) vs theoretical (red) transmittance. Theoretical spectrum was computed using DFT (B3LYP/def2-TZVP) and plotted with a Gaussian convolution to simulate peak broadening.*

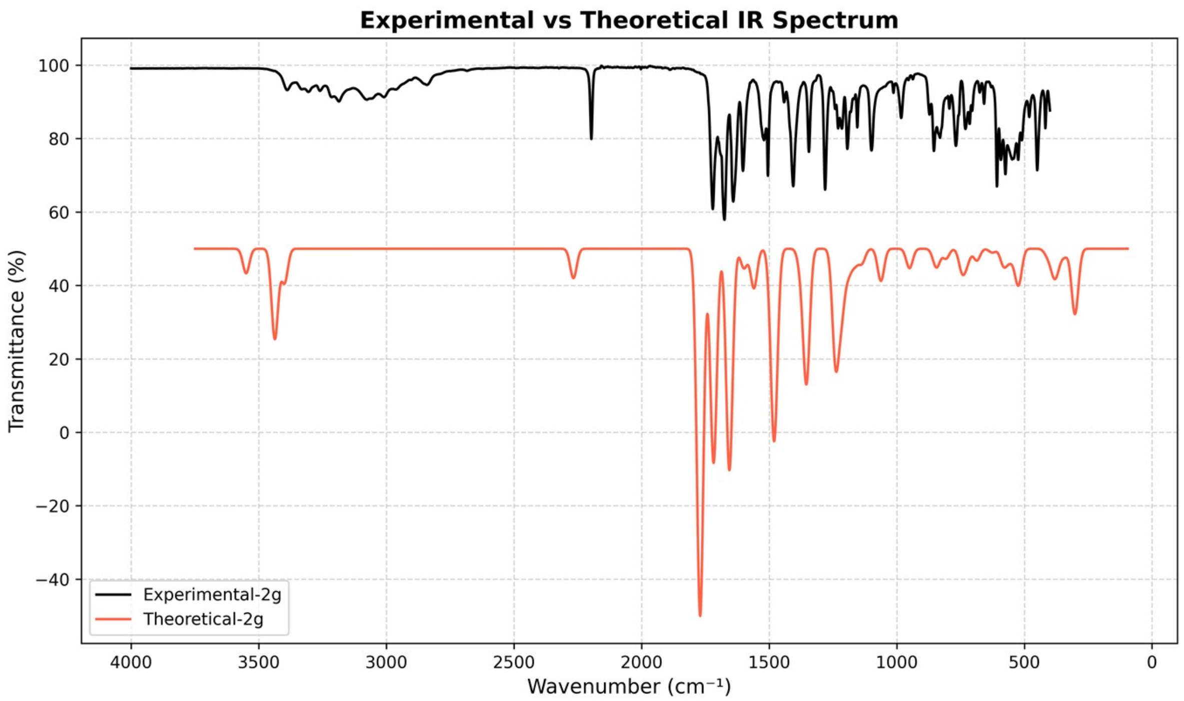

Fig. S32 *Comparative IR spectra of compound* ***2h****: experimental (black) vs theoretical (red) transmittance. Theoretical spectrum was computed using DFT (B3LYP/def2-TZVP) and plotted with a Gaussian convolution to simulate peak broadening.*

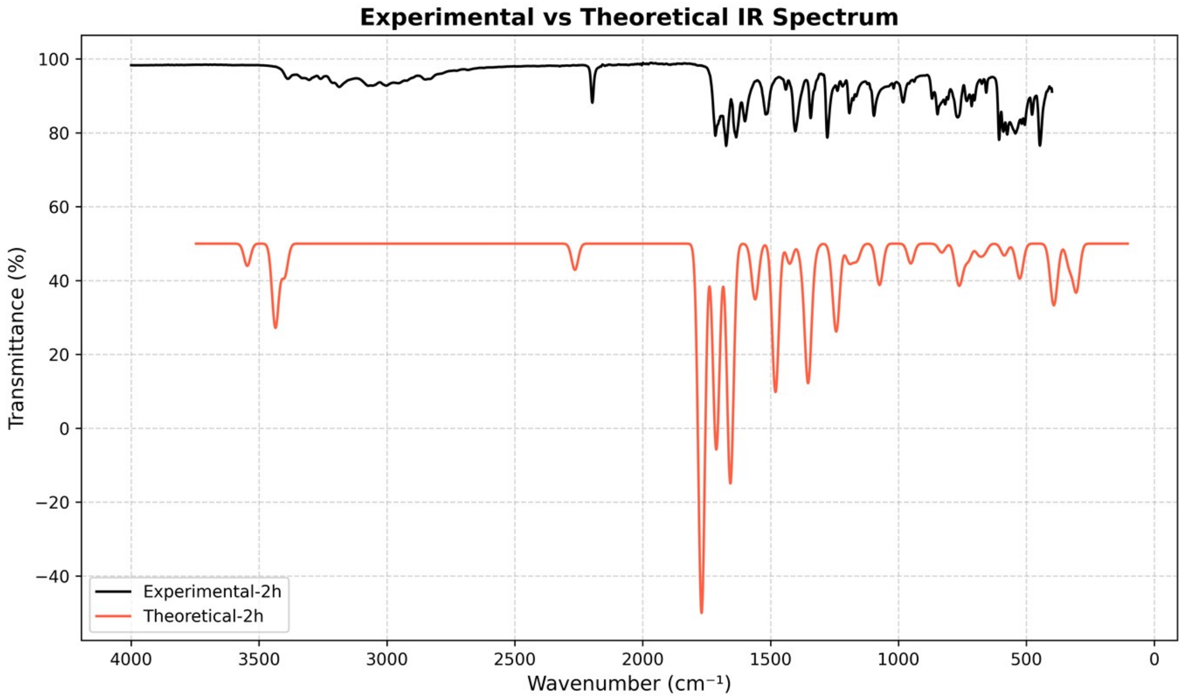

Code	Optimised structure	HOMO	LUMO	MEP
2a	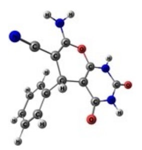	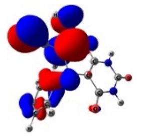	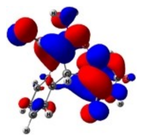	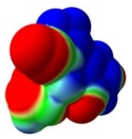
2b	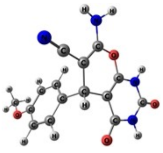	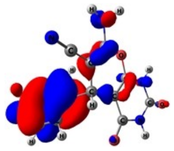	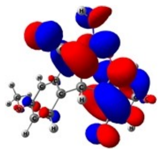	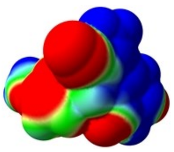
2c	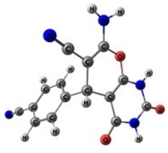	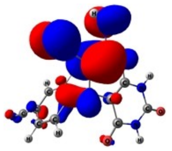	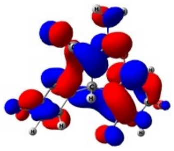	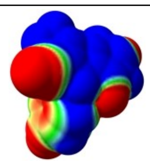
2d	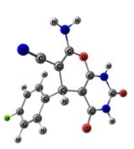	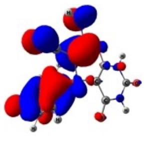	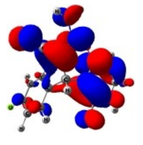	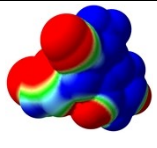
2e	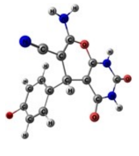	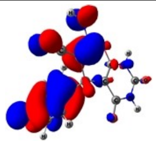	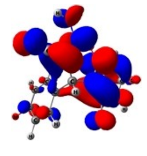	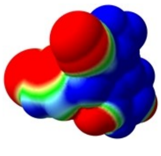
2f	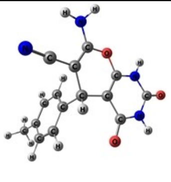	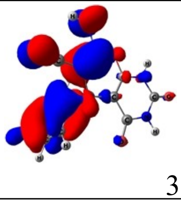	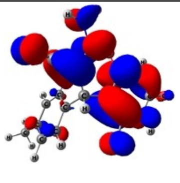	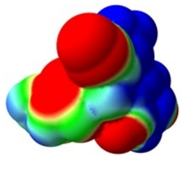

2g	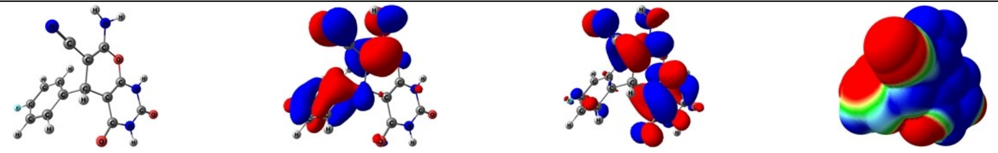
2h	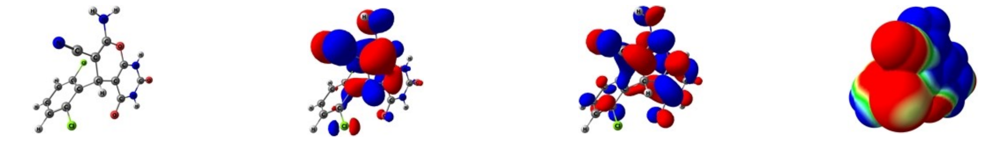
Afatinib	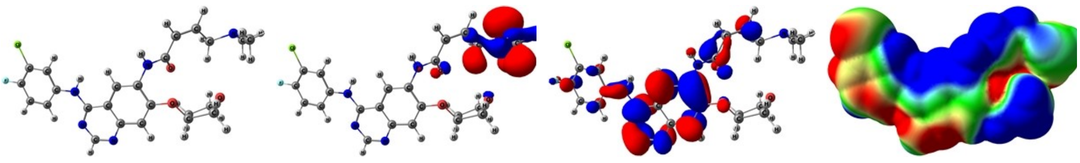

Fig. S33 (HOMO, LUMO) orbitals, Molecular Electrostatic Potential plots (MEP) and optimized structures (2a–2h and afatinib) at B3LYP/def2-TZVP level in gas phase.

Fig. S34. *2D interaction diagrams of ligands docked into the active site of the (PDB ID: 4ZXT).* **(A–I)** represent compounds **2a–2h**, respectively, and **(I)** corresponds to the standard drug Afatinib.

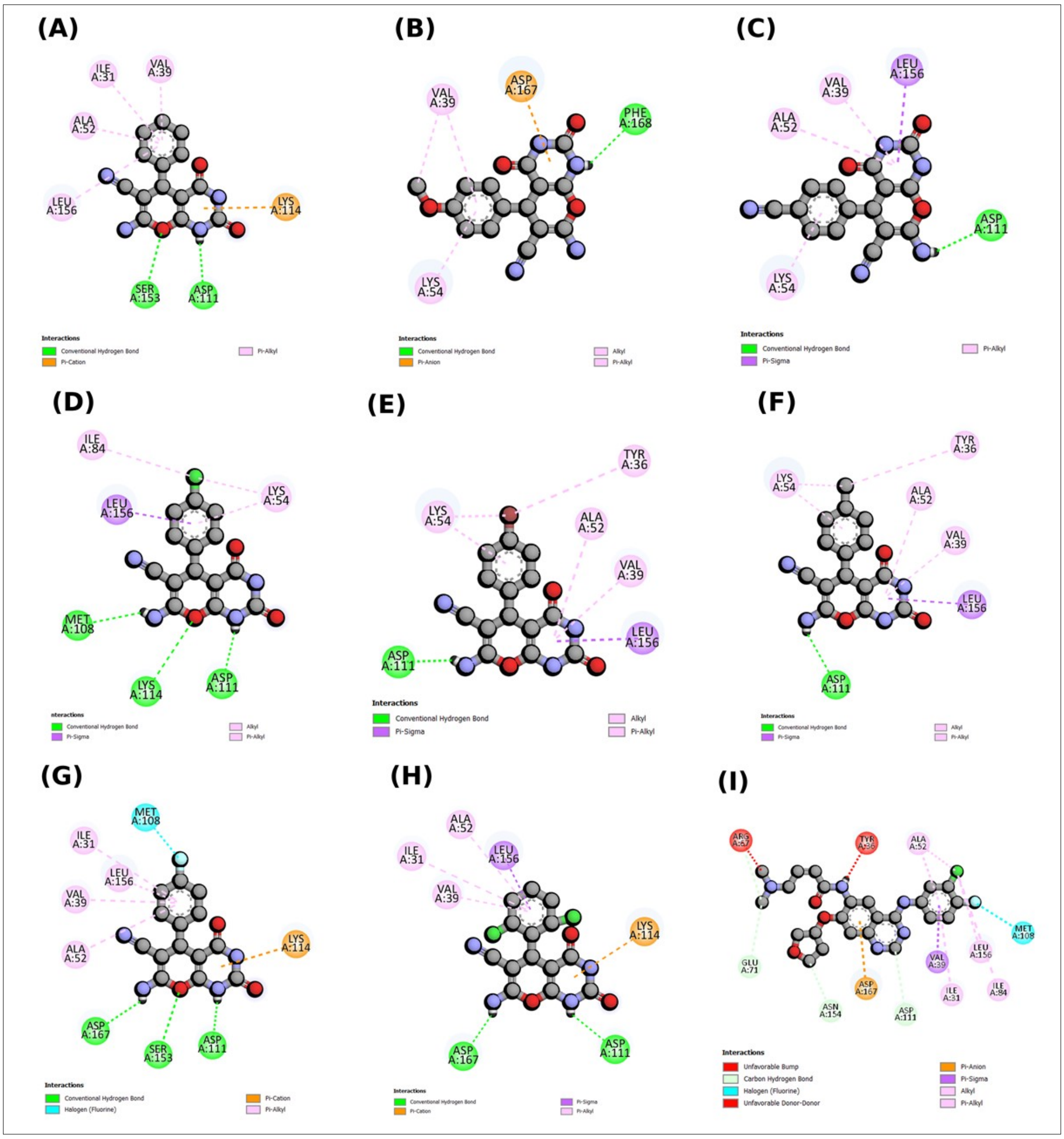
(A)
ILE A:31
VAL A:39
ALA A:52
LEU A:156
LYS A:114
SER A:153
ASP A:111
Interactions
Conventional Hydrogen Bond
Pi-Cation
Pi-Alkyl
(B)
VAL A:39
ASP A:167
PHE A:168
LYS A:54
Interactions
Conventional Hydrogen Bond
Pi-Anion
Alkyl
Pi-Alkyl
(C)
LEU A:156
VAL A:39
ALA A:52
ASP A:111
LYS A:54
Interactions
Conventional Hydrogen Bond
Pi-Sigma
Pi-Alkyl
(D)
ILE A:84
LYS A:54
LEU A:156
MET A:108
LYS A:114
ASP A:111
Conventional Hydrogen Bond
Pi-Sigma
Alkyl
Pi-Alkyl
(E)
TYR A:36
LYS A:54
ALA A:52
VAL A:39
LEU A:156
ASP A:111
Interactions
Conventional Hydrogen Bond
Pi-Sigma
Alkyl
Pi-Alkyl
(F)
TYR A:36
LYS A:54
ALA A:52
VAL A:39
LEU A:156
ASP A:111
Interactions
Conventional Hydrogen Bond
Pi-Sigma
Alkyl
Pi-Alkyl
(G)
MET A:108
ILE A:31
LEU A:156
VAL A:39
ALA A:52
LYS A:114
ASP A:167
SER A:153
ASP A:111
Interactions
Conventional Hydrogen Bond
Halogen (Fluorine)
Pi-Cation
Pi-Alkyl
(H)
ALA A:52
ILE A:31
LEU A:156
VAL A:39
LYS A:114
ASP A:167
ASP A:111
Interactions
Conventional Hydrogen Bond
Pi-Cation
Pi-Sigma
Pi-Alkyl
(I)
ALA A:52
MET A:108
GLU A:71
LEU A:156
VAL A:39
ASP A:167
ASN A:154
ASP A:111
ILE A:31
ILE A:84
Interactions
Unfavorable Bump
Carbon Hydrogen Bond
Halogen (Fluorine)
Unfavorable Donor-Donor
Pi-Anion
Pi-Sigma
Alkyl
Pi-Alkyl

Fig. S35 Linear regression between AutoDock Vina binding scores (kcal mol^{-1}) and cytotoxicity against A549 cells ($\log_{10}$ IC_{50}, µM) for compounds **2a–2h (Afatinib excluded**). A simple least-squares fit gives $R^2 = 0.002$ ($p = 0.914$); Spearman $\rho = 0.048$ ($p = 0.910$).

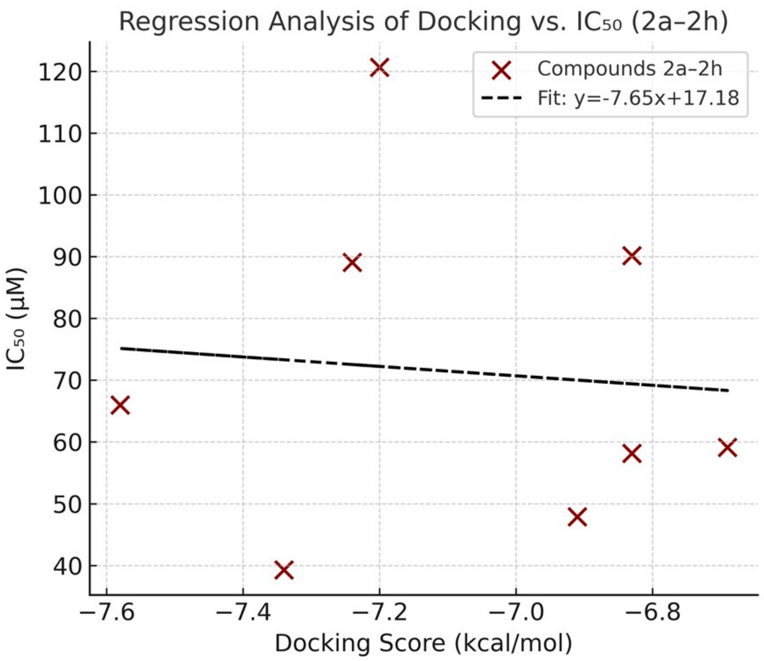

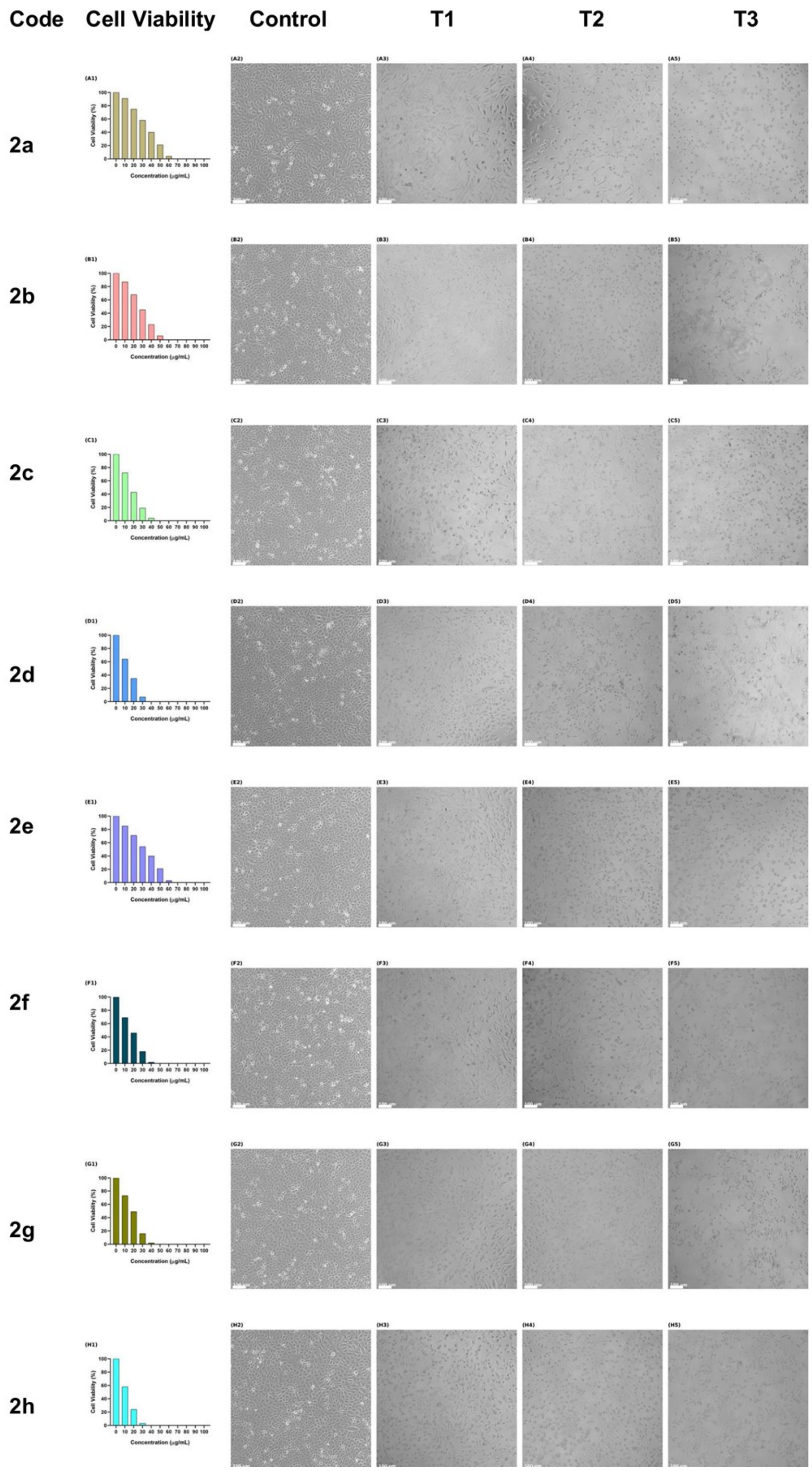

Fig. S36. *MTT assay evaluation of cytotoxic effects of synthesized compounds (**2a–2h**) on* A549 lung carcinoma cells. Each row corresponds to one compound: the first column displays cell viability (%) as a function of concentration (µM); the second column shows the control A549 cells; the next three columns (T1, T2 and T3) represent triplicate treated wells for the corresponding compound. Decreased cell density and morphological damage reflect concentration-dependent cytotoxicity.

Table S1: Experimental and calculated ^{1}H NMR chemical shifts (□ in ppm) of compound **2a** at the B3LYP/def2-TZVP level of theory in DMSO phase.

**Atom**	**B3LYP/def2-TZVP**	**Exp**
H24	4.4	4.2
H22	5.0	7.1
H23	4.5	7.1
H26	7.5	7.2
H27	7.5	7.2
H29	7.5	7.2
H25	7.7	7.3
H28	7.6	7.3
H30	7.0	11.1
H31	7.1	12.1
	**RMSD**	**2.30**

Table S2: Experimental and calculated ^{1}H NMR chemical shifts (□ in ppm) of compound **2b** at the B3LYP/def2-TZVP level of theory in DMSO phase.

**ATOM**	**B3LYP/def2-TZVP**	**Exp**
H33	3.7	3.7
H35	3.7	3.7
H34	4.0	3.7
H24	4.3	4.2
H23	4.4	7.0
H22	4.9	7.0
H28	6.9	7.1
H30	7.0	12.0
H26	7.1	7.1
H31	7.1	11.0
H25	7.3	7.1
H29	7.6	7.1
	**RMSD**	**2.1**

Table S3: Experimental and calculated ^{1}H NMR chemical shifts (□ in ppm) of compound **2c** at the B3LYP/def2-TZVP level of theory in DMSO phase

**Atom**	**B3LYP/def2-TZVP**	**Exp**
H26	4.5	4.3
H25	4.5	7.2
H24	5.1	7.2
H27	7.6	7.4
H30	7.8	7.4
H29	7.9	7.7
H28	8.0	7.7
H31	7.0	11.1
H32	7.1	12.1
	**RMSD**	**2.4**

Table S4: Experimental and calculated ^{1}H NMR chemical shifts (□ in ppm) of compound **2d** at the B3LYP/def2-TZVP level of theory in DMSO phase.

**ATOM**	**B3LYP/def2-TZVP**	**Exp**
H21	5.1	7.2
H22	4.5	7.2
H23	4.4	4.2
H24	7.4	7.2
H25	7.6	7.3
H27	7.5	7.2
H28	7.7	7.3
H29	7.0	11.1
H30	7.1	12.1
	**RMSD**	**2.4**

Table S5: Experimental and calculated ^{1}H NMR chemical shifts (□ in ppm) of compound **2e** at the B3LYP/def2-TZVP level of theory in DMSO phase

**Atom**	**B3LYP/def2-TZVP**	**Exp**
H24	4.4	4.2
H23	4.5	7.2
H22	5.1	7.2
H29	7.4	7.2
H26	7.5	7.2
H28	7.6	7.5
H25	7.6	7.5
H30	7.0	11.1
H31	7.1	12.1
	**RMSD**	**2.4**

Table S6: Experimental and calculated ^{1}H NMR chemical shifts (□ in ppm) of compound **2f** at the B3LYP/def2-TZVP level of theory in DMSO phase

**Atom**	**B3LYP/def2 -TZVP**	**Exp**
H33	2.0	2.3
H32	2.4	2.3
H34	2.5	2.3
H24	4.3	4.2
H23	4.5	7.1
H22	4.9	7.1
H30	7.0	11.1
H31	7.1	12
H25	7.3	7.1
H28	7.4	7.1
H26	7.4	7.1
H29	7.5	7.1
	**RMSD**	**2.1**

Table S7: Experimental and calculated ^{1}H NMR chemical shifts (□ in ppm) of compound **2g** at the B3LYP/def2-TZVP level of theory in DMSO phase

**Atom**	**B3LYP/def2-TZVP**	**Exp**
H24	4.4	4.2
H23	4.5	7.1
H22	5.1	7.1
H30	7.0	11.1
H31	7.1	12.1
H28	7.2	7.1
H26	7.3	7.2
H25	7.5	7.3
H29	7.7	7.3
	**RMSD**	**2.4**

Table S8: Experimental and calculated ^{1}H NMR chemical shifts (□ in ppm) of compound **2h** at the B3LYP/def2-TZVP level of theory in DMSO phase

**Atom**	**B3LYP/def2-TZVP**	**Exp**
H22	5.0	7.43
H23	4.5	7.42
H24	5.8	5.25
H26	7.6	7.2
H27	7.5	7.3
H28	7.5	7.2
H30	7.0	11
H31	7.1	12.06
	**RMSD**	**2.6**

Table S9: Experimental and calculated ^{13}C NMR chemical shifts (δ in ppm) of compound **2a** at the B3LYP/def2-TZVP level of theory in DMSO phase

**Atom**	**B3LYP/def2 -TZVP**	**Exp**
C6	48.9	39.5
C3	71.2	62.7
C13	104.9	92.3
C4	131.7	123.0
C10	137.2	130.6
C9	138.0	131.1
C12	139.5	131.1
C8	140.0	132.1
C11	139.6	132.1
C17	157.1	148.0
C7	160.9	153.3
C14	166.0	156.1
C19	171.3	161.5
C2	175.6	166.3
	**RMSD**	**8.8**

Table S10: Experimental and calculated ^{13}C NMR chemical shifts (□ in ppm) of compound **2b** at the B3LYP/def2-TZVP level of theory in DMSO phase

ATOM	B3LYP/def2-TZVP	Exp
C6	48.1	39.5
C32	62.3	59.6
C3	71.3	63.8
C13	104.9	93.3
C11	117.1	118.2
C9	128.3	119.4
C4	131.8	123.4
C8	140.0	132.9
C12	140.9	132.9
C7	151.0	140.9
C17	157.0	154.0
C14	165.7	156.6
C19	171.3	162.1
C10	173.5	162.7
C2	175.5	167.0
	**RMSD**	**8.2**

Table S11: Experimental and calculated ^{13}C NMR chemical shifts (□ in ppm) of compound **2c** at the B3LYP/def2-TZVP level of theory in DMSO phase

**Atom**	**B3LYP/def2 -TZVP**	**Exp**
C6	49.3	39.5
C3	69.0	61.3
C15	103.6	91.0
C10	118.3	113.2
C4	131.6	122.5
C11	133.3	122.6
C8	141.0	132.2
C14	141.9	132.2
C13	145.2	135.9
C9	146.5	135.9
C19	156.8	153.2
C16	165.7	153.4
C7	167.4	156.3
C21	171.4	161.4
C2	175.4	166.1
	**RMSD**	**9.60**

Table S12: Experimental and calculated ^{13}C NMR chemical shifts (□ in ppm) of compound **2d** at the B3LYP/def2-TZVP level of theory in DMSO phase

ATOM	B3LYP/def2-TZVP	Exp
C5	48.3	34.6
C2	69.3	57.8
C12	104.3	87.5
C3	131.8	118.5
C10	138.6	127.6
C8	140.4	128.8
C7	141.3	129.2
C11	142.3	131.6
C9	149.6	142.6
C16	156.9	148.9
C6	158.9	151.8
C13	165.1	157.0
C18	171.2	159.6
C1	175.7	161.9
	**RMSD**	**11.5**

Table S13: Experimental and calculated ^{13}C NMR chemical shifts (□ in ppm) of compound **2e** at the B3LYP/def2-TZVP level of theory in DMSO phase

Atom	B3LYP/def2-TZVP	Exp
C6	48.5	39.5
C3	69.4	62.5
C13	104.0	92.2
C4	131.7	123.2
C10	149.8	124.0
C9	141.3	133.9
C12	141.6	133.9
C8	142.7	135.4
C11	143.1	135.4
C17	156.9	147.8
C7	160.1	153.7
C14	165.2	156.6
C19	171.3	161.8
C2	175.3	166.6
	**RMSD**	**10.7**

Table S14 Experimental and calculated ^{13}C NMR chemical shifts (in ppm) of compound **2f** at the B3LYP/def2-TZVP level of theory in DMSO phase

**Atom**	**B3LYP/def2-TZVP**	**Exp**
C27	28.2	24.8
C6	48.4	39.5
C3	71.5	63.3
C13	105.0	92.8
C4	131.8	123.4
C11	138.5	131.4
C8	139.2	131.4
C12	139.6	133.1
C9	139.8	133.1
C10	151.5	139.9
C17	157.1	145.4
C7	157.2	153.7
C14	165.9	156.4
C19	171.3	161.8
C2	175.5	166.7
	**RMSD**	**8.6**

Table S15: Experimental and calculated ^{13}C NMR chemical shifts (□ in ppm) of compound **2g** at the B3LYP/def2-TZVP level of theory in DMSO phase

**Atom**	**B3LYP/def2-TZVP**	**Exp**
C6	48.3	weak
C3	69.7	58.8
C13	104.5	88.3
C11	124.0	114.5
C9	126.2	115.3
C4	131.8	119.1
C8	141.6	128.8
C12	142.5	129.7
C7	156.4	140.3
C17	156.9	149.5
C14	165.0	152.2
C19	171.3	157.6
C2	175.4	160.3
C10	178.5	162.4
	**RMSD**	**12.6**

Table S16: Experimental and calculated ^{13}C NMR chemical shifts (□ in ppm) of compound **2h** at the B3LYP/def2-TZVP level of theory in DMSO phase.

**Atom**	**B3LYP/ def2- TZVP**	**Exp**
C6	45.2	32.1
C3	65.6	54.1
C13	100.8	86.1
C4	130.9	118.5
C9	139.7	128.6
C10	139.8	129.3
C11	141.7	130.3
C7	151.4	134.1
C12	152.7	135.7
C8	153.8	135.9
C17	156.9	149.5
C14	167.6	153.0
C19	170.9	158.7
C2	176.4	162.1
	**RMSD**	**12.7**

Table S17 Comparative overview of reported catalytic systems for the synthesis of pyrano[2,3-d]pyrimidinone derivatives under different reaction conditions, along with the present Ag_2WO_4-catalyzed protocol.

Entry	Catalyst	Condition	Time/Yield (%)	Reference
1.	γ-Fe_2O_3@SiO_2@[Bis-APTES]Cl_2-NPs	EtOH, 70 ^{O}C	30min/95	(ref. 6)
2.	Fe_3O_4@HWSS@Ag°	EtOH, 75 ^{O}C	20-30/80-95	(ref. 7)
3.	Mn-ZIF-8@$ZnTiO_3$	EtOH/H_2O, 70 ^{O}C	15-30min/95	(ref. 8)
4.	DABCO	H_2O, RT,	5 min/94	(ref. 9)
5.	PPI	H_2O/reflux	20 min/94	(ref. 10)
6.	Cellulose based-Nanocomposite	H_2O, RT	30 min/ 96	(ref. 11)
7.	Fe_3O_4@SiO_2@$(CH_2)_3$-Urea-SO_3H/HCl	Solvent free, 60 ^{O}C	immediately/98	(ref. 12)
8.	LDH@TRMS@NDBD@$Cu(NO_3)_2$,	Solvent-free,RT	5 min/97	(ref. 13)
9.	CS-ZnONPs	H_2O, MW	3 min/ 95	(ref. 14)
10.	2-aminopyridine	EtOH/Reflux	10 min/92	(ref. 15)
11.	**Ag_2WO_4**	**EtOH/H_2O, 70 ^{O}C**	**5 min/95**	**This work**

Table S 18 Structure-Activity Relationship (SAR) analysis of synthesized pyrano[2,3-d]pyrimidinone derivatives (2a-2h) against the A549 cell line, correlating IC_{50} values, physicochemical descriptors, and docking scores with substituent effects

Entry	Substituent	IC50 (µM)	TPSA (Å²)	log P	Docking Score (kcal/mol)	Activity Class	SAR Observation
2a	None (Simple scaffold)	120.65	124.76	1.13	-7.20	Weakly active	Parent scaffold shows minimal activity; limited substitution reduces binding.
2b	-OMe	89.09	133.99	1.40	-7.24	Moderately active	Electron-donating OMe group improves activity modestly via enhanced solubility.
2c	-CN	59.13	148.55	1.35	-6.69	Moderately active	Strong electron-withdrawing CN enhances polarity but reduces docking affinity.
2d	-Cl	47.91	124.76	1.40	-6.91	Active	Chloro substitution increases hydrophobic contacts, enhancing activity.
2e	-Br	90.17	124.76	1.50	-6.83	Weakly active	Bulky Br substitution decreases

							bioactivity likely due to steric hindrance.
2f	-Me	58.15	124.76	1.39	-6.83	Moderately active	Methyl group contributes to hydrophobic stabilization, improving activity.
2g	-F	65.97	124.76	1.22	-7.58	Moderately active	Fluoro substitution provides better docking affinity but moderate cell activity.
2h	-Cl, -Cl (ortho dichloro)	39.29	124.76	1.47	-7.34	Most active	Ortho-dichloro substitution improves hydrophobic packing and binding stability.
Afatinib	Reference drug	1.4	87.22	3.95	-8.01	Highly active	Strong binding and potency validate reference standard.
